# Analysis of the Roles of the Arabidopsis nMAT2 and PMH2 Proteins Provided with New Insights into the Regulation of Group II Intron Splicing in Land-Plant Mitochondria

**DOI:** 10.3390/ijms18112428

**Published:** 2017-11-17

**Authors:** Michal Zmudjak, Sofia Shevtsov, Laure D. Sultan, Ido Keren, Oren Ostersetzer-Biran

**Affiliations:** 1 Department of Plant and Environmental Sciences, The Alexander Silberman Institute of Life Sciences, The Hebrew University of Jerusalem, Givat-Ram, Jerusalem 91904, Israel; michalzm@gmail.com (M.Z.); sofia.shevtsov@gmail.com (S.S.); laure.sultan@gmail.com (L.D.S.); Ido.Keren@stonybrook.edu (I.K.); 2 Department of Biochemistry and Cell Biology, State University of New York, Stony Brook, NY 11794, USA

**Keywords:** group II introns, splicing, maturases, RNA helicases, mitochondria, Arabidopsis, angiosperms

## Abstract

Plant mitochondria are remarkable with respect to the presence of numerous group II introns which reside in many essential genes. The removal of the organellar introns from the coding genes they interrupt is essential for respiratory functions, and is facilitated by different enzymes that belong to a diverse set of protein families. These include maturases and RNA helicases related proteins that function in group II intron splicing in different organisms. Previous studies indicate a role for the nMAT2 maturase and the RNA helicase PMH2 in the maturation of different pre-RNAs in Arabidopsis mitochondria. However, the specific roles of these proteins in the splicing activity still need to be resolved. Using transcriptome analyses of Arabidopsis mitochondria, we show that nMAT2 and PMH2 function in the splicing of similar subsets of group II introns. Fractionation of native organellar extracts and pulldown experiments indicate that nMAT2 and PMH2 are associated together with their intron-RNA targets in large ribonucleoprotein particle in vivo. Moreover, the splicing efficiencies of the joint intron targets of nMAT2 and PMH2 are more strongly affected in a double *nmat2/pmh2* mutant-line. These results are significant as they may imply that these proteins serve as components of a proto-spliceosomal complex in plant mitochondria.

## 1. Introduction

Plants are able to regulate and coordinate their energy demands during particular growth and developmental stages. These activities require complex cellular signaling between the nucleus and the mitochondrial genome (i.e., mitogenome (mtDNA)) (reviewed by e.g., [1,2]). Although mitochondria contain their own genetic material, encoding some proteins and structural RNAs, the vast majority of mitochondrial proteins are encoded by nuclear loci, and are imported from the cytosol post-translationally [3,4,5,6]. In fact, both the ribosomes and the respiratory machinery are composed of proteins encoded by both nuclear and organellar loci (see e.g., [7,8]). These necessitate complex mechanisms to allow the stoichiometric accumulation of subunits encoded by the two physically remote genetic compartments, through different biosynthetic pathways [9]. 

The expression of the mtDNA in plants is regulated mainly at the post-transcriptional level. High-throughput RNA-seq analyses provided with new insights into the complexity of RNA metabolism in plant mitochondria and indicated that the regulation of mtRNA processing plays a critical role in plant organellar gene-expression [1,10,11,12,13,14,15]. The importance of post-transcriptional regulation in plant mitochondria is further reflected by the extended half-lives of many of the organellar transcripts, and the fact that their translation seems uncoupled from their transcription [10,16,17,18]. One of the most notable features of plant mitochondria gene-expression involves the splicing of numerous intervening sequences (introns) that must be removed from the coding-regions post-transcriptionally [10,11,12,13,14,15]. The processing of the organellar pre-RNAs is essential for respiratory activities and relies on the activities of many different cofactors which belong to a diverse set of RNA-binding protein families. 

The mitochondrial introns in angiosperms are classified as group II-type and are found mainly within protein-coding genes [14,19,20,21]. In Arabidopsis mitochondria, these include 23 group II-type introns found within the complex I *nad1*, *nad2*, *nad4*, *nad5* and *nad7* subunits, the cytochrome c biogenesis factor C (*ccmFc*), the *cox2* subunit of complex IV and the ribosomal proteins *rpl2* and *rps3* (Table 1 and [22]). Introns belonging to this class are large catalytic RNAs (and in some cases also mobile genetic elements) which are excised from the precursor RNAs by two sequential transesterification reactions, involving the release of the intron as an RNA lariat (reviewed by, e.g., [21]). Based on structural similarities and the catalytic activities, it is generally accepted that the nuclear spliceosomal introns have originated from bacterial group II-related introns, which were introduced to the eukaryotic genomes by the bacterial ancestor of the mitochondrion [23]. Although some group II introns are able to catalyze their own excision in vitro, the splicing of group II introns under native conditions (in vivo) is assisted by protein cofactors. In bacteria and yeast mitochondria, this involves proteins that are encoded within the introns themselves (i.e., Intron Encoded Proteins, IEPs; or maturases) [24,25,26,27]. Genetic and biochemical data indicate that group II intron-encoded maturases bind with high affinity and specificity towards the introns in which they are encoded from, and facilitate splicing by assisting the folding of the RNAs into their catalytically active forms [15,24,25,26,27].

The mitochondrial introns in plants are expected to have evolved from maturase-encoding group II intron RNAs. However, throughout the evolution of land-plants, these have diverged considerably from their related bacterial ancestors, such as they lack many sequence elements that are considered essential for the splicing activity, and also lost the vast majority of their related maturase ORFs [14,19,28,29]. Several of the mitochondrial introns in plants are transcribed as individual pieces that are assemble in *trans* through base-pairing interactions, to form a splicing-competent structure. Interestingly, this situation is reminiscent of the *trans*-interaction of spliceosomal RNAs with pre-mRNAs (reviewed in e.g., [14]). In Arabidopsis, the *trans*-spliced introns include the first and third introns in the NADH-dehydrogenase subunit 1 (i.e., *nad1* introns 1 and 3), *nad2* intron 2, and *nad5* introns 2 and 3 [22]. Due to their degenerate nature and the fact that the organellar introns have also lost their cognate maturase factors, both the *cis*- and *trans*-splicing reactions of plant mitochondrial group II introns rely upon the activities of different catalytic enzymes, most of which are encoded by nuclear loci (reviewed by [14,15,19]). However, the specific roles of these factors in the splicing activity is still under investigation. 

Genetic screens have led to the identification of different splicing cofactors in plant mitochondria [12,14,15,30]. These include proteins which are closely related to maturases encoded within group II introns (i.e., the mitochondrial MatR encoded in *nad1* i4 and four nuclear-encoded maturase proteins, nMATs 1 to 4) [15,31,32,33,34,35], and at least two RNA helicases (i.e., PMH2 and ABO6) that are encoded in the nucleus and imported into the organelles [36,37,38,39]. Maturases and RNA helicases belong to ancient groups of RNA-binding proteins that facilitate intron splicing and were most likely inherited directly from the bacterial symbiont [14,15,21]. Other proteins that influence the splicing of group II introns in plant organelles include pentatricopeptide repeat (PPR) proteins, chloroplast RNA maturation (CRM)-related factors (i.e., the mCSF1 protein), members of the plant organellar RNA recognition (PORR) family, mitochondrial transcription termination factor (mTERF) related proteins and several other factors, which are seemingly unique to eukaryotes and are thus expected to evolve from the host genomes to function in organellar group II intron splicing in plants (reviewed in, e.g., [12,14,15,30]). Some of the proteins, such as nMAT2 [33], PMH2 [37] and mCSF1 [40], are required for the processing of a larger set of introns, while other factors such as PPR proteins (see, e.g., [41,42,43,44,45,46,47,48,49]) and mTERF15 [50] appear to be more specific, influencing the splicing of a single or only a few group II introns [14]. 

Here, we examined the functions of the nMAT2 maturase, encoded by the At5g46920 locus, and the RNA helicase PMH2 (also known as AtRH53), encoded by the At3g22330 gene, in mitochondrial RNA (mtRNA) metabolism in Arabidopsis plants. Published data have provided with evidence that the splicing of some group II introns (i.e., *cox2* i1 and *nad1* i2) in Arabidopsis mitochondria require both nMAT2 and PMH2 [33,37]. These results are interesting as they may imply that nMAT2 and PMH2 cooperate in the splicing of various organellar introns, in a similar manner to the roles of spliceosomal factors in the splicing of multiple introns in the nucleus. However, these studies which aimed to identify the intron targets of nMAT2 and PMH2 could not provide with definitive experimental data regarding to the roles of these proteins in the splicing of each of the 23 introns in Arabidopsis mitochondria [22]. Furthermore, although the main functions of nMAT2 and PMH2 are supporting RNA splicing, recent data indicate that some splicing cofactors may also affect other RNA processing events in plants mitochondria [41,51,52,53]. The effects of lowering the expression of nMAT2 and PMH2 on mitochondria functions, organelle gene-expression and the physiology of single *nmat2, pmh2* and double *nmat2*/*pmh2* mutant-lines in Arabidopsis plants are discussed.

## 2. Results

### 2.1. The Topology of nMAT2 and PMH2 Proteins

Proteins that interact with group II introns to facilitate their splicing are divided into two main categories, based on their topology and predicted evolutionary origins [14,15,54,55,56]: (*i*) proteins that are encoded within the introns themselves (i.e., Intron Encoded Proteins, IEPs; or maturases); and (*ii*) various ‘*trans*-acting’ factors that function in the splicing of group II introns. These proteins typically contain motifs that are identified as nucleic-acid binding sites, and, in some cases, also seem to harbor regions likely to mediate protein–protein interactions (see, e.g., [14,15]). Here, we focus on two key plant members of the maturase and RNA helicase families, nMAT2 and PMH2, which have key roles in group II introns splicing in different biological systems [14,15]. While nMAT2 (At5g46920; Figures S1A and S2) is identified as a mitochondria-localized nuclear-encoded maturase protein [31,33], the PMH2 protein (At3g22330; Figures S1B and S2) is closely related to DEAD-box RNA helicases [36,37]. 

Maturases encoded within group II introns contain several functional domains that are required for both the splicing activity and intron mobility [15,28,57]. These include a retroviral-like reverse transcriptase (RT) domain and a carboxyl-termini region with a sequence similarity to DNA endonucleases (D/En). Domain search analysis, using SMART [58] and Conserved Domain Database (CDD) [59] servers, indicate that nMAT2 (735 amino acids) harbors the consensus fingers-palm (amino acids 104–461) and thumb (X) motifs (amino acids 486–625), typical to reverse-transcriptases found in group II intron maturases, but lacks the C-terminal D/En domain (Figures S1 and S2). Based on these data and previous reports [31,34,60], nMAT2 is categorized as a type-I maturase (i.e., maturases that retain the RT domain but lack the canonical D/En motif). Similar to other mitochondrial proteins that are encoded within the nucleus, nMAT2 harbors a short (i.e., 12 aa) N-terminal sequence which represents the mitochondrial targeting signal (Figures S1 and S2). 

To gain structural insight, we used the Protein Homology/Analogy Recognition Engine (Phyre) server [61] to model the 3D-structure of nMAT2 protein (Figure S3A). Based on the “in silico” analysis, the nMAT2 protein shares a significant homology with the *L. lactis* LtrA maturase (c5g2xC; confidence 100%) [31,34,60], as well with the RT domains of P21 maturase (c5hhlA; confidence 100), a telomerase (c3du6A; confidence 99.7) and a retroviral-type RT region of HIV-1 (c1rthA; confidence 98.2).

Characterization of the deduced amino acid sequence of the Arabidopsis PMH2 protein (616 aa), using the PHYRE [61] and ROBETTA [62] servers, indicates that PMH2 shares high similarities with other RNA helicases, as the ATP-dependent RNA helicases PRP5 (c4ljyA; confidence 100) and PRP28 (c4w7sA; confidence 100), which are required for the assembly of the spliceosome and the splicing of spliceosomal introns within the nucleus [63]. Analysis of functional domains, implemented in the CDD [59] and SMART [58] servers, revealed that in addition to the predicted N-terminal mitochondrial targeting signal (25 aa long), PMH2 also harbors the two conserved motifs typical to DEAD-box RNA helicases: a DEXD-box (amino acids 124–326) domain, which also include the consensus ATP binding site (amino acids 148–154), and a C-terminal region with a great similarity to the “Helicase C-terminal domain” (amino acids 364–443) (Figures S1 and S2).

### 2.2. Mutations in the nMAT2 and PMH2 Gene-Loci Affect the Steady-State Levels of Many mRNAs in Arabidopsis Mitochondria

The homology of nMAT2 and PMH2 with known maturases and RNA-helicases, respectively [33,36,37], supports a role for these proteins in the processing of mtRNAs in plants. Accordingly, in our prior work, we showed that nMAT2 functions in the splicing of at least three mitochondrial introns, including *cox2* i1, *nad1* i2 and *nad7* i2 [33]. The RNase protection and Northern blot analyses also indicated to perturbations in the processing of several other pre-RNAs in *nmat2* mutants [33]. Likewise, using multiplex RT-PCR analyses, Köhler et al. [37] showed that mutations in the *PMH2* locus led to reduced splicing efficiencies of many introns in Arabidopsis mitochondria. However, the identity of the complete sets of intron targets and the specific roles of nMAT2 and PMH2 in the splicing of group II introns still needs to be resolved. It also remains possible that these factors will participate in aspects of RNA metabolism other than group II introns splicing [33,37].

**Figure 1:**
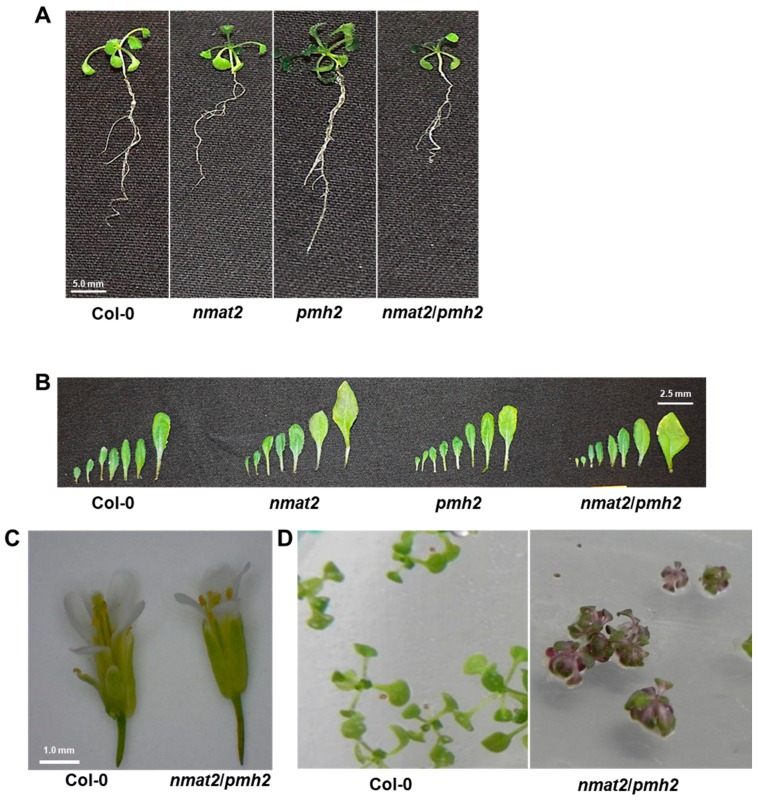
Plant phenotypes associated with *nmat2*, *pmh2* and the double mutant line. The effects of loss of nMAT2 and PMH2 on seedling development (**A**), leaf (**B**) and flower (**C**) morphologies of Arabidopsis wild-type (Col-0) and knockout/knockdown mutant lines. The effects of higher light intensity (i.e., 300 μE m-2 sec-1) on three-week-old *nmat2/pmh2* plants is indicated in panel (**D**). Bars represent 5.0 mm in panel (**A**), 2.5 mm in panel (**B**) and 1.0 mm in panel (**C**).

To establish the roles of nMAT2 and PMH2 in mitochondrial RNA metabolism we analyzed several Arabidopsis plants affected in the *mMAT2* and *PMH2* gene-loci. These including a T-DNA knockout *nmat2* mutant (SALK-line 064659), a *pmh2* knockdown T-DNA line, which is strongly affected in the expression of PMH2 (i.e., SAIL-628C06, [37]), and a double mutant line, *nmat2/pmh2*, that is affected in both these loci (for some unknown reasons, we failed to establish a double mutant line using *nmat2* and a knockout *pmh2* mutant, the SALK-line 056387 [37]) (Figures S1 and S2). Analysis of the phenotypes associated with *nmat2* and *pmh2* mutants indicate that they are both hardly distinguishable from those of wild-type plants, grown under similar conditions (see “Materials and Methods”) (Figure 1) [34,37]. To gain more insights into the roles of nMAT2 and PMH2 in mtRNA metabolism, and to address specific changes in the levels of the different mRNAs and pre-RNAs in Arabidopsis mitochondria, we used transcriptome analyses by quantitative reverse transcription PCRs (RT-qPCR) (see, e.g., [34,35,40,60,64]), of wild-type plants and *nmat2*, *pmh2* and *nmat2/pmh2* mutants. 

**Figure 2:**
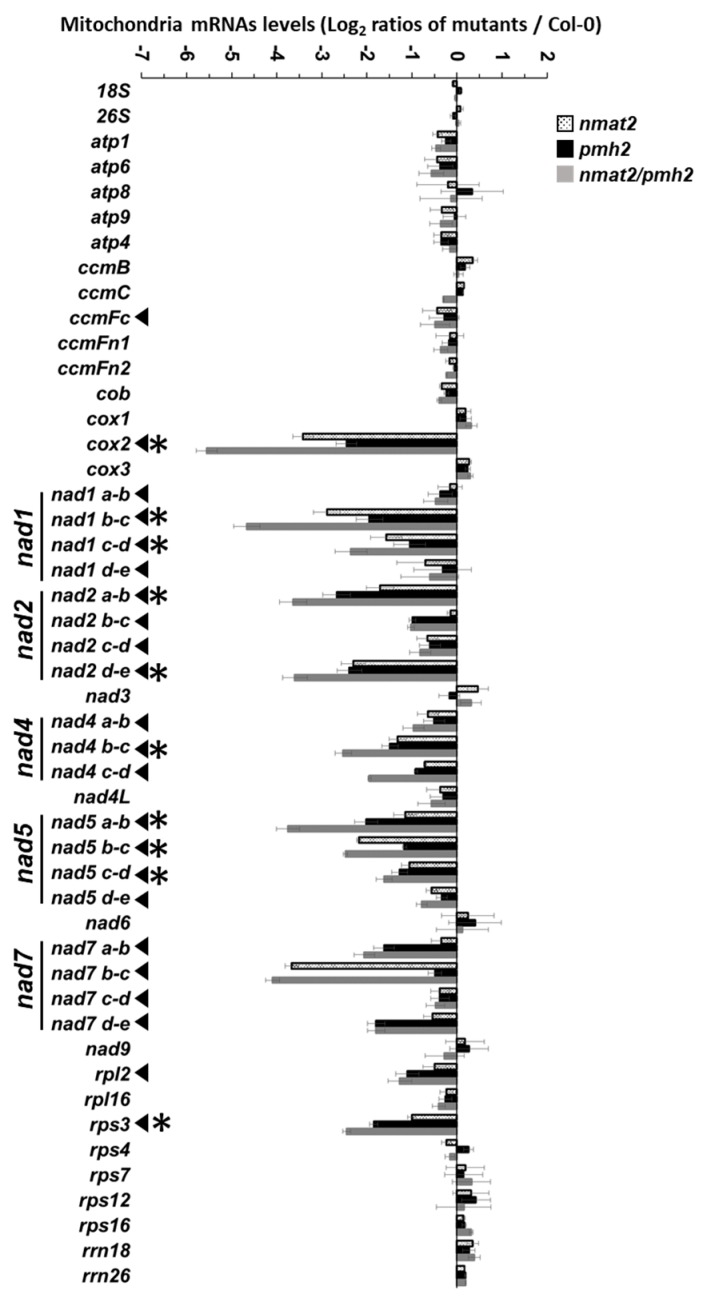
Transcript abundance of mitochondrial mRNAs in *nmat2*, *pmh2* and *nmat2/pmh2* mutants. Transcriptome analyses of mitochondria mRNAs levels in Arabidopsis plants by RT-qPCR was preformed essentially as described previously [35,40,60] (see also in “Materials and Methods”, RNA extraction and analysis). RNA extracted from three-week-old seedlings of wild-type (Col-0) and mutant plants was reverse-transcribed, and the relative steady-state levels of cDNAs corresponding to the different organellar transcripts were evaluated by qPCR with primers which specifically amplified mRNAs (see “Materials and Methods”). The histogram shows the relative mRNAs levels (i.e., log2 ratios) in mutant lines versus those of wild-type plants. Arrows indicate to genes that are interrupted by group II intron sequences in Arabidopsis mitochondria [22], while asterisks indicate to transcripts where the mRNA levels were reduced in both *nmat2* and *pmh2* lines. The values are means of five biological replicates (error bars indicate one standard deviation).

**Figure 3:**
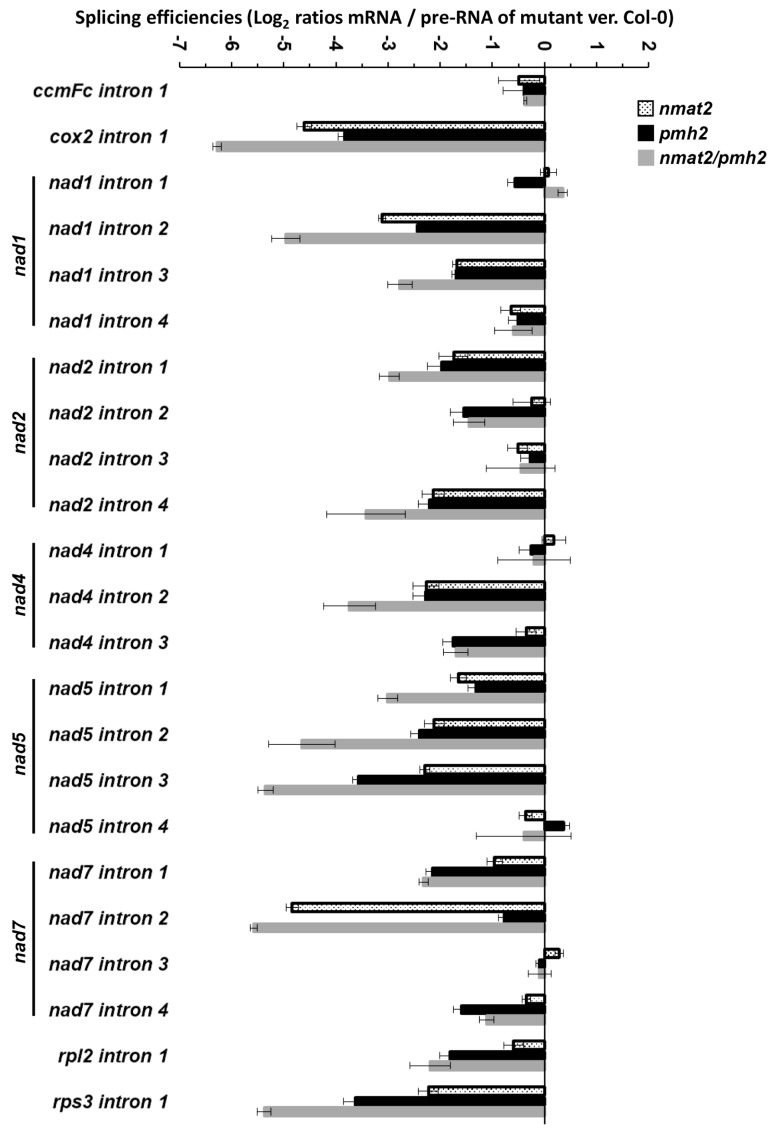
Splicing efficiencies and abundance of mitochondrial transcripts in *nmat2*, *pmh2* and *nmat2/pmh2* mutants. Transcriptome analyses of mitochondria gene-expression in Arabidopsis plants by RT-qPCR were preformed essentially as described previously [34,35,49] (see also in “Materials and Methods”, RNA extraction and analysis). RNA extracted from three-week-old seedlings of wild-type (Col-0) and mutant plants was reverse-transcribed, and the relative steady-state levels of cDNAs corresponding to the different organellar transcripts were evaluated by qPCR with primers which specifically amplified pre-RNAs and mRNAs (see “Materials and Methods”). The histogram shows the splicing efficiencies as indicated by the log2 ratios of pre-RNA to mRNA transcript abundance in mutant lines compared with those of wild-type plants. The values are means of three biological replicates (error bars indicate one standard deviation).

Different from canonical maturases encoded within group II introns, the RNA profiles by RT-qPCR indicated that nMAT2 functions in the maturation of numerous different transcripts in Arabidopsis mitochondria. In *nmat2*, notable reductions in mRNA levels were seen in transcripts which correspond to different exons in complex I *nad1* (i.e., exons b–c and exons c–d), *nad2* (exons a–b and d–e), *nad4* (exons b–c), *nad5* (exons a–b, b–c and c–d) and *nad7* (exons b–c) genes, the *cox2* subunit of complex IV and the ribosomal *rps3* gene (Figure 2), that are all interrupted by group II intron sequences in Arabidopsis mitochondria [22]. Lower steady-state levels of *cox2*, *nad1* and *nad7* transcripts in *nmat2* coincide with the data shown in Keren et al. [34]. However, reduced transcript levels corresponding to *nad2*, *nad4*, *nad5* and *rps3* may indicate additional splicing defects in *nmat2*, as was previously suggested by the RNase protections and Northern blot analyses [34]. The expression of *ccmFc*, which is also interrupted by a group II intron sequence, was not (or only slightly) affected in *nmat2*. Similarly, the levels of mRNAs that correspond to mitochondrial genes which lack group II introns (i.e., “intron-less” transcripts) were not significantly affected by the mutation in the *nMAT2* gene-locus (Figure 3). These included *cox1* and *cox3* subunits of complex IV, different subunits of the ATP synthase enzyme (i.e., complex V), various cytochrome C biogenesis and maturation (*ccm*) factors, and many ribosomal genes (other than *rps3*), which their mRNAs levels in *nmat2* were comparable to those seen in wild-type plants (Figure 2).

The RNA profiles of *pmh2* mutants were similar to those previously reported in Köhler et al. [37]. These analyses indicated to reduced mRNA levels of multiple genes, including *cox2*, *nad1* exons b–c and c–d, *nad2* exons a–b, b–c and d–e, *nad4* exons a–b, b–c and c–d, *nad5* exons a–b, b–c, c–d, *nad7* exons a–b, as well as *rpl2* and *rps3* mRNAs (Figure 2). Similarly to *nmat2* mutant, the accumulation of “intron-less” transcripts in the mitochondria was not significantly affected in the *pmh2* mutant (Figure 2). Small changes in the abundances of various mtRNAs in *nmat2* and *pmh2* plants (Figure 2 and [37]) may be caused by compensatory effects during transcription or post-transcriptional processes. Together, these data strongly suggest that both nMAT2 and PMH2 function in the splicing of many group II introns in Arabidopsis mitochondria.

### 2.3. nMAT2 and PMH2 Function in the Splicing of Similar Subsets of Group II Introns in Arabidopsis Mitochondria

To better understand the roles of nMAT2 and PMH2 in the processing of group II introns in plants, we compared the splicing efficiencies (i.e., the ratios of pre-RNAs to mRNAs) of the 23 group II introns found in the mitochondria of *Arabidopsis thaliana* plants [22], between wild-type (Col-0) plants and *nmat2* and *pmh2* mutants. Splicing defects were determined to be present in cases where the accumulation of a specific pre-mRNA was correlated with a reduced level of its corresponding mRNA in each mutant line. 

Although Northern blot and ribonuclease protection experiments indicated a role for nMAT2 in the splicing of *cox2* i1, *nad1* i2 and *nad7* i2, these analyses did not yield conclusive results with regard to the roles of nMAT2 in the processing of other transcripts in Arabidopsis mitochondria (Table 1 and [33]). To establish the roles of nMAT2 in the splicing of specific mitochondrial introns, we used transcriptome analyses by RT-qPCRs of total mtRNAs obtained from wild-type and mutant plants [34,35,40,60,64]. These analyses indicated to splicing defects in *cox2* i1, *nad1* i2, *nad1* i3, *nad2* introns 1 and 4, *nad4* i2, *nad5* introns 1,2 and 3, *nad7* i2, and the single intron within *rps3* (Figure 3). To some extent, reduced splicing efficiencies were also observed in the cases of *nad1* i4, *nad2* i3, *nad4* i3, *nad5* i4, *nad7* i4 and *rpl2* i1 (Figure 3). However, given the small degree to which their mRNAs levels were reduced in the mutant (Figure 2 and Figure 3), it is difficult to draw firm conclusions about the roles of nMAT2 in the splicing of these introns (Table 1). The RNA profiles further indicate that the splicing of *ccmFc* i1, *nad1* i4, *nad2* introns 2 and 3, *nad4* introns 1 and 3, *nad5* i4 and *nad7* introns 1, 3 and 4 did not rely upon nMAT2 (Figure 2 and Figure 3, and Table 1). The splicing of these introns is therefore expected to be facilitated by various other splicing cofactors.

Similar to the data shown in Köhler et al. [37], splicing defects in *pmh2* were supported in the cases of 15 mitochondrial introns (see Figure 3 and Table 1). These including *cox2* i1, *nad1* i2 and i3, *nad2* intron 1,2 and 4, *nad4* introns 2 and 3, *nad5* introns 1, 2 and 3, *nad7* introns 1 and 4, *rpl2* i1 and *rps3* i1, in which the accumulations of pre-RNAs were correlated with notable reductions in their corresponding mRNAs in *pmh2* mutants. However, we could not confidently draw any conclusions about the significance of PMH2 to the splicing of *ccmFc1* i1, *nad1* i4 and *nad7* i2, as although the steady-state levels of their pre-RNAs were higher in the mutant, the accumulation of their corresponding mRNAs (i.e., *ccmFc* mRNA, *nad1* exons d–e and *nad7* exons b–c) was not significantly affected in *pmh2* (Figure 2). Notably, the transcriptome profiles and splicing defects of *pmh2* mutants shared some similarities with those seen in *nmat2* mutants (Figure 2 and Figure 3, and Table 1). Introns that their splicing was affected in both *nmat2* and *pmh2* include *cox2* i1, *nad1* i2, *nad1* i3, *nad2* i1, *nad2* i4, *nad4* i2, *nad5* i1, *nad5* i2, *nad5* i3 and *rps3* i1 (Figure 2 and Figure 3, and Table 1).

Taken together, the RNA profiles (Figure 2 and Figure 3), and the data shown in Keren et al. [34] and Köhler et al. [37] strongly indicate that nMAT2 and PMH2 function specifically in group II introns splicing. In light of the expected significance of splicing to the functionality of many of the organellar transcripts, and hence to respiratory functions and plant physiology, we speculate that the lack of gross phenotypes associated with mutations in *nMAT2* or *PMH2* gene-loci may relate to redundant functions with other organellar splicing cofactors (i.e., maturases, RNA helicases, PPRs, PORRs or CRM-related proteins) that exist in Arabidopsis mitochondria [14,15,35,40]. Accordingly, the splicing of individual group II introns in plant mitochondria seems to rely on the activities of different protein cofactors [14,15,30]. While the functions of RNA helicases, including PMH2, are expected to affect a broad set of introns, the roles of the maturase-type nMat2 factor, which evolved from a protein acting only on the intron carrying its own gene, in the splicing of many group II introns in plant mitochondria are quite remarkable. Similarly, the organellar-encoded maturases, MatR and MatK, also act on multiple intron targets in the mitochondria and chloroplasts (respectively) of land-plants [60,65].

### 2.4. nMAT2 Is Found in Large Ribonucleoprotein Particles that Also Contain PMH2

**Figure 4:**
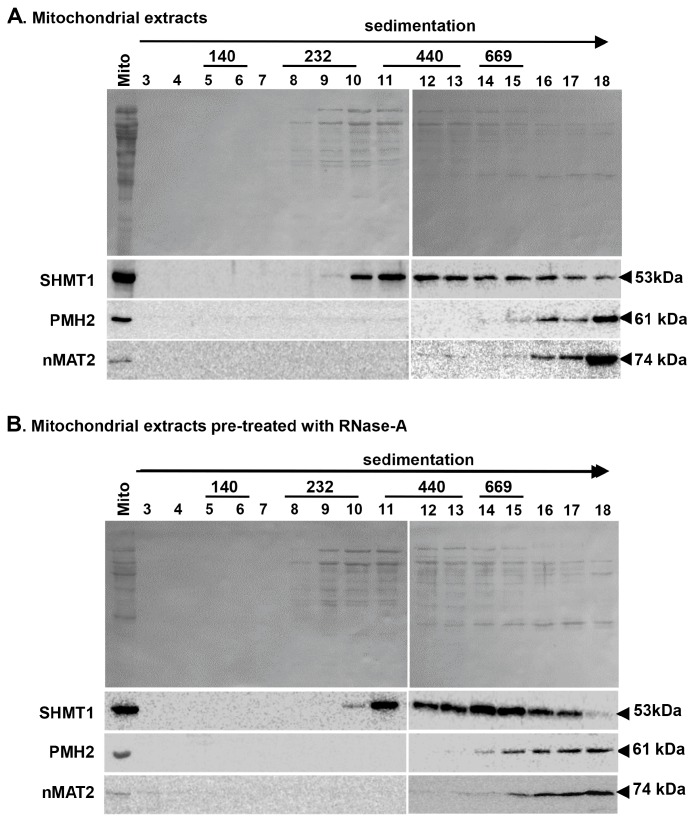
nMAT2 and PMH2 proteins are identified in large ribonucleoprotein particles in Arabidopsis mitochondria. Fractionation of wild-type and mutant mitochondria pre-treated with: RNase inhibitor (**A**); or ribonuclease A (RNase-A) (**B**) by sucrose gradient centrifugation. Aliquots of crude mitochondria protein extract (Mito) were solubilized in n-dodecyl-β-maltoside (DDM) (1.5% (*w*/*v*)), and sucrose gradient fractionated mitochondria samples (Fractions 3–18) were subjected to immunoblot analysis with antibodies raised against different organellar proteins (see “Materials and Methods”), including serine hydroxymethyltransferase 1 (SHMT1) protein (53 kDa), PMH2 (61 kDa, [36,37]) and nMAT2 (74 kDa, [33]), as indicated in each blot. High molecular mass standards (GE Healthcare) sizes are given in kilodaltons.

The experimental data indicate that nMAT2 and PMH2 are involved in the processing of similar subsets of group II introns in Arabidopsis mitochondria (Figure 2 and Figure 3, and Table 1). We thus intended to investigate whether these factors may cooperate in regulating the splicing of many mitochondrial pre-RNAs. Native organellar complexes were fractionated by velocity centrifugation sedimentation throughout sucrose gradients, and each fraction was analyzed by immunoblot analyses with antibodies against nMAT2 [33] and PMH2 [36,37]. Immunoblots with antibodies against serine hydroxymethyltransferase 1 (SHMT1) were used as a control. In agreement with previous analyses [33,36,37], both nMAT2 and PMH2 proteins migrated through the sucrose gradients towards the bottom of the tubes, with apparent molecular masses of ≥1000 kDa, which are significantly higher than their monomeric sizes (74 and 61 kDa, respectively) (Figure 4A). Interestingly, the signals of nMAT2 and PMH2 were observed in the same fractions, following the sucrose gradient centrifugation (i.e., fractions 16, 17 and strongly enriched in fraction 18). Notably, the particle sizes of nMAT2 and PMH2 proteins were reduced following the ribonuclease A treatment, as observed by their appearance in fractions of lower molecular weights (i.e., lanes 12–18) after the sucrose gradient fractionation (Figure 4B). Thus, the RNase-sensitivity assays lend additional support to the association of nMAT2 and PMH2 with organellar transcript in Arabidopsis mitochondria.

The similarities in the RNA profiles of *nmat2* and *pmh2* mutants and the appearance of both of these proteins in ribonucleoprotein (RNP) particles of similar molecular masses may indicate that nMAT2 and PMH2 cooperate in the splicing of group II introns in Arabidopsis mitochondria. Analogously, the splicing of group II introns in yeast mitochondria was also found to be mediated by both maturases and RNA helicases [52,53,66], although the specific roles of these factors in the splicing of group II introns are still under investigation.

Using a transgenic approach, we showed that the expression of a recombinant nMAT2 protein, which contains a hemagglutinin (3HA) tag at its carboxyl terminus, restores the splicing defects of *nad1* i2, *nad7* i1 and *cox2* i1 in *nmat2* mutants (Figure S4) [33]. Here, we further applied this method to identify proteins and RNAs that are stably associated with nMAT2, in vivo. Affinity purifications with anti-HA antibodies following mass spectrometry analyses, were used to identify proteins that co-purified with nMAT2-HA that were absent in co-IPs of Arabidopsis plants transformed with an empty vector control (Figure S4). Antibodies against SHMT1 were used as a control for the integrity of the co-immunoprecipitations (co-IPs) assays. Intriguingly, in addition to nMAT2, the co-IPs followed by LC-MS/MS analyses also revealed the presence of various peptides which corresponded to PMH2 protein, as well as two ribosomal factors (At1g16870 and At1g31817) and four pentatricopeptide repeat (PPR) proteins (i.e., At1g26460, At1g10270, At1g55890 and At1g61870) (Table S1). Other proteins that co-purified with nMAT2 included several prohibitins (At4g28510, At2g20530 and At5g44140), which are highly abundant in Arabidopsis mitochondria and are found in high molecular weight complexes [67]. It remains possible, therefore, that these may have nonspecifically co-purified with the nMAT2 particles.

Published data, as well as our own studies, indicate that organellar splicing factors in plants are tightly associated with their intron targets [33,60,65,68,69,70,71,72]. Here, we used the co-IPs to identify mtRNAs that are stably associated with nMAT2-PMH2 particles, in vivo (see “Materials and Methods”). The association of group II intron-containing pre-RNAs with nMAT2-PMH2 particles was supported in the cases of *cox2* i1, *nad1* i2, *nad1* i3, *nad2* introns 1, 2 and 4, *nad4* intron 3, *nad5* introns 1, 2 and 3, *nad7* introns 1, 2, 3 and 4 and *rps3* i1 (Table S2). The sequencing data also indicated the presence of *nad2* i2, *nad4* i3, *nad7* introns 1 and 4 and *rpl2* i1 in the co-IPs of *nmat2/nMAT2-HA* plants (Table S2). As the splicing of these introns is not affected in *nmat2* (Figure 3), we thus assume that these intron are likely associated with PMH2 protein. No cDNAs that correspond to *ccmFc* i1, *nad1* i4 and *nad4* i1 transcripts were detected in the pelleted RNAs. As the splicing of these introns does not depend upon nMAT2 or PMH2 (Table 1), these results strongly support the specificity of the co-IP analyses. 

### 2.5. Study of Double Mutants Affected in nMAT2 and PMH2 Gene-Loci

Mutations in nMAT2 and PMH2 affect the maturation of numerous pre-RNAs in Arabidopsis mitochondria, many of which are affected in both mutant lines (Figure 2 and Figure 3, Table 1). Fractionation of native organellar complexes and pulldown assays indicate that nMAT2 and PMH2 are found in RNP complexes of similar molecular weights, in vivo (Figure 4 and, Tables S1 and S2). We therefore anticipated that mutants homozygous to both gene-loci would show strong defects in the maturation of pre-RNAs that their splicing is assisted by both nMAT2 and PMH2 proteins (i.e., *cox2*, *nad1*, *nad2*, *nad4*, *nad5* and *rps3* pre-RNAs). For this purpose, the *nmat2* and *pmh2* plants were crossed, and the resulting double mutant-line, *nmat2/pmh2* (Figure S1B), was analyzed for its associated growth phenotypes and organellar activities. Phenotypic examination of *nmat2/pmh2* seedlings suggested that under optimal growth conditions i.e., in either long (16:8-h) or short day conditions (8:16-h), 22 °C, 100 μE m^−2^ s^−1^, primary root elongation, vegetative growth, flower morphology and fertility were only slightly affected in the double mutant, (Figure 1). However, we noticed that the leaves of *nmat2/pmh2* plants turn reddish when these are grown under higher light intensities (i.e., 300 μE m^−2^ s^−1^), suggesting the accumulation of anthocyanin pigments in the leaves (Figure 1D). Leaf redness through anthocyanin is often considered as a stress response (Chalker-Scott 1999). Accordingly, accumulation of anthocyanin and ROS was observed in other mutants affected in mitochondrial RNA metabolism, as *nmat1* mutants [34]. 

### 2.6. Double nmat2/pmh2 Mutants Are Strongly Affected in the Maturation of cox2, nad1, nad5 and rps3 Pre-RNA Transcripts in Arabidopsis Mitochondria

To identify changes in transcript levels in the double mutant line, we examined the splicing efficiencies of each of the 23 mitochondrial group II introns in wild-type and *nmat2/pmh2* plants by RT-qPCR analyses. Notable splicing defects were seen in *cox2* i1, *nad1* i2, *nad1* i3, *nad2* i1, *nad2* i4, *nad4* i2, *nad5* i1, *nad5* i2, *nad5* i3 and *rps3* i1. In many cases, the splicing in the double mutant line was more strongly affected than in the single *nmat2* or *pmh2* mutants (Figure 3), further supporting that the splicing of these introns depends upon the activities of both of these factors. Splicing defects were also apparent in the cases of *nad2* i2, *nad4* i3, *nad7* introns 1,2 and 4 and *rpl2* i1, which their splicing relies on either nMAT2 (*nad7* i2) or PMH2 (*nad2* i2, *nad4* i3, *nad7* i1, *nad7* i4 and *rpl2* i1). However, the splicing efficiencies of these introns in *nmat2/pmh2* were comparable with those seen in the single *nmat2* or *pmh2* mutant lines. To some extent, the levels of nad1 i4, nad2 i3 and nad7 i3 pre-RNAs were slightly higher in *nmat2/pmh2* plants compare to those of the wild-type plants (Figure 3). However, as their corresponding mRNAs levels were not significantly reduced in the mutant (Figure 2), it is difficult to draw firm conclusions to whether the splicing of these introns was affected in the double mutant. The splicing of introns that their splicing is not dependent upon nMAT2 or PMH2, including *ccmFc* i1, *nad1* i1, *nad4* i1 and *nad5* i4, was not significantly affected in *nmat2/pmh2* (Figure 3 and Table 1). The maturation of these RNAs is therefore facilitated by splicing cofactors other than nMAT2 or PMH2. Similarly to the single mutant lines, no significant difference in the relative accumulation of mRNAs corresponding to intron-less transcripts was observed between the wild-type plants and *nmat2/pmh2* mutant line (Figure 2). 

### 2.7. Analysis of Mitochondrial Respiratory Activity and the Biogenesis of Organellar Respiratory Chain Complexes in nmat2, pmh2 and nmat2/pmh2 Mutants

**Figure 5:**
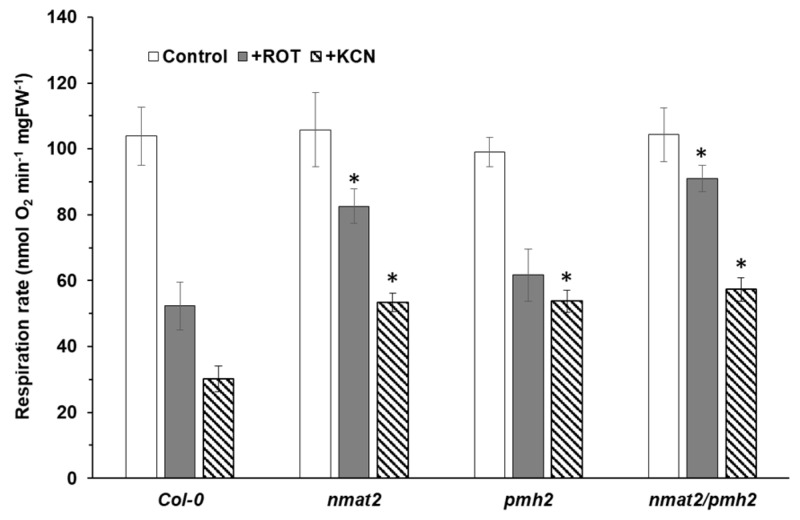
Respiration activities in wild-type and mutant lines. O_2_-uptake rates of wild-type plants and *nmat2*, *pmh2* and *nmat2/pmh2* mutants were analyzed with a Clark-type electrode as we described previously [35]. For each assay, equal weight (i.e., 100 μg) three-week-old MS-grown Arabidopsis seedlings were submerged in 2.5–3.0 mL sterilized water and applied to the electrode in a sealed glass chamber in the dark. O_2_-uptake rates were measured in the absence (Control) or in presence of rotenone (ROT, 50 μM) and potassium cyanide (KCN, 1 mM) which inhibit complexes I and IV activities, respectively. The values are means of four biological replicates (error bars indicate one standard deviation). The asterisk indicates a significant difference from wild-type plants (Student’s *t*-test, *p* 0.05).

The respiratory machinery is an aggregation of four major electron transport complexes (i.e., CI to CIV) and the ATP synthase enzyme (also denoted as CV), which function together in oxidative phosphorylation and drive the synthesis of ATP. Number of studies have shown that perturbation of splicing can affect mitochondria biogenesis and functions [14]. While the activity of complex IV is expected to be essential, complex I defects in plants result in a broad spectrum of phenotypes, ranging from mild to severe growth and developmental defects (reviewed by e.g., [73]). Interestingly, Arabidopsis mutants that are completely lacking complex I activity are strongly affected in their cellular physiology, but are viable when they are grown on sugar-containing MS media [74,75]. To analyze whether the respiratory activity was altered in *nmat2*, *pmh2* and *nmat2/pmh2* plants, the oxygen-uptake rates of wild-type and mutant-lines were monitored in the dark with a Clark-type electrode. When respiration was analyzed on three-week-old seedlings grown on MS-plates, the average O_2_-uptake rates of *nmat2* and *pmh2* mitochondria (105.76 ± 11.25 and 99.11 ± 4.45 nmol O_2_ min^−1^ gr FW^−1^, respectively) were similar to those measured in wild-type plants (103.84 ± 8.79 nmol O_2_ min^−1^ gr FW^−1^) (Figure 5). However, inhibition of mitochondrial respiratory chain complex I by rotenone (+ROT) affected the respiration rates of wild-type and *pmh2* plants (i.e., 52.32 ± 7.25 and 61.68 ± 7.95 nmol O_2_ min^−1^ gr FW^−1^, respectively), whereas the inhibitor appeared to has a less effect on the respiratory activity of *nmat2* plants (i.e., 82.54 ± 5.25 nmol O_2_ min^−1^ gr FW^−1^) (Figure 5). Similarly, while the average O_2_-uptake rates of *nmat2/pmh2* (104.29 ± 8.08 nmol O_2_ min^−1^ gr FW^−1^) were similar to those of wild-type plants, they were also less sensitive to inhibition by rotenone (91.06 ± 3.98 nmol O_2_ min^−1^ gr FW^−1^) (Figure 5). As the maturation of COX2 is affected in both the single and double mutant lines (Figure 2 and Figure 3), we also measured the O_2_-uptake rates in the presence of potassium cyanide (KCN), which inhibits electron transport through complex IV. The data shown in Figure 5 indicate that inhibition of mitochondrial respiration by KCN is more pronounced in wild-type plants than in each of the mutants. In summary, the respiration measurements suggest that complex IV is affected in *nmat2*, *pmh2* and the *double nmat2/pmh2* mutant-line, whereas complex I is more notably affected in the *nmat2* and *nmat2/pmh2* mutants. These results may correlate with the severity in the RNA metabolism defects observed in each of the mutants (Figure 2 and Figure 3). 

**Figure 6:**
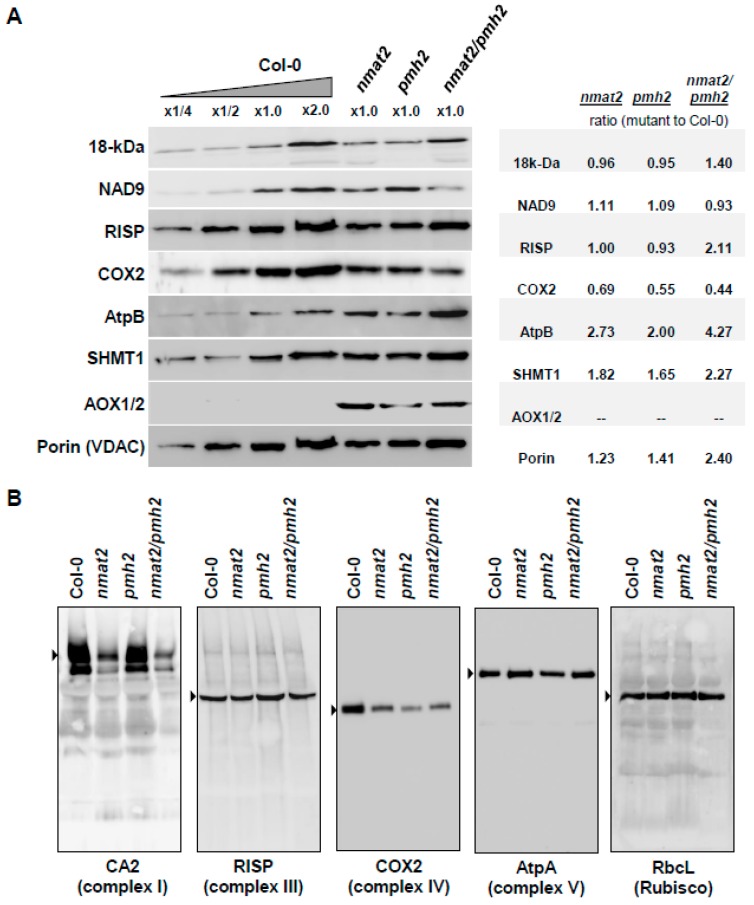
Relative accumulation of organellar proteins in wild-type plants and *nmat2*, *pmh2* and *nmat2/pmh2* mutants. (**A**) Immunoblots with total proteins (about 50 μg) extracted from three-week-old rosette leaves of wild-type plants, and homozygous *nmat2* and *pmh2* mutants. The blots were probed with polyclonal antibodies raised to γ-carbonic anhydrase-like subunit 2 (CA2), NADH-oxidoreductase subunit 9 (NAD9) and the 18-kDa subunits of complex I, Rieske iron-sulfur protein (RISP) of complex III, the cytochrome oxidase subunit 2 (COX2) of complex IV, mitochondrial ATP-synthase subunits and (AtpA and AtpB) of complex V, serine hydroxymethyltransferase 1 (SHMT1) protein and the mitochondrial voltage-dependent anion channel (VDAC, or Porin). Detection was carried out by chemiluminescence assays after incubation with HRP-conjugated secondary antibody; (**B**) BN-PAGE of crude mitochondria preparations was performed according to the method described previously [83]. Crude mitochondria preparations, obtained from three-weeks old Arabidopsis seedlings, were solubilized with DDM (1.5% (*w*/*v*)) and the organellar complexes were resolved by BN-PAGE. For immunodetections, the proteins were transferred from the native gels onto a PVDF membrane. The membranes were distained with ethanol before probing with specific antibodies (Table S3), as indicated below each blot. Arrows indicate to the native complexes I (~1000 kDa), III (dimer, ~500 kDa), IV (~220 kDa) and V (~600 kDa).

Protein accumulation depends on the balance between the rates of translation and protein degradation. Reduced mRNA transcript levels may also affect the organellar translation efficiencies. Accordingly, protein synthesis in the chloroplasts is correlated with transcript abundance [76]. The relative accumulation (i.e., steady-state levels) of different mitochondrial proteins in three-week-old *nmat2*, *pmh2* and *nmat2*/*pmh2* mutants was analyzed by immunoblot assays with antibodies raised against different organellar proteins (Table S3). These included the complex I subunits, 18-kDa (also termed as NDUFS4), NAD9, the Rieske iron-sulfur protein (RISP) of complex III, COX2 subunit of complex IV, the β subunit of the ATP synthase enzyme (AtpB, complex V), the plant mitochondrial voltage-dependent anion channel (VDAC, or Porin), the serine hydroxymethyltransferase 1 (SHMT1) protein, the mitochondrial alternative oxidase subunits 1 or 2 (AOX1/2) and the plastidial Rubisco enzyme (Figure 6). Relative protein levels were measured by densitometry of Western blots, and quantified using ImageJ software [77]. These analyses indicated that the abundances of the complex I 18-kDa subunit and Nad9 protein were similar in wild-type, *pmh2* and *nmat2* plants. The signals corresponding to the 18-kDa subunit and RISP protein seemed to be, at least to some extent, higher in the double mutant (1.40-fold and 2.11-fold, respectively; Figure 6A). However, the steady-state levels of COX2 subunit (Figure 6A), which its maturation was affected in *nmat2*, *pmh2* and the double mutant line (Figure 3 and Figure 4, and Table 1), were found to be somewhat decreased in the mutants (i.e., between 31% and 56% lower) than in the wild-type plants. Several other proteins, including the complex V subunit AtpB (2-fold to 4-fold), Porin (1.23-fold to 2.40-fold), SHMT1 (1.65-fold to 2.27-fold), and more notably AOX1/2, accumulated to higher levels in the mutants (Figure 6A). Upregulation in AOX expression is tightly associated with mitochondrial dysfunction and stress in plants [3,5,78,79,80,81,82]. 

Blue Native Polyacrylamide Gel Electrophoresis (BN-PAGE) was used to determine the effects of the mutations in nMAT2 and PMH2 on the biogenesis of the respiratory machinery. Separation of native organellar complexes by BN-PAGE and Western blot analyses revealed that complex I (using antibodies against the and the γ-type carbonic anhydrase CA2) was affected in *nmat2* and more notably in the mitochondria of *nmat2/pmh2* plants, while the levels of complex I in *pmh2* mutant were similar to those observed in the wild-type plants (Figure 6B). As the splicing of various *nad* transcripts is affected in both *nmat2* and *pmh2*, we speculate the differences in complex I levels between the mutant lines may relate to the strong maturation defects in *nad7* pre-RNAs seen in the *nmat2* mutant. In accordance with the splicing defects (Figure 2 and Figure 3) and reduced COX2 protein levels (Figure 6A), lower complex IV levels were evident in crude organellar preparations of *nmat2*, *pmh2* and the double mutant line. The respiratory chain complexes III and V were found in similar abundances in the mitochondria of wild-type and mutant plants (Figure 6B). Similarly, the accumulation of RuBisCO enzyme was also not significantly affected by the mutations in *nMAT2* or *PMH2* gene-loci. We, therefore, assume that the mtRNA maturation defects affect the biogenesis of the respiratory machinery in *nmat2*, *pmh2* and *nmat2/pmh2* plants. While the steady-state levels of COX2 and complex IV are reduced in the three mutant lines, the levels of the complex I 18-kDa and NAD9 subunits were not significantly affected in the mutant plants. Thus, reduced complex I levels, seen in *nmat2* and the double mutant line, may relate to altered translation rates or lower availability of different complex I subunits in these mutants.

## 3. Discussion

### 3.1. The Splicing of Group II Introns in Land-Plant Mitochondria Rely on the Activities of Different Nuclear-Encoded RNA-Binding Cofactors

The challenges of maintaining prokaryotic-type structures and functions within the cells are common to all eukaryotes. However, plants possess some of the most complex organelle compositions of all known eukaryotic cells. Plant mitochondrial genomes are unique in structural complexity, and gene-expression in plant mitochondria is highly complicated, involving multiple transcription initiation sites and extensive RNA processing steps [10,11,12,14,15]. These including the splicing of numerous group II-type introns that interrupt the coding regions of many essential genes. A major difference between bacterial group II introns and their counterparts in plant mitochondria resides in the nature of their target loci [21,57,84]. While in prokaryotes the group II introns often lie outside transcribed regions and are thus expected to have only a minor effect on bacterial fitness, the mitochondrial introns in plants reside within many genes required in both translation and respiratory-mediated functions [14,19,21]. These have diverged considerably from their bacterial ancestors, such as they lost many elements that are considered essential for splicing, and typically are also lacking their related maturase ORFs [19,20]. It is not surprising, therefore, that the splicing of the organellar introns in plants is accomplished largely by nuclear-encoded RNA-binding cofactors [14,15,60], which may also provide a means to link organellar functions with environmental and developmental signals. Here, we focus on the roles of the nuclear-encoded nMAT2 and PMH2 factors in the splicing of group II introns in Arabidopsis mitochondria.

### 3.2. nMAT2 and PMH2 Play Key Roles in the Splicing of Group II Introns in Plant Mitochondria

RNA-binding proteins play central roles in the post-transcriptional regulation of gene-expression in different biological systems. Accumulating data indicate that maturases and RNA helicases have pivotal roles in the splicing of group II introns (Reviewed by [14,15,21,57]). Model maturases encoded within group II introns function as intron-specific splicing cofactors, while RNA helicases belong to a large group of enzymes which carry multiple roles in gene-expression and RNA metabolism. To unravel the molecular basis of group II introns splicing processes in land-plant mitochondria, we utilized detailed expression analyses of wild-type, *nmat2*, *pmh2* and double *nmat2*/*pmh2* mutants. Both nMAT2 and PMH2 function in group II introns splicing (Figure 2 and Figure 3, and [33,37]). The mtRNA landscapes of *nmat2* and *pmh2* mutants indicated to perturbations in the maturation of many organellar pre-RNAs, all of which are containing group II intron sequences, while the steady-state levels of different intron-less transcripts were similar between wild-type plants and *nmat2* and *pmh2* mutants (Figure 2 and Figure 3). 

Significantly, the transcriptomes analyses revealed high similarities in the RNA profiles and splicing defects of *nmat2* and *pmh2* mutants. Introns that their splicing is notably affected in both *nmat2* and *pmh2* include *cox2* i1, *nad1* i2, *nad1* i3, *nad2* i1, *nad2* i4, *nad4* i2, *nad5* i1, *nad5* i2, *nad5* i3 and *rps3* i1 (Figure 2 and Figure 3, and Table 1). When the activity of both these factors is affected (i.e., in the double *nmat2*/*pmh2* mutant line), the maturation defects of these pre-RNAs are more noticeable in *nmat2*/*pmh2* then in each individual line (Figure 2 and Figure 3, and Table 1). Still, none of the analyzed processing events is completely abolished and mRNAs corresponding to the intron targets of nMAT2 and PMH2 are observed in the mutants (Figure 2 and Figure 3) [33,37]. We thus speculate that these results indicate to redundant functions with other splicing cofactors that exist in plant mitochondria [14,15]. Accordingly, MatR [60], nMAT1 and nMAT4 [33,34,35], the CRM-related mCSF1 protein [40,85,86] and the RNA helicase ABO6 [39] also influence the splicing efficiencies of multiple pre-RNAs in Arabidopsis mitochondria. For example, the splicing efficiencies of at least 13 different introns is affected in the *mcsf1* mutants [40]. The splicing of several of these introns, including *cox2* i1, *nad1* i2 and i3, *nad2* i1 and i4 and *nad5* i1, i2 and i3, also depends upon the activities of both nMAT2 and PMH2. Such a general substrate recognition is typical for RNA chaperones, which in many cases were shown to facilitate the transitions of the RNA ligands from non-functional, or intermediate structures, into the functionally-active forms (see e.g., [87]). 

### 3.3. nMAT2 and PMH2 Are Associated Together in Large RNP Complexes with Various Group II Introns in Arabidopsis Mitochondria

The RNA profiles indicate that nMAT2 is an atypical *trans*-acting maturase factor, which regulates the splicing of at least 11 out of the 23 group II introns in Arabidopsis mitochondria (Figure 2 and Figure 3, and Table 1) [33]. In accordance with previous reports [37], PMH2 was found to function in the splicing of 15 mitochondrial group II introns (Figure 2 and Figure 3, and Table 1). Interestingly, many of the introns that their splicing was affected in *nmat2* (beside *nad7* i2) are also identified as the intron targets of PMH2 (Table 1). The similarities in the RNA profiles of *nmat2* and *pmh2* mutants, and the fact that the splicing of these introns is more strongly affected in the double mutant-line (Figure 2 and Figure 3, and Table 1), may indicate that nMAT2 and PMH2 function together in the splicing of organellar pre-RNAs in Arabidopsis. Accordingly, separation of native mitochondrial preparations by sucrose gradients revealed that nMAT2 and PMH2 are both parts of high molecular weight ribonucleoprotein particles (Figure 4) [33,37]. Pulldown experiments further showed that PMH2 and several other proteins co-purify with the recombinant nMAT2-HA protein, in vivo (Table S1). The co-IPs also indicated to the presence of various group II introns (or pre-RNAs) in the nMAT2/PMH2-associated particles (Table S2). However, it remains unclear whether nMAT2 and PMH2 operate independently, or whether their functions in the splicing of mitochondrial introns may be coordinated. 

In an attempt to relate the experimental observations produced by the genetic and biochemical analyses with the overall tertiary structures of the mature forms of nMAT2 and PMH2 proteins (i.e., lacking their predicted N-termini targeting regions), we performed an atomic model of these proteins using the Phyre2 server [61]. The predicted structures of nMAT2 and PMH2 indicate the presence of positively charged surfaces that may serve as RNA-binding modules, while the uncharged or negatively charged regions may be required for protein–protein interactions (Figure S3). nMAT2 and PMH2 docking calculations were performed by Gramm-X [88], with no predetermined bias towards any specific residue interactions, and the predicted structures were visualized by PyMol [89]. The in silico analyses showed that PMH2 and nMAT2 may interact together throughout the association of amino acids found in a loop region between the two helicase domains of PMH2 (amino acids 327–363), and a short linker region (amino acids 461–485) between the fingers-palm and thumb domains of nMAT2 (Figure S5). However, this hypothetical model, which suggests an interaction between these two factors, needs to be supported experimentally. Nevertheless, the presence of nMAT2 and PMH2 together with group II introns in organellar RNPs (Figure 4 and, Tables S1 and S2) may relate to spliceosomal-like complexes in plant mitochondria [15]. Studies are under way in our laboratory to specify the binding characteristics of nMAT2 and PMH2 to group II intron RNAs and to examine whether these proteins associate with one another.

### 3.4. Mitochondrial Respiratory Activity Is Altered in nmat2 and pmh2 Mutants

Analysis of the protein profiles of wild-type and mutants plants indicated that complex I was affected in *nmat2* and *nmat2*/*pmh2* plants, while reduced levels of complex IV were apparent in the both single and double mutant-lines (Figure 6B). To examine if the reduced complexes I and IV levels affect respiratory functions, we measured the O_2_-uptake rates of Arabidopsis wild-type and mutant plants, using a Clark-type electrode. No significant differences in the O_2_-uptake rates were observed between the mutants and wild type plants. However, inhibition of mitochondrial respiratory chain complex I by rotenone had a stronger effect on wild-type and *pmh2* plants than on *nmat2* and *nmat2*/*pmh2* plants (Figure 5), which are affected in complex I biogenesis (Figure 6). We further analyzed the respiratory activities in the presence of KCN, a complex IV-specific inhibitor. As shown in Figure 5, the presence of cyanide had a stronger effect on wild-type plants (about 70% decrease), whereas the respiration rates in the mutant lines decreased by only about 50% in the presence of the inhibitor. These results are correlated with the splicing defects observed in the mutants (Figure 2 and Figure 3). Similar respiration rates seen in wild-type and mutant plants in the absence of inhibitors of the respiratory system (Figure 5), may correspond to the induction of alternative pathways of electron transport, via alternative oxidases (AOX) (Figure 6A) and/or type II NAD(P)H dehydrogenases (ND) [3,5,78,79,80,81,82]. Similarly, notable increases in the expression of various nucleus-encoded alternative oxidase genes, including AOXs and NDs, are seen in many other plants that are affected in mitochondrial RNA metabolism and the biogenesis of the respiratory system in plants (see, e.g., [34,35,40,43,50,64,74,90,91,92]. 

### 3.5. Morphology of nMAT2 and PMH2 Mutant Alleles

The results in Figure 1 and published data [33,36,37] indicate that the phenotypes of *nmat2* and *pmh2* mutants are comparable with those of wild-type plants. The single and the double mutants are all able to grow, flower and set viable seeds. In light of the expected significance of mitochondria functions to plant physiology, why does the loss of *nmat2* or *pmh2* cause only minor (or no obvious) phenotypic effects? Currently, we cannot provide a definitive explanation, but we speculate that the lack of strong phenotypes may be consequences of the multiplicity of splicing cofactors that are known to exist in plant mitochondria [14,15]. This assumption is supported by the fact that the mutations did not completely abolish the maturation of the pre-RNAs in *nmat2* and *pmh2* mutants, and the protein profiles and O_2_-uptake measurements which indicate that the respiratory activity is only partially affected in the mutants (Figure 5 and Figure 6). The splicing defects seem more noticeable in the double mutant line (Figure 2 and Figure 3), which also exhibit more notable growth and developmental defect phenotypes (i.e., shorter roots, altered leaf and flower morphologies, and leaf redness under higher light conditions) (Figure 1). However, despite the maturation defects, the corresponding mRNAs are still accumulating in the double mutant line. We therefore assume that in addition to nMAT2 and PMH2 other factors are likely involved in the splicing of each of the RNA targets. 

### 3.6. Maturases and RNA Helicases as Putative Proto-Spliceosomal Factors in Plant Mitochondria

As indicated above, maturases and RNA helicases serve as key factors in the splicing of group II introns in different organisms [14,15,21,57]. Canonical maturases encoded within group II introns bind with high affinities and specificities to their own host pre-RNAs, and thereby stabilize the catalytically active structure of the intron core [24,93], although in some rare cases these proteins may act on several closely related group II intron targets [94]. More notably are the functions of the organellar maturases in plants, which expanded their splicing functions to multiple intron targets (reviewed by [14,15,21]). Angiosperms contain six maturase-related proteins: MatK encoded by the *trnk* intron in the chloroplasts, the MatR ORF found within *nad1* i4 in the mitochondria, and four nuclear-encoded maturases (nMATs 1 to 4) that exist in the nucleus as self-standing ORFs, out of the context of their cognate introns, and are imported post-translationally into the mitochondria [14,15,31]. Both MatK and MatR function in the splicing of different organellar group II introns, and their functions seem essential in plants [60,65,95]. Likewise, the nuclear-encoded nMAT1 and nMAT4 also promote the splicing of different subsets of group II introns in the mitochondria [32,34,35,60]. Here, we show that nMAT2 acts as a more general splicing cofactor that influences the splicing of numerous introns in Arabidopsis mitochondria (Figure 2 and Figure 3, and Table 1). This evolutionary transition, where the intron-specific factors degenerated and evolved to regulate the splicing of multiple intron targets in plant mitochondria (all of which reside in protein-coding genes), may arose in plants as means to regulate organellar gene-expression in concert with cellular and environmental signals [15,21,57]. 

In addition to maturases, the splicing of organellar group II introns also depend upon the activities of other proteinaceous cofactors, which may assist in the folding of the large group II intron RNAs. In particular, DEAD-box RNA helicase seem to play central roles in introns splicing and RNA maturation (see, e.g., [36,37,52]). RNA helicases belong to a large group of enzymes that have multiple roles in RNA metabolism and gene-expression (reviewed by [96]). In yeast, *mss116p* mutants are defective in the splicing of the four mitochondrial group II introns, but are also seem to be affected in the splicing of group I introns and in the translation of various mRNAs in the mitochondria [52]. Our data show that, in Arabidopsis mitochondria, PMH2 is involved in the maturation of numerous group II introns-containing pre-RNAs (Figure 2 and Figure 3, and Table 1). However, based on the data in Köhler et al. [37] and our own results (Figure 2 and Figure 3), it is difficult to support any additional roles for PMH2 in mtRNA metabolism other than group II intron splicing. 

As with group II introns, the catalytic activities in the spliceosomes are also mediated by the RNA components, while the spliceosomal proteins, including the core pre-mRNA-processing 8 (PRP8) and different RNA helicases, are postulated to play roles in regulating RNA folding and conformational changes needed during the splicing reactions (reviewed by e.g., [97])**.** The assembly of the spliceosomes involves sequential rearrangements in protein-RNA and RNA-RNA interactions, which are driven by RNA helicases, such as the PRP5 and PRP28 proteins (see, e.g., [98]). PRP5 appears to mediate RNA-protein interactions during early pre-spliceosome formation (i.e., complex A). In a subsequent reaction, involving PRP28, the U4, U5 and U6 snRNPs are recruited for the formation of the pre-catalytic spliceosome (complex B) that catalyzes the first reaction leading to the release of exon 1 and the formation of an intron-exon 2 lariat intermediate. The second catalytic step is mediated by PRP8 and leads to mRNA formation and the release of the intron lariat. Intriguingly, the topology of PRP8 resembles that of maturases [99,100,101], which also facilitate RNA folding and ribozyme catalysis (see e.g., [15,21]). It was therefore hypothesized that PRP8 has evolved from a maturase-related RNA chaperon, and that during eukaryotic evolution has acquired additional domains and protein cofactors, such as RNA helicases, to facilitate spliceosome assembly and introns splicing [15,97]. The involvement of maturases and DEAD-box proteins in group II intron splicing in plants and yeast mitochondria may be homologous to the spliceosome, where PRP8 and various RNA helicases function at multiple steps to facilitate pre-RNA folding and intron splicing. 

## 4. Materials and Methods

### 4.1. Plant Material and Growth Conditions

*Arabidopsis thaliana* (ecotype *Columbia*) was used in all experiments. Wild-type (Col-0) and *nmat2* mutant lines (SALK-064659) were obtained from the Arabidopsis Biological Resource Center (ABRC) at Ohio State University (Columbus, OH, USA). Homozygous *pmh2* mutant line (SAIL-628C06) was generously donated from the Binder’s laboratory (Ulm University, Ulm, Germany). Prior to germination, seeds of wild-type and mutant lines were surface-sterilized with bleach (sodium hypochlorite) solution and sown on MS-agar plates containing 1% (*w*/*v*) sucrose. The plates were kept in the dark for 5 days at 4 °C and then grown under long day condition (LD, 16:8-h) in a controlled temperature and light growth chamber (Percival Scientific, Perry, IA, USA) at 22 °C and light intensity of 300 μE m^−2^ s^−1^. After two weeks, the germinated seedlings were transferred to soil and cultivated in the growth chamber, under similar growth conditions (i.e., 22 °C, 60% RH and light intensity of 300 μE m^−2^ s^−1^) in either short (SD 8:16-h) or long (LD 16:8-h) day conditions. PCR was used to screen the plant collection and check the insertion integrity of each individual line (specific oligonucleotides are listed in Table S4). Sequencing of specific PCR products was used to analyze the precise insertion site in the T-DNA lines.

### 4.2. Establishment of a Homozygous Double nmat2/pmh2 Mutant Line

Double *nmat2*/*pmh2* mutants were generated from single homozygous *nmat2* and *pmh2* lines, SALK-064659 and SAIL-628C06, respectively by genetic crossing. The resultant F1 seeds were sterilized, grown on kanamycin-containing MS medium and their seedlings were screened for double mutants (i.e., *nmat2/pmh2*). The self-fertilized F2 seedlings were further confirmed for the presence of *nmat2* and *pmh2* alleles by genomic PCR, and the double *nmat2/pmh2* mutant was then used for the analyses of its associated growth and developmental phenotypes and organellar activities.

### 4.3. Microscopic Analyses of Arabidopsis Wild-Type and Mutant Plants

For the analysis of plant morphology, whole plants and different organs obtained from wild-type and homozygous lines were examined under a Stereoscopic (dissecting) microscope.

### 4.4. Respiration Activity

Oxygen consumption (i.e., O_2_ uptake) measurements were performed with a Clarke-type oxygen electrode, and the data feed was collected by Oxygraph-Plus version 1.01 software (Hansatech Instruments, King’s Lynn, Norfolk, UK), as described previously [102]. The electrode was calibrated with oxygen-saturated water and by the addition of excess sodium dithionite for complete depletion of the oxygen in water housed in the electrode chamber. Equal weights (100 mg) of 2-week-old seedlings were immersed in water and incubated in the dark for a period of 30 min. Total respiration was measured at 25 °C in the dark following the addition of the seedlings to 2.5 mL of water. 

### 4.5. RNA Extraction and Analysis

RNA extraction and analysis was performed essentially as described previously [35,40,60,103]. In brief, RNA was prepared following standard TRIzol Reagent protocols (Thermo Fisher Scientific, Waltham, MA, USA) with additional phenol/chloroform extraction. The RNA was treated with DNase I (RNase-free) (Ambion, Thermo Fisher Scientific) prior to its use in the assays. RT-qPCR was performed with specific oligonucleotides designed to intron-exon regions (pre-mRNAs) and exon-exon (mRNAs) regions corresponding to the 23 intron-containing mitochondrial transcripts in (Table S5). Reverse transcription was carried out with the Superscript III reverse transcriptase (Invitrogen, Carlsbad, CA, USA), using 1–2 μg of total RNA and 100 ng of a mixture of random hexanucleotides (Promega) and incubated for 50 min at 50 °C. Reactions were stopped by 15 min incubation at 70 °C and the RT samples served directly for real-time PCR. Quantitative PCR (qPCR) reactions were run on a LightCycler 480 (Roche-Diagnostics, Basel, Switzerland), using 2.5 μL of LightCycler 480 SYBR Green I Master mix (Roche-Diagnostics, Basel, Switzerland) and 2.5 μM forward and reverse primers in a final volume of 5 µL. Reactions were performed in triplicate in the following conditions: pre-heating at 95 °C for 10 min, followed by 40 cycles of 10 s at 95 °C, 10 s at 58 °C and 10 s at 72 °C. The nucleus-encoded 18S rRNA (At3g41768) and the mitochondrial 26S ribosomal rRNA subunit (ArthMr001) were used as reference genes in the qPCR analyses.

### 4.6. Total Protein Extraction and Analysis

Protein analysis was performed essentially as described in [33,60]. Total protein was extracted from three-week-old Arabidopsis leaves or isolated mitochondria by the borate/ammonium acetate method [104]. For this purpose, frozen plant tissue was homogenized in the presence of polyvinylpolypyrrolidone (PVPP) (1:1 *w*/*w* ratio). The homogenate was added to microfuge tubes containing 400 μL ice-cold protein extraction buffer (50 mM Na-borate, 50 mM ascorbic acid, 1.25% (*w*/*v*) sodium dodecyl sulfate (SDS), 12.5 mM β-mercaptoethanol, pH 9.0) and the protease inhibitor cocktail “complete Mini” from Roche Diagnostics GmbH (Mannheim, Germany). Proteins were recovered by centrifugation (25,000× *g*) in the presence of three volumes of ice-cold 0.1 M ammonium acetate in methanol buffer (NH_4_-OAc-MeOH), generally as described in [104]. Protein concentration was determined according to the Bradford method (BioRad, Hercules, CA, USA), with bovine serum albumin used as a standard. Approximately 20 μg total protein was mixed with an equal volume of 3× protein sample buffer [105], supplemented with 50 mM β-mercaptoethanol, and subjected to 12% SDS-PAGE (at a constant 100 V). Following electrophoresis, the proteins were transferred to a PVDF membrane (BioRad; Hercules, CA, USA) and blotted overnight at 4 °C with specific primary antibodies. Detection was carried out by chemiluminescence assays after incubation with an appropriate horseradish peroxidase (HRP)-conjugated secondary antibody.

### 4.7. Preparation of Mitochondria from MS-Grown Arabidopsis Seedlings

Crude mitochondria extracts were prepared essentially as described in [83]. For the preparation of organellar extracts from *A. thaliana*, 200 mg of 14-days-old seedlings were harvested and homogenized in 2 mL of 75 mM MOPS-KOH, pH 7.6, 0.6 M sucrose, 4 mM EDTA, 0.2% polyvinylpyrrolidone-40, 8 mM L-cysteine, 0.2% bovine serum albumin and protease inhibitor cocktail “complete Mini” from Roche Diagnostics GmbH (Mannheim, Germany). The lysate was filtrated through one layer of miracloth and centrifuged at 1300× *g* for 4 min at 4 °C (to remove cell debris). The supernatant was then centrifuged at 22,000× *g* for 10 min at 4 °C. The resultant pellet, containing thylakoid and mitochondrial membranes, was washed twice with 1 mL of wash buffer 37.5 mM MOPS-KOH, 0.3 M sucrose and 2 mM EDTA, pH 7.6. Protein concentration was determined by the Bradford method (BioRad; Hercules, CA, USA) according to the manufacturer’s protocol, with bovine serum albumin (BSA) used as a calibrator. For immunoassays, crude mitochondria fractions were suspended in sample loading buffer [105] and subjected to SDS-PAGE (at a constant 100 V). Following electrophoresis, the proteins were transferred to a PVDF membrane (BioRad; Hercules, CA, USA) and incubated overnight (at 4 °C) with various primary antibodies (Table S4). Detection was carried out by chemiluminescence assay after incubation with an appropriate horseradish peroxidase (HRP)-conjugated secondary antibody.

### 4.8. Blue Native (BN) Electrophoresis for Isolation of Native Organellar Complexes

Blue native (BN)-PAGE of crude mitochondria fractions was performed generally according to the method described by Pineau et al. [83]. An aliquot equivalent to 40 mg of crude Arabidopsis mitochondria extracts, obtained from wild-type *nmat2*, *pmh2* and *nmat2/pmh2* plants (see above; Protein extraction and analysis) was solubilized with n-dodecyl-β-maltoside [DDM; 1.5% (*w*/*v*)] in ACA buffer (750 mM amino-caproic acid, 0.5 mM EDTA, and 50 mM Tris-HCl, pH 7.0), and then incubated on ice for 30 min. The samples were centrifuged 8 min at 20,000× *g* to pellet any insoluble and Serva Blue G (0.2% (*v*/*v*)) was added to the supernatant. The samples were then loaded onto a native 4 to 16% linear gradient gel. For “non-denaturing-PAGE” Western blotting, the gel was transferred to a PVDF membrane (BioRad; Hercules, CA, USA) in Cathode buffer (50 mM Tricine and 15 mM Bis-Tris-HCl, pH 7.0) for 16 h at 4 °C at constant current of 40 mA. The mitochondria were then incubated with antibodies against various organellar proteins and detection was carried out by chemiluminescence assay after incubation with an appropriate horseradish peroxidase (HRP)-conjugated secondary antibody. In-gel complex I activity assays were performed essentially as described previously [92].

### 4.9. Co-Immunoprecipitations

Mitochondria extracts from nmat2/nMAT2-HA plants were solubilized with 1% NP-40 (*v*/*v*) in assay buffer (150 mM NaCl, 10 mM sodium phosphate buffer, pH 7.2) at a protein concentration of about 1 mg/mL. After 30 min incubation on ice, the organellar extract was centrifuged for 10 min at 21,000× *g*. The clear supernatant was then incubated with anti-HA antibodies conjugated to protein A/G sepharose beads, with gentle mixing at 4 °C for 16 h. The beads were collected by a brief centrifugation (1 min at 1000× *g*, at 4 °C) and washed three times with 0.6 M NaCl, 0.5% (*v*/*v*) NP-40, 50 mM Tris-HCl (pH 8.3), followed by a single wash with 1× PBS buffer. The identity of the proteins in the co-IPs was established by LC-MS/MS analysis (The Smoler Proteomics Center, Technion, Haifa, Israel) (see also Table S1). Commercial antibodies to SHMT were used as controls for the integrity of the co-IP method. Total mtRNA that co-precipitated with the anti-HA antibodies was DNase digested and then reverse transcribed, using the Superscript III reverse transcriptase and a random hexanucleotide mixture. The identity of the co-purified mtRNAs was established by PCR of the cDNA library with specific oligonucleotides designed to organellar pre-RNAs (see Table S6) and sequencing (The Center for Genomic Technologies, The Hebrew University of Jerusalem, Israel). 

## Figures and Tables

**Table 1:** List of group II intron and their splicing efficiencies in *nmat2*, *pmh2* and double *nmat2*/*pmh2* mutants.

Mitochondrial Group II Introns	Introns Configuration	nMAT2-Dependent	PMH2-Dependent	Splicing Affected in *nmat2*/*pmh2*
*ccmFc* i1	cis	No ^a,c^	No ^b^, ambiguous ^c^	No
*cox2* i1	cis	Yes ^a,c^	Yes ^b,c^	Yes
*nad1* i1	*trans*	No ^a,c^	No ^b,c^	No
*nad1* i2	cis	Yes ^a,c^	Yes ^b,c^	Yes
*nad1* i3	*trans*	ambiguous ^a^, Yes ^c^	Yes ^b,c^	Yes
*nad1* i4	cis	No ^a,c^	No ^b,c^	ambiguous
*nad2* i1	cis	ambiguous ^a^, Yes ^c^	Yes ^b,c^	Yes
*nad2* i2	*trans*	No ^a,c^	Yes ^b,c^	Yes
*nad2* i3	cis	No ^a,c^	No ^b,c^	No
*nad2* i4	cis	ambiguous ^a^, Yes ^c^	Yes ^b,c^	Yes
*nad4* i1	cis	No ^a,c^	No ^b,c^	No
*nad4* i2	cis	ambiguous ^a^, Yes ^c^	Yes ^b,c^	Yes
*nad4* i3	cis	No ^a,c^	Yes ^b,c^	Yes
*nad5* i1	cis	ambiguous ^a^, Yes ^c^	Yes ^b,c^	Yes
*nad5* i2	*trans*	ND ^a^, Yes ^c^	Yes ^b,c^	Yes
*nad5* i3	*trans*	ND ^a^, Yes ^c^	Yes ^b,c^	Yes
*nad5* i4	cis	ambiguous ^a^, No ^c^	No ^b^, ambiguous ^c^	No
*nad7* i1	cis	No ^a,c^	Yes ^b,c^	Yes
*nad7* i2	cis	Yes ^a,c^	No ^b^, ambiguous ^c^	Yes
*nad7* i3	cis	ambiguous ^a^, No ^c^	No ^b,c^	ambiguous
*nad7* i4	cis	ambiguous ^a^, No ^c^	Yes ^b,c^	Yes
*rpl2* i1	cis	ambiguous ^a,c^	Yes ^b,c^	Yes
*rps3* i1	cis	ambiguous ^a^, Yes ^c^	Yes ^b,c^	Yes

^a^, splicing defects in *nmat2* mutants as indicated in Keren et al. [33]; ^b^, reduced splicing efficiencies in *pmh2* mutants as indicated by Köhler et al. [37]; ^c^, splicing defects supported by the transcriptome data and RT-qPCR analyses (this study); Grey shaded columns indicate to decreased splicing efficiencies in the double *nmat2/pmh2* mutants; ND, Not determined.

## Supplementary Materials

The following are available online at www.mdpi.com/1422-0067/18/11/2428/s1.
