# Supplementary material for: Analysis of the Roles of the Arabidopsis nMAT2 and PMH2 Proteins Provided with New Insights into the Regulation of Group II Intron Splicing in Land-Plant Mitochondria
Source: Int J Mol Sci. 2017 Nov 17;18(11):2428. doi: 10.3390/ijms18112428 (PMC5713396; doi:10.3390/ijms18112428)
Supplement: Supplementary file 1 [file Fig. S1-Gene structure_PCR screen.pdf › Fig. S1-Gene structure_PCR screen.pdf]

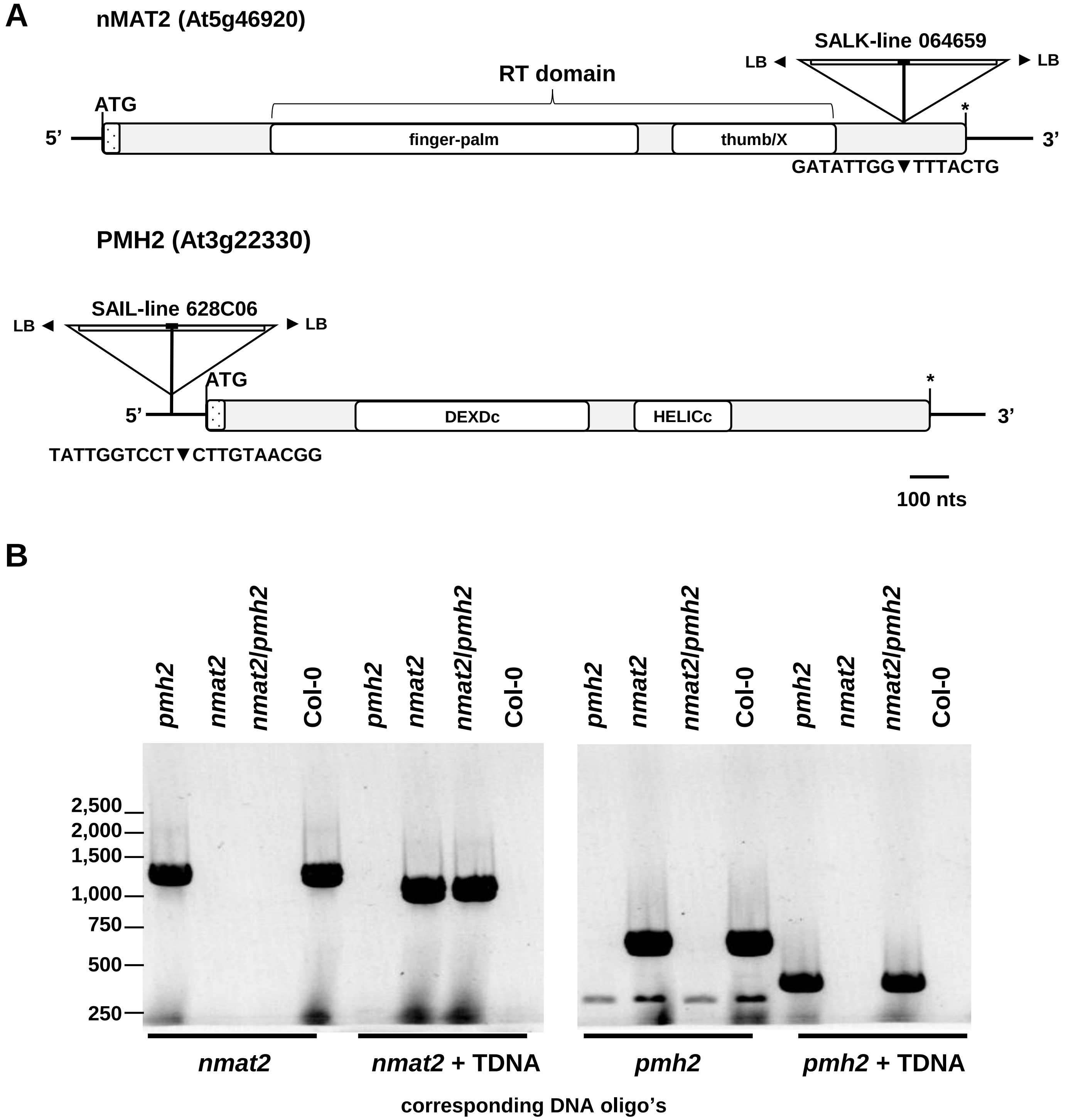

Figure S1. Schematic representation of the nMAT2 and PMH2. (A) Schematic representation of nMAT2 (At5g46920) and PMH2 (At3g22330). The different motifs are highlighted within the sequences of nMAT2 and PMH2. (B) The locations of the T-DNA insertion sites in *nmat2* (SALK-line 064659), *pmh2* (SAIL-line 628C06) and the double mutant line (*i.e*. *nmat2*/*pmh2*) target regions were verified by PCR, and are also outlined in the sequences of nMAT2 and PMH2. Primers are listed in Table S4.
