# Supplementary material for: Analysis of the Roles of the Arabidopsis nMAT2 and PMH2 Proteins Provided with New Insights into the Regulation of Group II Intron Splicing in Land-Plant Mitochondria
Source: Int J Mol Sci. 2017 Nov 17;18(11):2428. doi: 10.3390/ijms18112428 (PMC5713396; doi:10.3390/ijms18112428)
Supplement: Supplementary file 1 [file ijms-18-02428-s001.zip › Fig. S2 cDNA sequences.pdf]

A. *nmat2* (At5g46920) full cDNA sequence:

5'…agaagaggaagaagtcgaagctgttcatcaatcgtccgaaATGCGTAGAAGCTTCTCTGTTTTGGGTCCTTACAAATGGCTAAAACCTTCTTCTACATATACCAACGT
TTACTGCTTCGTCACAAATCCTCCTCTCATTTCTAACCCTAACAACGGCTCTGCGATTTTCCGATATCTATCTTACTTCGCACCTCAACAACGAAGACAACAACAACAAGC
ACCAGATCCAGATGATCCAGCGAATCTATTGAAAGAAGATGGAGTATCTCTCTGTTCTCAGATGTGGCTTGAGAATTTCAAAGAACCTGACAAAACCGCTACTAATTTAAC
CTCGTATCTTCGTAGATTCGAGTTATGGGTATTAGCTTATCAGAAAGTTTGCTGTGATGAATTAGGTGCTTATGTTCCTCGTAGCTCTATTCAAAGATCAGCTTTAGAGAA
TTTGTTAGCTTTAAGAAACTCTGTTCTCGATGATCGGTTTAAATGGGGTTCTCGGTTGGATTTCTATATTAAATCTCCTAGAGATAAGACCGATTACGAGTCGTTGTCGAA
GAGGAAGATTAAAGCGATTTTGACTACTACGCAGCCTACTCCGTTTCAGGATAGGATAGTTCAGGAGGTTTTGTTGATGATTTTGGAACCGATATACGAATCTCGGTTTTC
GCAAAAATCGTTTGCGTTTCGGCCCGGGAGGACCGCGCATACGGTGTTGAGAGTGATTCGGAGGAATTTCGCGGGTTATTTGTGGTATGTTAAGGGTGATTTGAGTGTTGT
GTTGGATGGAATGAAAGTTGGTTTTGTGATTAGTTCTTTGATGAGAGATGTTAGAGATAAGAAAGTGATTGATTTGATCAAATCTGCGCTTGTTACACCTGTTGTAACGAG
TAAAGTTGAAGATGGTGAGAAGAAGAAGACTAAGAAGAGAAAATATCAGAAAAAACGGGTTTTGGCTGAGGATGAGCCCAAGCCTGATCCTTATTGGTTAGAGACGTTCTT
TGGTTTTGCTCCTGAGGAAGCTGGGAAGTCTCCGCAGTGGGGACATTGCGGGATTCTTAGTCCTCTTTTGGTTAATGTATGTCTTGACGAGCTTGATCGTTGGATGGAAAC
GAAAGTGAAAGACTTTTACCGTCCTTCGAAAAGCGATGTTATATGGAATAACCCTGAAGGAGAAGCGGATCAAGGAAACACTTCTTGGCCGGAATTTGTTCCCACGAGTGG
TCCTGACAAAACGAGGAAAATGGATTACGTTAGATATGGAGGTCATATCTTGATTGGTGTCCGTGGACCTCGAGCTGATGCTGCAACGTTAAGGAAAGAGCTTATTGAGTT
TGTTGATCAGAAGTATATGCTTAGACTCGACAATGAGAATCTACCCATTGAACATATAACAAAAGGTATAATGTTTCTTGATCATGTTTTGTGTCGCCGAGTTGTTTATCC
GACTCTGAGGTATACTGCGACCGGTGGGAAGATTATAAGCGAAAAAGGCGTGGGAACGCTTTTATCCGTTACAGCGAGTTTGAAGCAATGCATCAAACAGTTTCGAAAGCT
GTTATTTATAAAAGGAGATCGTGATCCGGATCCACAGCCTTGTTTTAGAATGTTCCACGCAACTCAAGCTCATACGAATAATCAGATGAATAAGTTTTTGACTACGATTGC
TGAGTGGTATCGGTTTGCAGATAACAGAAAGAAAATTGTGAATTTCTGTTCTTATATTATCCGTGGTTCACTTGCTAAGCTTTATGCTGCAAAGTATAAACTTAGATCAAG
AGCAAAAGTGTATAAGTTCGCTAATCGGAATCTGAGCCTTCCGTTGTTGCAGAAGAAAGGTCAGTCACCAGAATATCAGAATCTCTTAAGAATGGGTCTTGCTGAATCGGT
TGATGGACTTGTTTATACAAGAATGTCTCTAGTTCCAGAAACTGATTACTCGCCTTTCCCGGGTAATTGGAGGCCCGAGCACGAGAAGTTTCTGATCGAGTACTTAACACT
CGATGAACCGAAAACCTTAGAAGAGCAAAAGAGATTTATTAGAGAGAAAGGGCTCGTTTCGCCTCAGGATTACACATCGATGTTGGTTTGGAACTATAAGAGAAATGCTAT
TCCAATGGATCAAGTTTCAATTCTAAAGGATCAACCTTTCTTGTTGGGATCATCAAGTACCTACAACCGCGACAATGATGATCAGAAGAATAAAGAAGAAGATGAAGATAG
TGAAGATGGGC▼TTCGCATTGCACGCATGTAAgaaaccagaaaactgtttgacttttactcttatatcaagcttgtataatttgatgccatattagccattccctatccgg
tttgtttcggctattgtatgcaaaacaccaaatttattagcattggtattttc…3'

nMAT2 (At5g46920) protein sequence:

Predicted targeting site

MRRSFSVLGPYKWLKPSSTYTNVYCFVTNPPLISNPNNGSAIFRYLSYFAPQQRRQQQQAPDPDDPANLLKEDGVSLCSQMWLENFKEPDKTATNLTSYLRRF**ELWVLAYQ**

KVCCDELGAYVPRSSIQRSALENLLALRNSVLDDRFKWGSRLDFYIKSPRDKTDYESLSKRKIKAILTTTQPTPFQDRIVQEVLLMILEPIYESRFSQKSFAFRPGRTAHT

VLRVIRRNFAGYLWYVKGDLSVVLDGMKVGFVISSLMRDVRDKKVIDLIKSALVTPVVTSKVEDGEKKKTKKRKYQKKRVLAEDEPKPDPYWLETFFGFAPEEAGKSPQWG

Fingers-palm region

HCGILSPLLVNVCLDELDRWMETKVKDFYRPSKSDVIWNNPEGEADQGNTSWPEFVPTSGPDKTRKMDYVRYGGHILIGVRGPRADAATLRKELIEFVDQKYMLRLDNENL

Thumb (X) domain

PIEHITKGIMFLDHVLCRRVVYPTLRYTATGGKIISEKGVG**TLLSVTASLKQCIKQFRKLLFIKGDRDPDPQPCFRMFHATQAHTNNQMNKFLTTIAEWYRFADNRKKIVN**

FCSYIIRGSLAKLYAAKYKLRSRAKVYKFANRNLSLPLLQKKGQSPEYQNLLRMGLAESVDGLVYTRMSLVPETDYSPFPGNWRPEHEKFLIEYLTLDEPKTLEEQKRFIR

EKGLVSPQDYTSMLVWNYKRNAIPMDQVSILKDQPFLLGSSSTYNRDNDDQKNKEEDEDSEDGLRIARM

B. *pmh2* (At3g22330; full cDNA sequence)

5'…ggaacta▼aaacccctttgccttctttcttcatctctctaaatccctaattctccaaattttggggttttacattttccttatcaagagaacacctgaatctgaaaATG
ATCACTACAGTGCTACGACGATCTCTCCTTGATGCGTCGAAGAGGAATCTCTCCGCTTCGTTGACCTCAATCAATACTGTTCTGTTTCACAACCTGGCCCCAGCCGCCACT
AGAGTCTCCGACCTAGCCTTAATTGGATCTTCCGATGTCAAAGCTGGGTTTCCGTTTGGTGTTGAAGCGAAAGGAATTCATTTTCAATCTGGGCCGTTGGATTTTAGAGCT
TCGATGGTTTCTCAAGCTGGATTTGCGATCTCTGAGAGTAGCGAGAGAAGGGTTGGTGATTCGGAGAGTGTTGGTGGTGATGGGCTTGCGATATCTGAGCTTGGGATATCT
CCGGAGATTGTTAAAGCTTTGAGTAGTAAGGGTATTGAGAAACTCTTTCCTATTCAGAAAGCTGTGCTGGAGCCAGCGATGGAGGGTCGAGATATGATTGGTCGTGCTAGG
ACTGGAACGGGAAAGACACTTGCTTTTGGGATTCCTATCATTGACAAAATCATCAAATACAACGCTAAACATGGACGAGGGAGGAATCCGCTTTGTTTGGTTTTGGCACCG
ACAAGAGAGCTTGCTCGCCAGGTTGAGAAGGAGTTTAGGGAGTCTGCTCCGAGCTTGGATACTATTTGTCTTTATGGAGGTACACCGATTGGACAGCAAATGAGGCAGCTT
GACTATGGTGTTGATGTGGCGGTTGGAACTCCAGGTCGTGTCATTGATCTGATGAAGAGAGGAGCTTTGAATCTATCGGAAGTTCAGTTTGTGGTTCTGGATGAAGCTGAT
CAGATGCTTCAAGTTGGATTTGCTGAGGATGTTGAAATAATATTGGAAAAGTTGCCTGAGAAACGTCAGAGTATGATGTTTTCTGCAACAATGCCGAGTTGGATCAGATCT
CTCACTAAGAAGTACCTGAATAATCCTTTGACAGTTGATCTTGTTGGAGATTCTGATCAGAAACTAGCAGATGGAATTACGACATATTCTATCATAGCAGATTCTTATGGA
AGAGCATCCATTATTGGTCCTCTTGTAACGGAGCATGCCAAAGGAGGAAAATGCATTGTTTTTACACAAACAAAACGAGATGCTGATCGCCTTTCATATGCATTGGCTAGA
AGCTTCAAATGTGAAGCTTTACACGGTGATATATCCCAGAGTCAGAGGGAAAGAACGCTTGCTGGTTTCCGAGATGGGCATTTCAATATTCTTGTTGCGACTGATGTTGCT
GCCCGTGGACTTGATGTACCTAATGTCGATTTGATCATTCACTATGAGCTTCCTAATAACACGGAGACATTTGTCCATCGAACCGGGCGAACGGGTCGGGCTGGAAAGAAA
GGAAGTGCTATTCTGATCTACAGTCAAGATCAATCCAGGGCTGTAAAAATAATTGAGAGAGAAGTTGGGAGCCGATTCACTGAGCTCCCTAGCATTGCTGTGGAACGTGGA
AGCGCAAGCATGTTTGAAGGAATAGGTTCTCGATCCGGTGGTTCCTTTGGAGGAGGCATGAGGGATCGTGGTTCAAGCTTTGGTGGTCGTTCAGGCGGTGGTGGTTATGGT
GGCAGCAGTGGTGGTTATGGTGGTGGGCGTTCAGGCGGATCAAGCAACCGTTATTCTGGTGATTCTGACCGTTCTGGTTTTGGGAGCTTCGGTATGCGTTCTCCTGAGGGA
TATGGTTCGGATCGTTCATCTCAGTCAGGTGGAAGGAGCAGCTTTGGTGGTGGTCGTTCAGGCGGATCATCAAACAATCGTTCCTCTGGTTTCGGGGACTTTGGCTCTGAC
CGTTCTTCTCAGTCAGGCGGAAGGAGCAGCTTTGGTGGGTTTGGATCAAATGATGGGAAAAGATCTTACTGAgagaagtgtttttggagccgagccggcaataattggttt
gaccctaggaggagggaggattcggtgactcgagtagattagttactacggcggatacgaaggagtcaagttatatatataaatctgtagctatgtaaaacaaacatatac
tttcgaagttatgttatcatcatttcatatgaggtgattatcttcttttgcataatttgtttgtttatatt…3'

PMH2 (At3g22330) full protein sequence

Predicted targeting site

MITTVLRRSLLDASKRNLSASLTSINTVLFHNLAPAATRVSDLALIGSSDVKAGFPFGVEAKGIHFQSGPLDFRASMVSQAGFAISESSERRVGDSESVGGDGLAISELGI

(ATP-binding site) DEAD-boxlike helicase domain

SPEIVKALSSKG**IEKLFPIQKAVLEPAMEGRDMIGRARTGTGKTLAFGIPIIDKIIKYNAKHGRGRNPLCLVLAPTRELARQVEKEFRESAPSLDTICLYGGTPIGQQMRQ**

LDYGVDVAVGTPGRVIDLMKRGALNLSEVQFVVLDEADQMLQVGFAEDVEIILEKLPEKRQSMMFSATMPSWIRSLTKKYLNNPLTVDLVGDSDQKLADGITTYSIIADSY

helicase c-terminal domain

GRASIIGPLVTEHAKGGKCIVFTQTKRDAD**RLSYALARSFKCEALHGDISQSQRERTLAGFRDGHFNILVATDVAARGLDVPNVDLIIHYELPNNTETFVHRTGRTGRAG**K

KGSAILIYSQDQSRAVKIIEREVGSRFTELPSIAVERGSASMFEGIGSRSGGSFGGGMRDRGSSFGGRSGGGGYGGSSGGYGGGRSGGSSNRYSGDSDRSGFGSFGMRSPE

GYGSDRSSQSGGRSSFGGGRSGGSSNNRSSGFGDFGSDRSSQSGGRSSFGGFGSNDGKRSY

Figure S2. The topology of nMAT2 and PMH2.

The cDNA sequences of nMAT2 (A) and PMH2 (B) were obtained by RT-PCR with specific oligonucleotides (underlined and marked in red letters). The PCR products were cloned into the pGEM-T vector system II (Promega) and sequenced (The Center for Genomic Technologies at the Hebrew University of Jerusalem). The resulting sequencing data is illustrated on the corresponding sequences of nMAT2 and PMH2. Capital blue letters indicate the coding regions, while black lower-case letters represent 5' and 3' UTR regions. The insertion sites of T-DNAs in *nmat2* (SALK-line 064659) and *pmh2* (SAIL-line 628C06) genes are indicated by red arrows. The deduced amino acid sequences of nMAT2 and PMH2 are given below each cDNA sequence. Arrows represent conserved domains in nMAT2 and PMH2 as predicted by the SMART (Letunic *et al.* 2012) and Conserved Domain Database (CDD) (Marchler-Bauer *et al.* 2003) servers. These include the N-termini mitochondrial targeting signals (marked in blue) and the fingers-palm (amino acids 104 - 461) and thumb (X) domains (amino acids 486 - 625) of nMAT2 and the two conserved motifs typical to DEAD-box RNA helicases (amino acids 124 - 326 and 364 - 443). The consensus ATP binding site (amino acids 148 - 154) is underlined.
