# Supplementary material for: Analysis of the Roles of the Arabidopsis nMAT2 and PMH2 Proteins Provided with New Insights into the Regulation of Group II Intron Splicing in Land-Plant Mitochondria
Source: Int J Mol Sci. 2017 Nov 17;18(11):2428. doi: 10.3390/ijms18112428 (PMC5713396; doi:10.3390/ijms18112428)
Supplement: Supplementary file 1 [file ijms-18-02428-s001.zip › Fig. S3-Prot. structures.pdf]

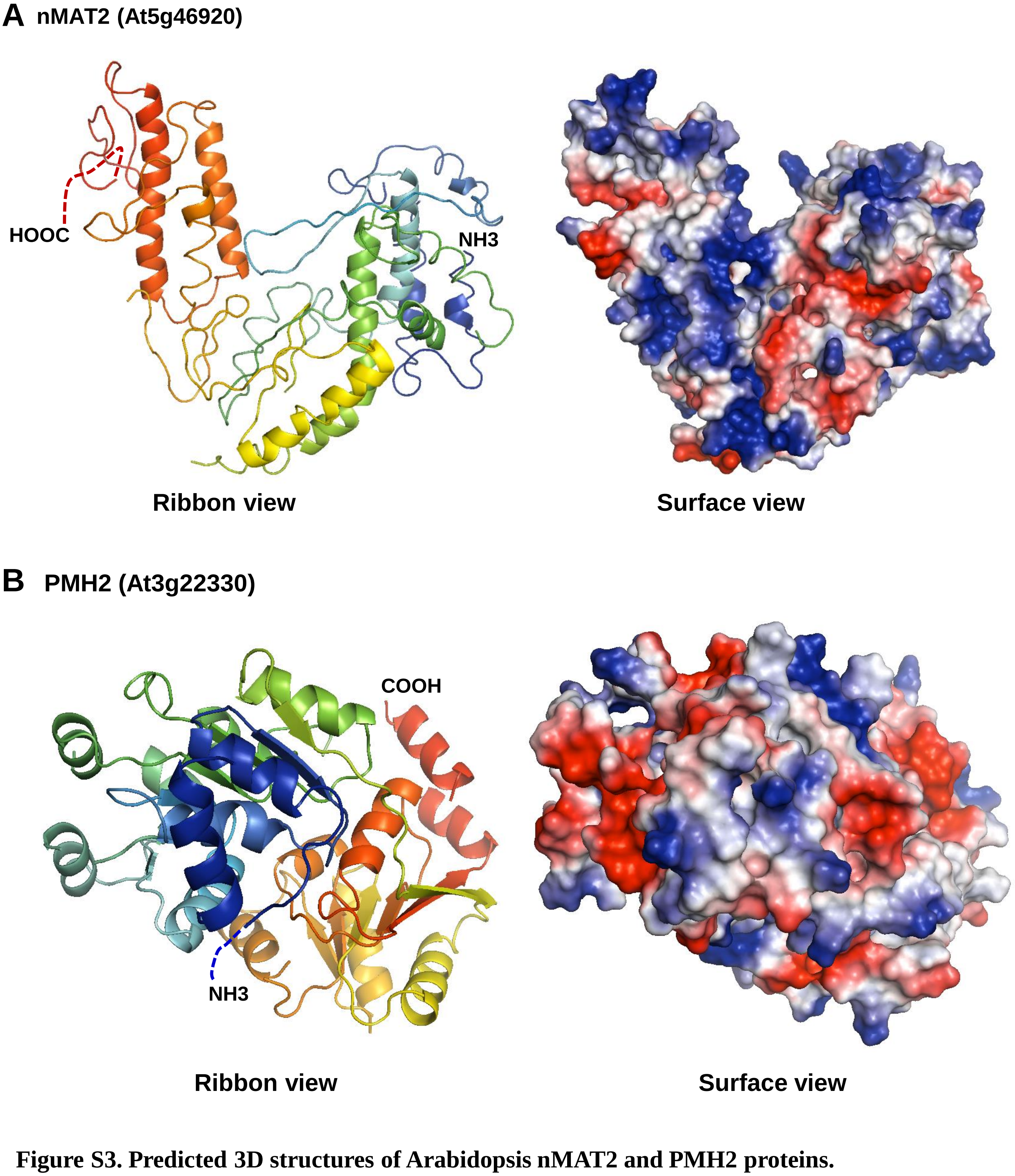

Figure S3. Predicted 3D structures of Arabidopsis nMAT2 and PMH2 proteins.
To get more insights into nMAT2 and PMH2 modes of action, in particular of RNA recognition, we performed an atomic 3D model of the Arabidopsis nMAT2 (A) and PMH2 (B) proteins, using the Phyre web server (Kelley and Sternberg, 2009). The hypothetical structures of nMAT2 and PMH2 proteins were visualized by the PyMol software (DeLano and Lam, 2005). The color code is red for negative values, white for near zero values, and blue for positive values. Positively charged surfaces are expected to be critical for RNA recognition and binding, while uncharged or negatively charged regions may function in protein-protein interactions.
