# Supplementary material for: Analysis of the Roles of the Arabidopsis nMAT2 and PMH2 Proteins Provided with New Insights into the Regulation of Group II Intron Splicing in Land-Plant Mitochondria
Source: Int J Mol Sci. 2017 Nov 17;18(11):2428. doi: 10.3390/ijms18112428 (PMC5713396; doi:10.3390/ijms18112428)
Supplement: Supplementary file 1 [file ijms-18-02428-s001.zip › Fig. S4. nmat2-HA comp.pdf]

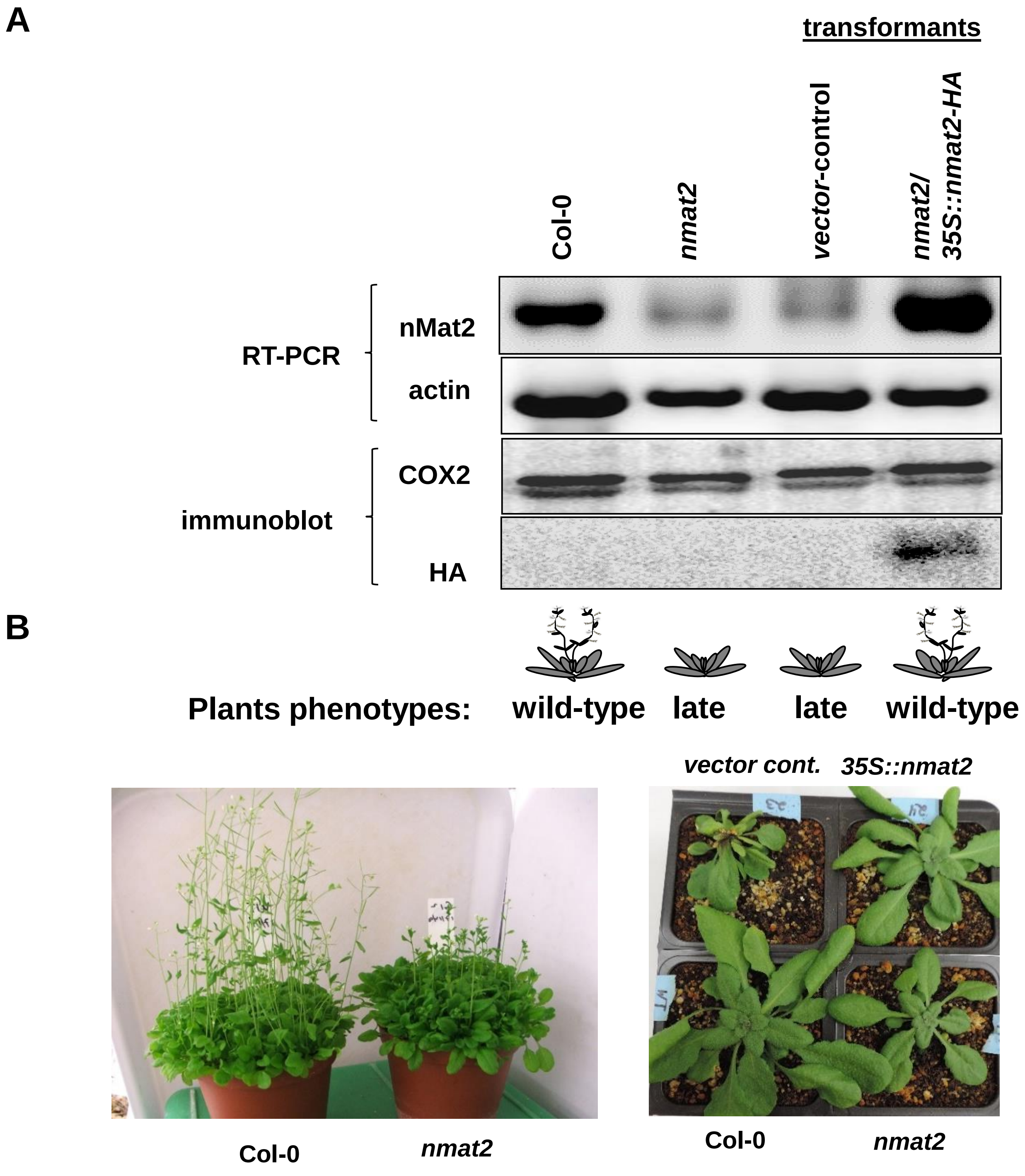

Figure S4. The expression of a recombinant nMAT2-HA protein in homozygous *nmat2* plants.
A. Gene expression was analyzed by RT-PCR with total RNA extracted from wild-type and mutant lines, and by immunoblots (modified from Keren *et al*. 2009) with total proteins (about 50 μg) extracted from 3 week-old rosette leaves of wild-type, homozygous *nmat2* plants and transgenic plant lines expressing an empty vector (control) or a recombinant nMAT2 protein which contains a hemagglutinin (3HA) tag at its carboxyl terminus (i.e. 35S::*nmat2-HA*). The blots were probed with polyclonal antibodies raised to nMAT2 (Keren *et al*. 2009), cytochrome oxidase subunit 2 (COX2), and commercial antibodies raised to the hemagglutinin (HA) tag. Detection was carried out by chemiluminescence assay after incubation with HRP-conjugated secondary antibody. B. Plant phenotypes associated with *nmat2* mutants and *nmat2* plants transformed with an empty vector control or a vector containing the nMAT2-HA under 35S promoter (*nmat2/35S::nmat2-HA*).
