# Supplementary material for: Analysis of the Roles of the Arabidopsis nMAT2 and PMH2 Proteins Provided with New Insights into the Regulation of Group II Intron Splicing in Land-Plant Mitochondria
Source: Int J Mol Sci. 2017 Nov 17;18(11):2428. doi: 10.3390/ijms18112428 (PMC5713396; doi:10.3390/ijms18112428)
Supplement: Supplementary file 1 [file ijms-18-02428-s001.zip › Fig. S5-docking model.pdf]

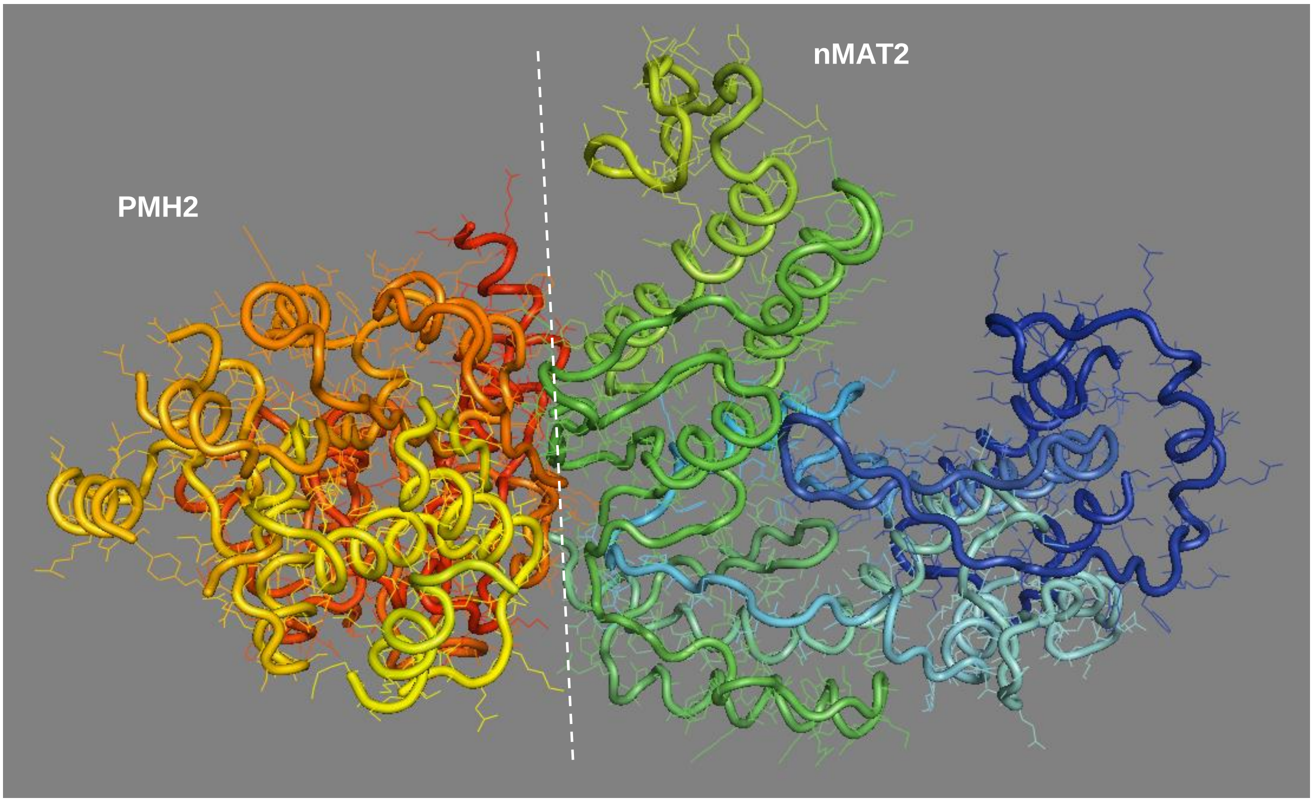

Figure S5. Predicted docking structure of Arabidopsis nMAT2 and PMH2 proteins.
A view of the model structure (see Figure S2) of PMH2 (yellow, orange and red) docked to nMAT2 protein (green, cyan and blue). Note that the model predicts that PMH2 binding to nMAT2 protein is mediated by amino acid residues found within a short linker regions between the two DEAD box domains in PMH2 and a linker region between the fingers-palm and thumb domains of nMAT2.
