# Supplementary material for: Analysis of the Roles of the Arabidopsis nMAT2 and PMH2 Proteins Provided with New Insights into the Regulation of Group II Intron Splicing in Land-Plant Mitochondria
Source: Int J Mol Sci. 2017 Nov 17;18(11):2428. doi: 10.3390/ijms18112428 (PMC5713396; doi:10.3390/ijms18112428)
Supplement: Supplementary file 1 [file ijms-18-02428-s001.zip › Table S1 - MS-MS results.pdf]

Table S1. Proteins identified in the co-IP assays by LC/MS-MS analysis

A. Proteins identified by co-IPs with the anti-HA antibodies from *nmat2*/*nMAT2-HA*

Peptides	At number	Notes
**Intron maturase, type II family protein (nMAT2)**<br>MYPYDVPDYAYPYDVPDYAYPYDVPDYA<br>SDVIWNNPEGEADQGNTSWPEFVPTSGPDK<br>IVQEVLLMILEPIYESR<br>YLSYFAPQQR<br>NSVLDDR<br>TAHTVLR<br>ADAATLR<br>GLVSPQDYTSMLVWNYK<br>EDGVSLCSQMWLENFK<br>FLTTIAEWYR<br>VEDGEK	**At5g46920**	**Maturase related protein / splicing**
**DEAD-box ATP-dependent RNA helicase 53 (PMH2/ARTH53)**<br>GLDVPNVDLIIHYELPNNTETFVHR<br>SGGGGYGGSSGGYGGGR<br>GRNPLcLVLAPTR<br>SSFGGFGSNDGKR<br>DADRLSYALAR<br>SGGSFGGGMR<br>AVLEPAMEGR<br>FTELPSIAVER<br>SSGFGDFGSDR<br>ESAPSLDTIcLYGGTPIGQQMR<br>TLAGFR<br>RSSFGGFGSNDGKRS<br>KTLAFGIPIIDKI	**At3g22330**	**RNA chaperon / splicing**
**pentatricopeptide repeat-containing protein**<br>INAYASLGNLQK<br>SALTVVDDMIK<br>VMDTALK<br>MLLLGK	**At1g26460**	**RNA metabolism?**
**pentatricopeptide repeat-containing protein**<br>HILANAPFAPSSVTYR<br>LPDSTSALVGQR<br>TMAFSSAEEAAAER<br>VVADFGAR	**At1g10270**	**RNA metabolism?**
**pentatricopeptide repeat-containing protein**<br>AGMFENAQK<br>IISLYGK<br>TNDFLK	**At1g55890**	**RNA metabolism?**
**pentatricopeptide repeat-containing protein (PPR36)**<br>IAFSAAVENLAEK<br>SEKDPDRILEIcR<br>AALSLLK	**At1g61870**	**Translation or RNA metabolism?**
**Mitochondrial 28S ribosomal protein S29-like protein**<br>STHAAVNVVVR<br>DTTLSQLTHK<br>WLAPFMR<br>KSYLALR<br>FDEAALK	**At1g16870**	**Translation**
**Probable ribosomal protein S11 (putative mitochondrial protein, SUBA3)**<br>FREQVPVSGESANNSGLSNTGR<br>MTNYTADATAENIGR<br>ATSGSLPDLK	**At1g31817**	**Translation**

AIIAFR		
**Prohibitin-1, mitochondrial (PHB1)**<br>VGIIGGLGLYGATHSLYNVEGGHR<br>SAQLIGQAIANNQAFITLR<br>AVVAQYNASQLITQR<br>DKVYPEGTHLMIPWFERPVIYDVR<br>ARPYLVESTSGSR<br>EIAQTIANSANK<br>VYPEGTHLMIPWFERPVIYDVR<br>VLPSIINETLK<br>SLGENYSER<br>EFTAAIEAK<br>QVAAQEAER	**At4g28510**	**Mitochondria biogenesis**
**Prohibitin-6, mitochondrial (PHB6)**<br>SAQLIGQAIANNQAFLTLR<br>AVVAQYNASQLITQR<br>VLPSIIHETLK<br>QVAAQEAER<br>AKPYLVESTSGSR<br>EIAQTISR<br>VLTRPMADQLPEVYR<br>SLGENYR<br>DKVYPEGTHLMIPWFERPIIYDVR	**At2g20530**	**Mitochondria biogenesis**
**Prohibitin-7, mitochondrial (PHB7)**<br>SAQLIGQAIANNEAFITLR<br>VLPSIINETLK<br>QVAAQEAER<br>VLTRPMGDR	**At5g44140**	**Mitochondria biogenesis**

B. Proteins identified by co-IPs with the anti-HA antibodies from *nmat2*/*empty vector control*

Peptides	At number	Notes
**Chaperonin CPN60**<br>KANLQDLAALTGGEVITDELGMNLEK<br>KANLQDLAALTGGEVITDELGmNLEK<br>QVANATNDVAGDGTTcATVLTR<br>LLEQDNPDLGYDAAKGEYVDMVK<br>TPVYTIASNAGVEGAVIVGK<br>LLEQDNPDLGYDAAKGEYVDmVK<br>AAVEEGILPGGGVALLYAAR<br>SAIELSTSDYDKEK<br>VTVSKDDTVILDGAGDKK<br>TQKcELDDPLILIHEK<br>MISTSEEIAQVGTISANGER<br>IGGASEAEVGEKKDR<br>cELDDPLILIHEK<br>TLFNELEVVEGmKLDR<br>RGISMAVDAVVTNLK	**At3g23990**	**Molecular chaperon / mitochondrial import and macromolecular assembly**
**Heat shock 70 kDa protein 10 (mtHsc70-2)**<br>ELLLLDVTPLSLGIETLGGVFTR<br>IELSSTSQTEINLPFITADASGAK<br>GVNPDEAVAMGAALQGGILR<br>AKIELSSTSQTEINLPFITADASGAK<br>IINEPTAAALSYGMTNK<br>SRFETLVNHLIER<br>SQVFSTAADNQTQVGIR<br>EKIPSEIAKEIEDAVADLR	**At3g23990**	**Chaperone / protein folding**

EAELHAQKDKER<br>GVNPDEAVAmGAALQGGILR<br>QAVTNPTNTVSGTKR<br>SSGGLSEDDIQK<br>EVDEVLLVGGMTR		
**Glycine dehydrogenase (decarboxylating) 2 (GLDP2)**<br>DKATSNIcTAQALLANMTAMYAVYHGPEGLK<br>VVMATDLLALTMLKPPGEFGADIVVGSGQR<br>VVMATDLLALTmLKPPGEFGADIVVGSGQR<br>RHNSATPDEQAQmANYcGFDNLNTLIDSTVPK<br>VVmATDLLALTmLKPPGEFGADIVVGSGQR<br>ESPYLTHPIFNMYHTEHELLR<br>FSGIFDEGLTESQmIEHMSDLASK<br>NImENPAWYTQYTPYQAEISQGR<br>RLMDYGFHGPTMSWPVPGTLMIEPTESESK<br>NIMENPAWYTQYTPYQAEISQGR<br>FGVPMGYGGPHAAFLATSQEYKR	**At2g26080**	**Glycine decarboxylase enzyme**
**ATP synthase subunit alpha (AtpA)**<br>EVAAFAQFGSDLDAATQALLNR<br>GmALNLENENVGIVVFGGDTAIKEGDLVKR<br>GMALNLENENVGIVVFGGDTAIKEGDLVKR<br>GMALNLENENVGIVVFGGDTAIKEGDLVK<br>AILNSVKPELLQALK<br>LTEVLKQPQYAPLPIEK<br>VVDAMGVPIDGKGALSDHEQR<br>GMALNLENENVGIVVFGGDTAIK<br>ATSESETMYcVYVAIGQK<br>GmALNLENENVGIVVFGGDTAIK<br>DNGMHALIIYDDLSK<br>DNGmHALIIYDDLSK<br>IRNFYANFQVDEIGR<br>GIRPAINVGLSVSR<br>KmELDAFLKER<br>KSVHEPMQTGLK<br>KSVHEPmQTGLK<br>KMELDAFLKER<br>EAFPGDVFYLHSR<br>NFYANFQVDEIGR	**AtMg01190**	**Complex V / membrane ATP synthase subunit alpha**

C. Proteins identified by co-IPs with the anti-SHMT antibodies from *nmat2/nMAT2-HA*

**Peptides**	**At number**	**Notes**
**Serine hydroxymethyltransferase 1 (SHMT1)**<br>AVmLADmAHISGLVAANVIPSPFDYADVVTTTTHK<br>WGVNVQPLSGSPANFHVYTALLKPHER<br>AVMLADMAHISGLVAANVIPSPFDYADVVTTTTHK<br>LDPEKWGVNVQPLSGSPANFHVYTALLKPHER<br>EVLYDFEDKINQAVFPGLQGGPHNHTITGLAVALK<br>ALEAFRLDPEKWGVNVQPLSGSPANFHVYTALLKPHER<br>VLEAVHIASNKNTVPGDVSAMVPGGIR<br>LKDFVSAMESSSTIQSEIAK<br>LKDFVSAmESSSTIQSEIAK<br>QLNAPLEEVDPEIADIIEHEK<br>ImALDLPHGGHLSHGYQTDTKK<br>KISAVSIFFETMPYRLDESTGYIDYDQMEK<br>GYELVSGGTDNHLVLVNLKPK<br>GLELIPSENFTSVSVMQAVGSVMTNK<br>INQAVFPGLQGGPHNHTITGLAVALK<br>GLELIPSENFTSVSVMQAVGSVmTNK	**At4g37930**	**L-serine metabolic process**

IMALDLPHGGHLSHGYQTDTKK<br>DFVSAMESSSTIQSEIAK<br>IMALDLPHGGHLSHGYQTDTK<br>ImALDLPHGGHLSHGYQTDTK<br>GLELIPSENFTSVSVmQAVGSVmTNK<br>YYGGNEYIDMAETLcQKR<br>LDESTGYIDYDQmEK<br>KISAVSIFFETMPYR<br>YYGGNEYIDMAETLcQK<br>QATTSEYKAYQEQVLSNSAK<br>YYGGNEYIDmAETLcQK<br>YYGGNEYIDmAETLcQKR<br>NTVPGDVSAMVPGGIR<br>LRHEVEEFAK<br>ISAVSIFFETmPYR<br>NTVPGDVSAmVPGGIR<br>LDESTGYIDYDQMEK<br>VAEYFDKAVTIALK<br>ISAVSIFFETMPYR<br>KISAVSIFFETmPYR<br>ALEAFRLDPEK<br>VLEAVHIASNK<br>ISAVSIFFETMPYRLDESTGYIDYDQmEK<br>LIVAGASAYAR<br>AYQEQVLSNSAK<br>HEVEEFAK<br>SATLFRPK<br>GFVEEDFAK<br>mGTPALTSR<br>FAQTLMER<br>YSEGYPGAR<br>MGTPALTSR<br>FAQTLmER<br>QATTSEYK<br>EVLYDFEDK<br>QFPTIGFEK<br>VAEYFDK<br>GAMIFFRK<br>AVTIALK<br>LYDYAR<br>ALEAFR		
**Serine hydroxymethyltransferase 2 (SHMT2)**<br>ImALDLPHGGHLSHGYQTDTKK<br>QLNASLDEIDPEVADIIELEK<br>INQAVFPGLQGGPHNHTITGLAVALK<br>IMALDLPHGGHLSHGYQTDTKK<br>IMALDLPHGGHLSHGYQTDTK<br>ImALDLPHGGHLSHGYQTDTK<br>YYGGNEYIDMAETLcQKR<br>KISAVSIFFETMPYR<br>YYGGNEYIDMAETLcQK<br>YYGGNEYIDmAETLcQK<br>YYGGNEYIDmAETLcQKR<br>LDENTGYIDYDQLEK<br>NTVPGDVSAMVPGGIR<br>ISAVSIFFETmPYR<br>NTVPGDVSAmVPGGIR<br>ISAVSIFFETMPYR<br>GYDLVSGGTDNHLVLVNLK<br>KISAVSIFFETmPYR<br>LIVAGASAYAR	**At5g26780**	**L-serine metabolic process**

LREMVEEYAK<br>VLELVHIAANK<br>mGTPALTSR<br>YSEGYPGAR<br>VAEYFDLAVK<br>MGTPALTSR<br>ALEAFQLDPSK<br>GFIEEDFAK<br>EVMYDYEDR<br>QFPTIGFEK<br>GAMIFFRK<br>LQSEMSK<br>AYQDQVLR<br>SAVLFRPK<br>LYDYAR<br>GAmIFFR			
