# Supplementary material for: Analysis of the Roles of the Arabidopsis nMAT2 and PMH2 Proteins Provided with New Insights into the Regulation of Group II Intron Splicing in Land-Plant Mitochondria
Source: Int J Mol Sci. 2017 Nov 17;18(11):2428. doi: 10.3390/ijms18112428 (PMC5713396; doi:10.3390/ijms18112428)
Supplement: Supplementary file 1 [file ijms-18-02428-s001.zip › Table S2 - co-IPs sequences.pdf]

cox2

ATGATTGTTCTAAAATGGTTATTtttTACAATTTCTCCTTGTGATGCAGCGGAACCATGGCAATTAGGATtTCAAGACGCAGCTACACCTATAATGCAAGGAATAATAGACTTACATCACGATATCTTTTTCTTCCTtATTCTGA
TTTTGGTTTTCGTATTATGGATCTTGGTTCGCGCTTTATGGCATTTCCACTATAAAAAAAATGtAATCCCGCAAAGGATTGTTCATGGAACTACTATCGAGATTCTTtGGACCATtTTTCCTAGTATCATCTtGATGTTCATTGC
TATACCATCATTTGCTCTCTTATACTCAATGGACGAGGTAGTAGTAGATCCAGCCATTACTATCAAAGCTATTGGACATCAATGGTATtGGACTTATGAGTATTCTGACTATAACAGTTCCGATGAGCAGTCACTCACTTTTGAC
AGTTATATGATTCCAGAAGAAGATCTAGAATTGGGTCAATtACGTTTATTAGAAGTGGACAATAGAGTGGTTGTACCAGCCAAAACTCATCTACGTATTATTGTAACATCTGCTGATGTACTTCATAGTTGGGCTGTACCTTCCT
tAGGTGTCAAATGTGATGCTGTACCTGGTCGTTTAAATCAAATCTCTATTTTGGTACAACGAGAAGGAGTTTACTATGGTCAGTGCAGTGAGATTTGTGGAACTAATCATGCCTTTAtGcgtgcgcccggaaacataggccgact
gttgagccccactctggctcagccgcaccaccccggggtgcgagccacccgagaagcaagctattacagtgagcggctggagctgtagggagccgaggagcaaggtagtagataagatagaagaaaagggccaggcacccggtgga
gcagaggggacggttaggataacgaacttgaaacgcggagcccgagcggctggcgagcgagtggttagtggccaatagcgccctagttgatggcattcctctctgcggctggcactcgaggaaccacagggcactccatacagag
caagcaagtcttagggatgagacgcccgcgcaaggacctcaattctcattaggaggtcgaaccaaggacctatggaagtcggggctaccccgtccccatgggcaatagtgtcctgagggaggagtttagaggccttatagtagca
tggacttcttgatctttctaggtcatgcgaagggggccagtccaagatcgtactgttcctctacaaagataatagacgctctcaacggctaggcgccactctctttctgagttattccagcttcttcatgatttcgatttcgtgc
cgcggtgaacaaacaaaaaaagagggccgtctcagcggaaggagaaggacctgcaacggcagagactactgaccctcttcttgttcttagccgtctattacgagtccgggaagcctggaatcataaatatgagggatccggaagg
gtgggcagggcttccgcaaggtttttttatcccctcttcccgggatgggaaaggagtcctatcaagtaaaggccataaccagcctctttttttatgtacacctttctttgtttcaggctcttcaagaccttcctcctgctaatc
cggccattaccgaacctgtctttcccacccttctcaattcttgcgattcctagccagccccttagcttattttgctttataaaaccactttctcctttttttcagcttgctgctcgctttctcgcttctgagaggtactgacgtg
actcgactgaaaggagaggcgaaaggagcctgacttacggtttcaaagcctggcgcgaagcgaagggattggatttcacctatgatcagattagtgggcaaccattgtttactttttttcgtggtgttgttgacgttgttgaaagc
taattaggaagtaggccttgcttttctccgctgggagcagctcacactatggtggaggaggtgccgtgaagatctaggagtgtgagcagtacgagctgaaaggctcccatactgtttggagggcagggggcatagatgccaaaga
aagctgacccctatCTATCGTCGTAGAAGCTGTTtCTAGGAAAGATTATGGTTCTtGGGTATCCAATCAATTAATCCCCCAAACCGGGGAAGCTTAA

***ccmFc* (*ccb452*)**

ATGGTCCAACTACATAACTTTTTCTTTTTTATTATTTTTATGGTCGTGCtTTGTGGCACGGCAGCACCCGTACTATTGAAATGGTTCGTCAGTAGAGATGTTtCCACGGGTGCCCCTTTTTtCAATGGTACTATAATTCCTATTC
tTATCTCTTtATTCtCTCTTTTGGTCTATtTACATTCAAGGAAAATCATACGCTCCATGGACGGAGCAAAAAGTGGAGTCTTGGTCAGAGCAAGTCGCCCTATTCTATTACCAGACATAATTGGGAGAAGCTCATCTGAAACTAG
AGCTAGAAAGGCCTTATTTTTTTTCGTTCCCGTTCTTCATTTCtGTCTTCTCGAATCCAAGGGGGACTTCTCATATTTAGAATCTTTCTGCGGTGTGCTCTGTTTACTATTCTTTtGTACTTTCtTCTTTTTAGCACGCGATAGG
TCAGCGAAGCGTGAGCGGGCGCGGAGAAGGAAAGGCCAAACACTTCGGCCGAACGGGAATGAGCAACGACGAAATGACAAGATGAGGTGCTCCGGGCACCCCCATTTAGATTTAGAAAGAAGGGTCGAAGGTTTTGGGCCTCTAG
CTTTCCCCGTCCCCCCTGAGTTGGGTGGTGCTTGTGTGGGGGGCGTGCCACCAGAAATCGGGCTTGAAGCTCTCGCCTTACCAAGGAGCCGACAGCTGATGGCTATGGCTGTTGGTCACGACTACTACCAAAAGGTTCCAATGAA
GATGAATATTTCACATGGAGGAGTGTGCATCTGTATGTTGGGTGTTCTTCTGTCGtgcgacccggcggcttatgtgcgacctgtggcccacgcctcctatttgttcagggcgggcggcgtgaactctgattcgatccgggtattc
aatcccgccgctgagatgctcagttgactccttaaccttgataggaagatggcttattccaaaattcgtgcataagggtaaggaactttggatgaactaatgcgaatgggtgtaagcttcgctgctcggaaacacccagtgctga
ccacactgagagacacgaaagcgcgggtaacgctagttggcgaagtggcgttaagcatccctagcggtacgaaaagagaggtcgtgatgatatcatctacgtccgtaccgctcctcgtggagtagatcccgcatccaaccaagtc
tttgaccagggaacgggataattcccactaccgctggcaggccagccgggccatgagcgcggtgggaacgggcttcccaaaaagccagccccgggccgggcggggtcagcatagaatgaaggggacggccctaatgttgtgttgg
caaagccaacttcttaggttgcggacggagaagagcggacgtggggactcgggtcggggtgcagcgtaactaagagagccattccatttagggcgagacagaatgggcgggcacgagcggtctggtgtccgagtcaattggtcag
acgacgactacttcactttattagattagtattagccgcctatcccggaatgaaatgagatagataaaatagaaagaacgcgaagcgctagcgctataggatttttttgggggggataagctcgcttcttcacaagcttacccccg
ccccgaccggcagctgctgggtctccccatctctcctaaacttcccccggtcttcggcccgagctgtatgaggcagaaactcgtcccacgtacggttcggaggccgagccccaccccagcagtaatggtgcggcttaggtcaact
AACACAAAGAAGATACAGTTCACTCAACGATTGCCTTTGGGTTACGAACTCCATATGGGGAAGGAACGTTGTTGTTTGCGAGGTCTCGATCATTTACATGGACCCACTTTTCATTCCATTTGTGGGAATTTGATGATCTATAAAt
CGTCCCTAACGAACGATCGGCTCATGTTTGAGCATGATGAATCACTTCATGCCGACCTCTTGCTAATAAACTTTCCGGCCTCATATAAGAATGGAAAACTTGAGCATTTTCTGCATTGGTGGATGAAGAATCGCAAACATAATAA
TTTTTGGTTAACCATGTTCCCAGAAAAAAGATACTTTCGAGAAAGGACGAGCACGGCTGAAGTGGCTATACATACAAATCTATTTACGGATCTATATGCTTtGATTGGAACTGGAAGTTCCAGAACAGGAGGTTGGTATACCAtC
ATAATGAAACTGCCTTTTATTTTTTTTTATTtGGATAGGATTTATGTTGGCTTCGTTGGGAGGCTtGCCTAGTTTGTTACGTCAGCTCCAAAAGGATAAGTTGCGTTGGAATtGAGAAAGTTCCGTGGAGTTCATAATTGCATAA

nad1

atgtacatagctgttccagctgaaatacttggaataattctaccacttctactaggagtagcctttttagtgctagctgaacgtaaagtaatggcttttgtgcaacgtagaaagggtcctgatgtagtgggatcgttcggattgt
tacaacctctagcagatggtttgaaattgattctaaaagaacctatttcaccaagtagtgctaattttttcctttttagaatggctccagtggctacatttatgttaagtctggtcgcttgggccgttgtaccttttgattatgg
tatggtattgtcagatttgaacatagggctactttatttgtttgccatatcttcgctaggtgtttatggaattattatagcgggttggtctagtaattaggggggcggccgttcggtcgcctatgagactaggaccaataggtcaa
aaattggtttgtgccgcaggtgttgaacgatctactctacacaggtgtgggcttacagggctagggctcagaaaccctttctttcattcatcaagagggctagacgcgcctttcgaaccagtctttataaacctggtcgctcact
tttccgggccgggatcaataagtagaagtcataaaaagatatgtttctcttcgcaccactgatagactactaaagattcaatttgaaaggccatacttagtttcgcaagcctttgtcattgtcagtcaactaagtaggtcgttcc
gcccccctgcgaatccgtaaattgaggagcatgccgcaacaaaaggatggtcccctatgcatttcattctttctagaaaggaaaattataagtacccccatcatggtgaacctctccttgtgatcgggatgaggtaaatgcctcct
tttcgggggggcggatcgaatcgcagtttccttaggtagccaccgacctacagttatccttaaacttccgtgcttggtggagaagaagcgaacaaaggtacgctcgcttgctgtcttgttctctgccgcgaactgggatcgctcgc
cagctaggtcagattgaagcaacatttttttgagaacatattaccccatcttcggggacaaggggcggaacgacctctcgatctacttacagcagcccaggaataaaaacgtcgtctaggcgttccctcttgctccgatctaccct
acgcctagga...aagcaaggatgccagtaccaaattttttcctcgggatgcatagccgccactatatcgtccaattccagtgcctgtaagagatcgggtttaccgactagtgtctttgattgatccaagtccccccgacagttca
atagcgcttccgcttcaggcctcgtaacctgttcataatgatcccgcataggtatcttactttcttagacaaaagcgggatagtgaaatagacgtccagtctccgatcccagttgattgatctcgactttaccagctcatgaacg
gggcattcatcagacttgagcagtaggtttcgactcggtcagctgtcttgatgaagattgacttcagccaaagagaatgaattgacttagggaaatattcctatgtgctcgatccgatcaacgaaaagaaatcctgaaattacat
aaagaaacgcgagaatcttcacagttgacattttcgattgagaaagtggcaggccaaaagagctctactgtagtgccttgcaaggtcgtagagccggtaaccctcgttcacctaaaatggaaaaaggtctgtgattgagactaag
gaacgctagctatatgcttaacacatgcaagtcgatcagttcgccctttcgcctatcggcttggcaggcaagctaccttgcttgcattctataaacggtaacttccgcgcccttcctgcctgcttaaattgatgtgaattgaatg

atggtgacagctcttcaaatcctattcagtctgattagatatgtcactgaaacaatccgttctgtctcagttttattttcggattccgaggatgagccggacgacgaagcctcctcctcagataaagatgtctcagacgctactc
tcccggcaagaacaacttattctattgtgatttttttggcgggttcggtcaggagagacaaaagagagatgctttctatcagtcaaaaggggactttgtctcccgccatgctcccacggcccgcttaccgaggaatagaaaggga
ggaccgggggggggtccagagcaagtagggttggggtatagagccgtaagcgcggtggggtgtgagagaggacgtgctcgtacggttcatagaagggtatgatcaactcgttgattgttggtaacttgaactcctctatatTCGA
AATATGCCTTTCTAGGAGCATTACGATCTGCAGCTCAAATGGTCTCTTATGAAGTATCTATTGGTCTTATTCTTATutgtgcgccttgtgagcgcgtttgtatccgcgaaggcaatcatcgctcggatgttcccctaacccaacc
cgggaacggaccggagggaaccgcagcatgggtaatgtccgcgtctcgtcgcaaggctcattttgagttgtgggtcataggtcggcttcggcgggcagctttatctgatcaaggccggggcacacgggtcctggtactatcc
aggtgcgaagaaccccggaggtgactgcaatgagcagaaatctcactcaccggcctaaacgacgagaaaacactcgaacgtgagagcaagggatcacccaacgaatggacgagctccaaggagggaggaggcaagcaccatgctt
tcagagaagtggcggtccgaatcttatctgaactgcgagaataactgactaagctgtgccataagggtaattctaaaagcgggacagggccaagcctttaggttgtttaagtaggttgggtgacagatcggccataggagtactc
cgggatataaaccagggcaacaaatgtcgagcatacaacgatgccgcccgttttcatttcatggaagtccccggcagaggaaagggctgtaggtgatggcgcgttttgcttcttatctagagaggggcgctgaaatcgttcctat
tgggtcgtgtgtggctttagtatagatgaataaaggcgggcgccgaaaaggaagcagcccagcctcttcaagaaagctttgcttggtaggcgctgcctactcactcggacaatgctctgaacacgagagtgtgcagttccgcccc
cttctctcatgctgagtcacaggcagcgcctcggaaagcacggacgagccacattgcagggaaacttgcacgtgtggttctggccggggacacccccggtatTACTGTACTAATATGTGTAGGTtCttGTAATTtGAGTGAGATT
GTCATGGCGCAAAAGCAGATATGGTtTGGTATTCCCCTGTTCCCTGTATTGGTTATGTTCtTTATTTCTtGTCTAGCAGAAACTAATCGAGCTCCGTTTGATCTCCCAGAAGCGGAAGCTGAATtAGTTGCAGGCTATAATGTAG
AATgcgcgggatgcgatccttaatagtccactgttggcggaagccaatgtcccggggtcccggggactcattctgactgaaactaggggtgggtctttaccaactcaaaaatattcgatttttgggaagacaaaaaacgtgatcg
cctagcgcgagtcttattgaaaaggccccttactatagatgccccttattgaaaactcaaggaaagcttgatatatatgtattatattaatgcgtaatgcgctcaagcatgcgaagaaagccggccttacctttagcaacaaggg
cgcttaggctttactaagaagtgcgaaagggcttttcttgcttgtttagtaaagtcacgctttttttatttagaattgagtaggtgcttgctggggcagggcactcttcattcttcattcgaaactttgaatgtcgggtcagagg
tttctgccttgttatcaaaaaggaagttgttgggtaaagcaaactccctccttggacgagcacggggcttagtcaagtagaaccgggttgcgctgcttgatgctccgatcgaaaacaaatcagtggggccatgccggtacgactg
aatatgcgttagaaaggtcaatccctcccctacgacaccaaaaaaaccgggccgaacttctaacagcccgcccgcttccatagaaaaatatcgcggcaacgtagacctaagtggtcatattggatccttgggaaccatcacaagt
acggccggcctatatttgaacgagaatgccgtgtcgttcagcggagcctaaaagaaggtgactcgcgcagacagctgactccttttcaatagaaaggaatagccaaaccaacccagctggctgatcaatctcagagatcttatcg
gccggcaaaccggagacggacgaccacggttccgaccttaccagcaccagaggtgtactaatcaatgaacccccgaaaccaactttattttctgacaaaatccaccaagtctaagcggtcacgacatct...ccctagtgaggaa
tcctttagaactgatgacgtcctagctgtgagctgacccaagagctacactgattaggaagcagattccgcacagaagaccaagcaaatctcctcttctagtcttacccccaggctgtggtgctacgagatggcaatttatcgta
tagaagaatagatccgtgggcctattgattgaccatagatcatagaaccgatcgactgctatctaagagaagataagtagttcttctatcgttcaagggcaaggggagaacatgtagcggcttgcctagatctcgtatttcccac
gtagtccgtaggtcaaaccaaggattctcttcacagtcttaaaaggatgtatctctcctttgcgctgacttatgctctgcgagctaggagcgcatcataggggcagggcgaaaacgaacataggatcatcatcatggcccttttc
ttctttcttttgctgctgatctcacatcgagagcagctccttcttgttcagggtcatcagatgagagatcttcttccgactccgagaaatcggtcaaacgggaggctaccctctccttttagtcgagtaatcaatccctcgactc
atctctctatccataaaaaagcccttcccagaggagagcggaagctccaggatgagtatgccctggattcccggattcattctcgtcctgatccactctggaattttcggaatctacaaaaacattctaggaaagggctcgtaat
gatcttctcaaagatcacaaggtgctgaagtggcaggataggaggggggatccgtagtttttgtgaaagggatgctcttacttatcatgtctgaaaagaaaaaccgaattcctcgatttcattcgattcatccacatttccatttc
ttgtagaggaaggctaactgtgcttgctggctgggagctgtatgagcggtaacgtccacgtacggttccgtgagaagggcggtggacagaaatggccttgttgtaccttactctcgATTCTTCAATGGGGTtTGCTCTTTTTTTT
TtGGGAGAGTATGCCAATATGATCTTAATGTGCGGAACTTTGCATCTGACATTCGTTGGGCTTCCCTCTTCGGGAGCCTGCGTCCCGGCCGTTTTTGTGCAATAAACCCCTCCGGCCGAAGACTAGTGGTAGGTGGTCCCGCGTA
GCTTTCGGAGAAGGGTAGCCTTGTGTGTAAGCACAGCAATGAACCGCGGCGAACCCTCAGACGACCTATCTAAGATTAGGGGGGGGGGAGATCCTCAGTAGTGGTGACCCTTTCACCTCTTCCCACGGACTGATACATGTACCGA
ATGCTCATACGGGAAAGTTGACTCCTGGGTCTGGAACCTGGGGGGTTGCTCCGAGAAATCCTTTCTTTCTCGTCCACTCAGGGGGGTGCGGACACACCCGCGCGGATTACAGGTGACGGTTACAAGAATGCGGGGAAGTGAACAG
TACCCGACGACATTCAGGGATGGATGTAGACCCATCGGGCAGGGATAATCATTCCGGTCCTGGGAGAGGTATTGATTACTCGACTAAAAGGAGAGGAACCCCATCGACTGAAAGAAGAGGAGAGGTGGCGACCATTTTCAAGAAC
CAAAAAGACTGAACTGAGGGAAGCCCTATGAGTCACTGAAACGACGGCAGGAGGGGGCCTTTGATTATAAAAAAGGGGAGCAAAAAACGAGCTTTTCCCCCCTTTACAATATGA{**matR_ORF**}tagtcgtttttttctattagt
tgcgatgcgaacaggcgtttacttatgagattagttgagtagacttgcctgagttgtctgctataagatagagctagttttggggtagggcttttgacataaaaaagccggataggcttggcttcgctatcgctcatgacttgta
ttgtagtcggcccggaatgcctcggtagtctttctaatgccttcttccttcattcattttcttttagttgcggtagcttccgcgccagcaagatacggacagcgaagccaaagcaatactaaacaagcgagaaaagtccttgtta
ttagtaaagcgctaacgatcaagaaaaggccccttactactagataggctaacacgcctttactaattatatatatatataataaggtatttctcaaagtaaagtttctagcttgtttctttagaaagattgcggggggcgctcacg
tttttggccccttcccggcccggaagttcgcttccggcgactagcttttacgctatcgcttggacttgtcacttcgtaccttgaatcaatcaatgaatgaatgaaaacgcctgactaggaatagaaaggaaggacaggttggtt
cgaggaccccttggtcaaaggaaaggtacaaaggaactcgaccacttgttgggagaggttgtgaaacaaactcgactaaaaggagaggtataaaaatgattccggggcggagccgtatgacgcgagagtgtcacgtacggtttct
ttgagaagggtgtgataccaccacctatcaggcccgacgagcggtccacggagctgcatccctactcaccGAGTGGTCtATGCACATTGTTCTTTCtAGGAGGTTGGCtGCCTATCCTAGATCTTCCCATTTTCAAGAAGATCCC
GGGCTCGATCTGGTTTAGTATCAAGGTTCTTtTCTTTCTGTTCCTATATATATGGGTtCGTGCAGCATTTCCACGATATCGTTATGATCAATTAATGGGACTTGGCtGGAAAGTGTTCTTGCCTCTATCATTAGCTtGGGTAGTC
tCTGTTTCTGGTCTTTTAGTCACCTTTCAATGGCTTCCTTAA

nad2

ATGAAAGCAGAATTCGTTCGGATCCTCCCACACATGTTCAATCTTTTTTTAGCGGTTTtCCCAGAGATCTTTATCATTAATGCAACCTttATTTTGCTCATTCATGGAGTTGTATTTAGTACCTCTAAGAAATATGATTATCCGC
CGTTAGtCAGTAATGTGGGTTGGCTTGGATTACTTAGTGTtgcgcgcctaggagggcagcgcgcttggggatgcagaggaactattatcgcccagccccctaccctaacctaatgcggcgccggattcggaccgcaggaaccgta
gcatgggggggggacgtctaatccttttgccgccgcaaggctggctaatcgtacgcagcaggctcgaagacccccctggttctggaaggcacatgagtccgaacgctatgttgaatgtgcgaccgacactacgtaggtaccagtg
caggtgaggcgtcggtcggtcctagaaacggcggcaacggcgcgaggagttaacgaccggacgtgctgcaacctagggatcaccaggcagctctttcgctctatagggatcgggggggcatagcacaattcctcttggaggggggg
ttggtttgcccgggtgactgatcctgccataatgtactcctacctatagcaccggcaaccgaagtaaagcatacataggacgatcttcatgcgtgaatagccgggaggaaagaaagggcggtagatgataatgaaaaaggcgcc
gcgtctgtacgggcgggcggaatggatgagcaaaaggtgccatggggtgaggaatatcccgagtctcatgtaagacaactaaatgcaccccgaggtgctgccgaggaactgggagaccttctcgagcacaggataggcaattgaa
agatagagctagcctattcgttatggggaatcaatgctccgggccgaataaagcttacctccggcacctggctttccccggcgcatattagcttagctgtgcttaataggtcggtttgtttggcttcctctcttagcccattcct
aaccagtggagaaaaggttcgcttatgctggagggtttttccacttagtgatcggtctgctagttcacgcaaatccgaagaagttatcacggacgagccacatgcagggaaacttgcacgtgtggttctggccgggctttcctg
aggtaTCTAATAACCTTGCTTCTGCTCGCCGCTGGCGCACCTCTCCTAACTATTGCCCATTTATTCTGGAATAATtTTTTTAGGAGGGACAATTTTACATATTTtTGCCAAATCTTTtTATTATTAAGTACGGCTGGTACCATTT

CGATGTGTTTCGATTtTTtCGACCAAGAGAGGTTTGATGCTTTTGAATTCATTGTATTAATTCtACTTtCTACTtGCGGTATGCTCTTTATGATCTCGGCTtATGATTTAATTGCCATGTATTTAGCTATTGAGCtTCAAAGTTT
ATGTTTTTATGTAATCGCAGCATCAAAAAGAAAGTCTGAATTTTCCACGGAAGCCGGCTtGAAATATTTGATCTTAGGTGCATTTTCtTCTGGAATATTATTGTTTGGgtacgaccggacaactaccgatatcaattaatatctt
tttttagaatgttgttgttaaatagataaatatctatttatattgtaaactatcggatcgggtattacttagatgtgaaactttaagacctcattagttgtgatattgatcttacggggggggggaacgaaatcaaagaatatat
agacttgtaaagaccctctatgtagttgtctatctagggcgatcgatctacatctttctccatagcccttgggctgtgtctcgatctcctacgcgcgaaatcagagggacctgtttctatggagattccccttggtctaattcca
cgccttatgacgaaaggagagtcggcgtggcatcaagtgaagatttagggaagtagagataaattcccttctggggtattccgaaggtgtaacctaggcaacgaaaagggcccgatacttcaactagaggagcgaccagttggt
tcaccaaacccctacccagcgtcacacgttctccaggtccgaagggatccagtgcccaactaccgactcctcccggaatttcgttatcggagccgagccgggaacggccgttagtggacacccactacttttcaccggttagaga
ggccctctttaatacattaagaaaagatgttcacaggggccagaaagactcggtcataggaacttagaccacctacgcgctggtaaacgaaaaccacctcgaccggatcagagtgaaacaacaatgtcgaaatcaggccgcccct
tgccttgaaagaattcacacgcggccacctgctttcccaaaagaaggggagatccgctgcttactgctcacggtttttttcgacctatactatagtaaagtcgtccgcctatctttcttctttctttttcttctattc...ggccc
accgagcgaagctctttctttttttttgggccgtgccgaaaatttgatcgtatgttagttagagaggttggcgaactactacgatcaatagattccccatctatatccaatcccaacggggaatagaagcgagtcgcttccggctg
gcttatagtcctagtcttttagcttatgtaatggtcggccttcctacacgctagagcccgccggaatgcctccttgttttagttttggacgaagccaagccgatacatacaatatacgataggcgaaggaaagacccccatttat
ttaatagcgcctgggcaagtttgatcgaggattggagaggtactttagtaactcgactgaagggagaagtgggaagaaaaggctcgggatggatttaggagtctttgtgcgagccgtatgcggtgagagtcgcacgtacggtaac
gaggggggggttcgcgtctatacgtgtagtgtggtggttgggcctacccaccctatTTGTTCCATGATCTATGGGTCTACTGGAGCTACCCACTTCGATCAATTAGCCAAGATTTTGACtGGATACGAAATCACTGGTGCTCGATC
TAGTGGTATTTTTATGGGGATTCTGTtTATCGCTGTAGGATTCCTATTCAAGATCACTGCAGTTCCTTTTCgggcggctgtaggacggacggccgcctataggtggtagggtagggtgggtggtaccgctcagattgcggccaat
cttcctaaccgcgcgcgggccgggcttagagcgagtgaaactcatcactacctcgtaagggcattgagaccatagcatgttacacgaggaaaaaaacccttgtagtgttgtcacacagctgcccgcctagaagagtcactcgctc
tgtagtgttgtcacacaagataagcacgtcgcccgcctgctggccgggcgaatcgaagttctcttccggtcaactgtccacccagtcaagtgcaaaaaacacagtagaatcacgcaacgcacgctgctggtgtgcttccagcccg
cgaggaaagaaagaagagcgacaaggttgagttcagatttgactgtttgcagcatgggagcagattacccaaaaaatccctgggaacaatgaaaaaaaaagatatctcggtaacaaaaaccatagggggctgtattggcgagatc
caacggtgaacagctgcccaaaataaaaaccgcctggaagtccgaggacctttagtaccgtaccccaaaccagcagccttcgcgccaagcaagaccgcccttgtccctctcctttcagtcgagtttgtgttcacaacctctcta
gacaagtgctcgagtgacttcgcccctctccttttagtcgagttcgtaaagcacctctcgcaataagctcgagttcctcttttataagagtaataaatacctgcagacctttcctttctccattccgcctcattcattgttttat
tccgcaaagcaaaagtggttcgtatgcctactttacctacttgacgaagggaacgcactttgttgagtttccgggtttttttatagattggattcagtcagcgtcacgacataatcaaaaggaaggagtgaggctttggttactg
gcgaaccgttccaaaggcgaccttctcgagtttccggctgtttcctagattgaaggaagtagccctttcgtcgccctaccaaacgcaagaagtcactatcaaacagctcgaaatactgtgacaaaggtgcgctcccccggtgact
aagaaagaggtttgcgcaacaattgaagtgatgaggtcgagggaagtagggctcctattgactaaagattggttcttcgctttcctttagaatgaaagtagctatgaagcccctacctacttcaatctttgtttgattcaaaagg
cgaacagcccccccaactagttgtatggggtggggttcttgtggtaagctgccttggatatgagaaattcctaaaaaaaggcaaagtccgctattagacgaagatcttcctatcaatagataggaattagtgttcgatatagggg
ataggatctatctgctctctctaaaaaaaaattgagagagcagatataacgatataaaatcggaaggatagatagacatctaaagaaagggatgtctattctattcctctttttttatagaaagaaaagatatctcgaacatcta
tcttttgtttatttatcattgattctatctatgaatcaagtcgaattcgtttttggccgaagccccgaatggattggatcgtttcccgccaatgaaatgaacgctggagcccgttccgaatgcttcttcaatccggaagttctcg
cgaagagaataagatgctgctccccccttcctctgtctttttttcgctttgctaatcttccccttccctctaacgggggccaggcggcggcgggcgcgggaggaaaaaacctaagagaagacgctcttaggtccttcttttttcagt
gcaacataggaaagcggccctctttttgtcatttctgcagctttccagatttggtattgaacgcatggcgtagctaggaccttaaaattatgtttgagcctatgttcaaacaaaacccatcccccggctggaaagaatgcccgcc
aagttctgataaggaaggaacaaccccggaaaggctcgacgaagggagggagatgcggcggggtaaggaaaaggctttcggagatcgagagatttcctttcttttgcatcgaaaacgaagaaggccgaggatagctacggtgcgt
gttatctgaagggagcacgcttttttcgaccgcggtgctatgattgccggagcctctcctcgaaccgcccgctggcctattgggatagcagcctgcggctttgcctgcccattagaataataatcaaaaattccggctcggcccgg
gaaagcgctggcaacaacagaaaggaaggggtccatgtagctgctgcgcccgcccctcttcttagtcaatggggcagcaggttcggcatctactagaaaagagagaatccacttcagaaaccacacctcggctaggcagcgtgtg
gaatggccgagagcggaccttttggatatataatccaagtcgagagtggagttacaggaacagccgtctgatggaaaaaaactttcacgttcggttcagagagcactttttttcgttgagaattcctcgttccctttcgtgtgaa
ttccccagtggcgaatttcaaacttgtgggtctattcaatctctcgagccccgaagaaacccccctcccccctcggactccatattccctttgactctatATATGTGGGCACCAGATATCTATGAGGGTTCACCCACCCCGGTTAC
AGCATTCCTTTCTATTGCGCCTAAAATCTCTATTTtTGCTAATATTTTACGTGTTTtTATTTATGGTTCCTATGGAGCTACATTGCAACAAATCTTCTTTTTCTGCAGCATTGCTTCTATGATCTTAGGAGCACTGGCCGCCATG
GCCCAAACGAAAGTCAAAAGACtTCTAGCTtATAGTTCAATTGGACATGTAGGTTATATTtGTAtTGGTTTCTCATGTGGAACCATAGAAGGAATTCAATCACTACTAATTGGTATCTTTATTTATGCATTAATGACGATGGATG
CATTCGCCATAGTTTtAGCATTACGGCAAACCCGTGTCAAATATATAGCGGATTTGGGCGCTCTAGCCAAAACGAATCCTATTTtGGCTATTACCTTCTCCATTACTATGTTCTCATACGCAGGAATACCCCCGTTAGCCGGCTT
TTGTAGCAAATTCTATTTGTTCTTCGCCGCTTTGGGTTGTGGGGCTTACTTTCTAGCCttAGTGGGAGTAGTGACTAGCGTTATAGGTtgggcggccggaaggttgccacgaataagtaagtttggaggaccgaaggcagttctc
cgtgcaccggacacgtagcttaccgaatcagttgcgacaccgatgggaatgcatgctacgaaagataggtcgagtctgatacatcaaccgtctactcaatatccttagtagagtccacaatcactacacgagatgaaccttggt
ttggtgaattgaagttcgccttaggtgtaataggactcccagttactgcgcgcgatcgtatactgagatgctccccgccggttgttggaacgacacgagccgggacgggcctggattcagaaagatgaagggccccaaagtagaa
ataggggggttacaaattccccatctcattggggcggaaaacgaatcgacatctcgatgtgatacagccttttcttttttagttgggaaagaacggcgaagtccatccgaaccgaaccgtccaatgaagaataagaggagagcaa
agcccaatggggaagcgcatgcggagcaggcacggagaaaaatcagtgtggaggagaagcagccgagctcattcccttcgcatcctgggcccaaagcagtgcagtctttcctggccaaatcaaggatttggggcttcttgctacg
atacttaactatataaatcctttagtaatatataaatggaaagatagatccatccatctatcctatccgatttccattttcatatctaaaaaagaatcgatttcattcaacgtttgattcaaagaactgcgcctagcccccccgc
tcatgaaacggctctgctgcaatggatggcagagggtccgtagtacccaaagcactggagtgatccagtagccgggaaggggcctagaagtgcctactactactacaccatactacacttggctctacacatttaccgagctaac
ccctgtcagtgcctggcagagctaagggggcttcaatccttattccttatccccatcttcgcccaggctaacggggccttacttattcaggggggggagcctcgagaagcactgttgagaagaagatcctaggcccctcttcattc
tctacagggtttctttcttcaatataggtgacaacgagcgaggcagagatggaagagatcaaagacgagaataagaagccagtaaacttccttttttgatcattttgtttgatagaggggggatagaaaaaatggacaaaacctgga
ctcgcatttctcatcgaacaaatacgaaaaaagaatcatattgaaaacctcacctctcctctcgtttcactctcgttcctttccttacagtcgagctcctttgttcctatggacctctcgcaacaagtgctcgagtcatgacttc
gccctctccttttcagtcgagatcttttaacctttagtcgagtgcctttccggctctcccaaggataaggtctcattaattagttgatgaggggtcgagttactgcgttcctaacctaaccccttccttctccgagctttgtat
tataagtgatccgaacctgcccggagtgagcctcccatagaggccaagttggtgagccgtatgatgggcaactatctcctgcggtttggagaggactcagctgttagttagtatccccttggtttcggggtggacccttttttcac
tctatTTTTATTATATACGCTTAGTGAAAAGAATGTTTTTTGATACACCTAGGACATGGATTCTATATGAACCAATGGATCGTAATAAGTCGTTACTACTAGCAATGACTTCCTTTTTCATTACTTtATttTTGCTATACCCCTC
TCCTTTGTTCTCAGTTACTCATCAAATGGCACTCAGTTtATATCTTTAA

nad4

ATGTTAGAACATTTCTGTGAATGCTATTtTAATCTAAGTGGTCTTATTCTGTGTCCTGTGCTAGGAAGCATTAtTCTTCTTTTtATTCCAAATTCAAGAATACGACtGATACGATTAATTGGTtTGTGTGCCTCTCTTATTACTT
TTTTGTATTCTCtTGTTCtTtGGATACAATTCGACTCTTCTACGGCCAAATtTCAATTTGTGGAAAGCCTTCGATGGCTTCCTTATGAAAACATCAATTTTTATTTGGGTATAGACGGTATCTCTTTATTCTTCGTGATATTGAC
CACATTTCTGATCCCTATTTGCATTTtAGTGGGTTGGTCTGGTATGAGAAGTTATGGGAAAGAGTATATTAtAGCATTTTTAATTtGTGAATTTCTAATGATCGCCGTGTTttGCATGCTAGATCTTtTACTATTCTATGTTTTT
ttCGAAAGCGTGCtAATCCCTATGTTgtgcggagcggagtatcttatattcgctgggagaaagcttttcctctgcaggggccttgtgcagtaaacccctacgggcggtcgtccgtcgtcgtaaagtagtccccgcgaagctttcg
ggaagaggggtagtcttgtgtgtaagcatagcatttctggtcgaacccgcccaatccaactaagaagaaccgaacctgacaaacacatctttttccttttgggagggtactccgagtatagtgggtacctcgtaggacctcgacc
cgcctactcgggtcttgtatggatatgcaggaaggggtgctcctaggtgtgtgtaggggttgtgtttgttcgcgagaatggattcctcgtcaagtctgtttggggggtgtggacacacttgcgcgaattcaggtaacggctacaa
gggagaaataaaaaggaaactgtacccgaccagggatggacgtaaactcgtaagctaccgaggttagggataatcgtccaggtcttattgtgaaacaaaaaagccgccccgccacagcaagcgggttggttcctctgtcgtcgcc
ggatagctcttggcgaggtacttttgtcactcgactgaaaggagaggagaccttaggataagttgctaaaacaaaggggagtagaggatcgacccgttcagtagaattccgaagaaagactgttgacagctggtggagacatttc
tttggccccggcaaatcaaaaggaaatgcgggcaggtttaagctcggcagagggttcaagaatagggtcctgcccttcagattctcagaataaaaaaatagttccaaacctttatgcatgcacctccgtataagtgctgcgtac
aagttccggccaggataattgggaaagatcaaacccgaaagaaccgctcacatcacagtagtagtagcgtaaaggccgtaagtcgggtagcggccataccataaggtaaggggctattactttcacatctctccttctgtcgtac
tataagaaagagagatccgctgcgtgagcaacccgactgtgcgttacatgtgctctacaggccgaactccatctttcttcttaacaagcccatttatctttagattttgaagacgggcgttgcgttcggttcgaaaggtatggtt
ttcagtatgtctccagatagggcgccccactagtccggctagctagtgagcggttctttcgggcgagaagcaggccgggccctacgggcgggggcatctcccgcaacgaaagctgcatagttcgccaccacccgaaaagtaaaag
attagagagtccagactaaaaatacatgcatagatagtgatctaatgacaagggccgacgacggaagctcgggacggagccgtatgatgcggaagtctcacgtacggttccctgagaagggagtggctacctactggagcttcga
ccaagcaccccccggtcaattccgctttggggccacccccttactctacCATTATTATAGGAGTATGGGGTTCGAGACAAAGAAAGATCAAGGCAGCATATCAGTTTTTCCTTTATACTTTACTTGGATCTCTTTTTATGCTATTAG
CTATTCTGTTGATTCTTTTCCAAACAGGAACCACCGATTTACAAATATtATTAACCACAGAATTTAGTGAGCGGCGCCAAATCTTTCTATGGATTGCTTtTTTCGCCTCTTTCGCCGTCAAAGTGCCTATGGTACCAGTTCATAT
TTGGTTACCTGAAGCTCATGTAGAGGCACCTACGGCAGGATCCGTCATCTTGGCAGGAATTCtTTTAAAATTTGGAACCtACGGGTTTTTAAGATTTTCAATACCCATGTTTCCCGAAGCGACACTTTGTTtTACTCCTTTCATT
TATACTTTAAGCGCGATTGCTATAATATATACTTCCTTGACCACTTtAAGACAGATCGATCTAAAGAAGATCATTGCTTACTCCTCAGTAGCCCATATGAATCTGGTGACTATTGGTATGTTTAGTCgggcggcggccgttaggt
cacctattttgagttatggacacacaaggccaaaacatgtgtgtcgggcgtgcgacccatcaacctactagcaatgggggagaaaacatagcatgtcgcaacaaaagcttgattcgaggcgtcagcaaaacactgccgtctgttc
ccttcagtcccttagcgccccgggacgggagtgggggacggctctacgcgcaggcaacagcagcaccggctccacgaagtctgaatcgaatctttctgttggctttcccaaattcattcgtaaatcaaaatcaaagggctagaag
cgagcgcttctagctgcttcgcctgcttcttcttattatggcggccatgttggcgtggcgaaaatgaacgaaaacgcgagatgaacgtgctattttcaaatcggattgatagattgatctgttctgatagatctaaagagtagaaa
tagatagagaatagagagggaatcaataataaggtctttgagcctatttctatctattgatgagacaactatctattcttgatccatcagaaagaaattatttatccatcagaaagaaatcgatatgtatctgatggagtcttct
acatcgtacgtagagcgcccaagcgctttttgggccagctcagttctcttatccatcggtccaatgcactgggctcatctcatggagggaaaagccaaaatgtagttgtcttgttgttgttcgccgcctcgacgcattcccttct
ctccccggcatcgtcccacacagaaagaaagagcgcggagccccggcccgagccgtaggtccgctaacgtaaagcgaggagttgagcctgaactggcgaaccgaagtcactttcggaaccatacttcctacagctgctaacatgt
gcccagtcctgcggaaaggcgcaaacgaacgtgagctgctataccggaatcccccgctggccatcggggaccgagtggtaaggccatgatctgcaggggaacggatcactcattcttccattggggacaggtgcacgaacgacaa
ctccaaacgtcacacatccgccgcctacttaccgtttaggtggcaccagcgagatccagctaaggaaaaagagtgtcgcggctgctccactccgccgcggtctcatgaacttcacttcgttgccttcccgcgcgcaaagcgaatg
ggcgctgtgctgtgggtcagtttcggggcggggggcgcagagataccagaataaatgattcatttgtttggatcgacgagctttttcagccccaaaactcagaatcaatggaatgtctgtccataaacatctattctatctgtat
atataggggatctctctatacatatcaaagtcttttatggcattgatatgatcgcctagctgtagccgcatatcacagctgcactcaatatgcggatttctgttggatcagatcttttctttcgctttgacggaagctttttgac
ctagcgaaaagcactttcgcgcaagcaaagctttgcgaagcaagacgaggaagtggagctgtcgtataagcggtagcttcccccgaccgactaaaatacaagagtcgcggcctactttgattgcgaaggggtttggc
aacaagcaaacggctttctatcatagttgcaagggttcaaaaccttagttcgctgcttttcccagtaccagagaaggggcttatactgctcgcctttgtttggtttgatatattcattcagtcaaaatacaaactacaacaaagtg
gaagtggaagggccgctatagaagctagaacttgctttataagtcggcctaaccaaagcgtcacgacaacaaaaaattatccttatgaatggaatcttaagtaggggggggctttgaccgcccgcctaccaatcaaagaggcagag
agtaaacgtaagctcacccgtaagctcgaagagagcttcccttcattcgcttcgcgggagccgcacaagcacatagctggaagtcagagggcccatactacctgcctaaccctttgttccgaggaccgtagatcggaaagcacc
ccatttatccaaaagagaagggaagggggcctatgtatttgcatgacccctgcggatttgaccctatcccggagccaatcccctattggtcctgccaccacgccgcagaacgagagctcgtgtggaacctttttctttctggcgtaa
cagccggtggaacgtaacaaaagattacggtcgcctaacattaacataagggcgggggggtacggtaaactcggccaaaatatgacacccgaagggcccgaacgcacaatcctatcccatccgagtccgagtttaccccttgcact
tcggacagccgaccgtagcatcataaggaggacccccctttcgaggtaaaaaaaaaaaaaaggtacggtacatataggaggttggactttctcaacgtggtgtatagcacgaaaaacttttcgatacaagaaagggccgttctcaca
tgaaagaagagaagaaatcctttcttctcttctttctctttctcgagaaggaaagaaagaggatggggggaggggggggaatggggccggtgcccttcttttacggccgtcactcttatttgtcagccgtgaggaactaccggctc
ggtctcggtgggaaaggaaaggcttgggcctacctatcccgataagacctcataaaggaacggcgggagtgataggttccatattgccgagctgaagggcaagacttttgtacgtgatcgtagtatgtgacgtcgtctcgtccac
gctgcattgaagagtacctacgcactaagttccggttcactgataaggaagatagagttgggcgggggtctacgatgtgatactcaagtatatgacccggggagatacatgctaactatgggtaggaagcaggaacccctatgta
aataatttcgggggggggttacagatctcttatactaccctcgatcgacagagcggaacgaccagaaaaataagtgatgttagaaagccgtatgataggtggtaactatcttgtacggttcgggggggtaatcggcgtactccggg
agaaatctttcgctctattGAACATACAGGGAATTGGAGGTAGCATTCTACtGATGTTAAGTCATGGACTGGTTtCTTCAGCCCTTTTTCTATGTGTTGGTGTTTTATATGACCGACATAAGACTCGACTTGTTAGATATTAtGG
AGGTTTAGTGAGCACCATGCCGAATtTCTCTACCATTTTCTTTTtTTTTACTTTGGCCAATATGAGTTtACCTGGTACTAGCAGCTTTATCGGGGAATTTCTCATCTTAGTAGGAGCTTTCCAAAGAAATAGCTTAGTAGCCACA
TTAGCAGCGCTTGGGATGATTTTAGGTGCGGCCTATTCCCTTTGGCTATATAATCGTGTGGTTTCTGGAAATTTAAAACCTGATTTCCTCCATAAATTCTCCGATTtAAATGGCAGAGAAGTTTtCATATTTATACCTTTTCTTG
TTGGAggggcgaccgtccgttgaactaccaaagaaaaagggtaaacctatgtgatcatgacattgtaggtgcttgcgatgggacggatgcgacttccctcagttggtttgggtggcatagcccgttgcataagtccccctttttt
tgattcatttttgagtctttagggagccaaagctttactttactaataaaggctcgcgcaggggcgctcactttttttttgctaagccgtctctttctgggtgggaccgagagaaataaaggacagagggcaaccatgcatggta
cttctcgaccctgtctccgagggacagttgaacgagcgactcatgaatgctgccgggtcggacgagccaataactcgaacgcgttcggtctgtttttttgagcaagaatcacagcgttaccttaccttcaccatgatacggactcc
aagttcttatggcagagcacgaggagatttatcatcaatattctaatgggaatggaaaccagaagcacgaccgaggtcttcgtagtctaaaataataaaaccatcaataacagtaatgtaagcatgagacttttttggtagtaccg
gtgaaccagatggccgcggcgatggaatctgggacggaggactcgtagtatctctctagatccggcaaaagcgaaagacccccctagttccattcgaatgaaggctgactgctgagcccactctggccccggcgggccccccgtggt
tgcgagccggagctgccatagcttatggctagagcaatgggagggggcctcagcagagagaacagaacagaaccaccgtaaggataacgagtgctccgcccgtcaagcgggcggcaggagcagcaggcaagtacttggtaggccaa
cagtccagtgggcactcgacgaaaggggggcacacggagcaagtacgagaaattggccccgctccgctttataaaaagcaaggacccactacgggaggtcaaaaacccaggacctatggaagtcggggctcgtccccggtcaatat

tggatcaaacaaaacaagcaataggggcccgtagcactgacctctttttttattgattcaatataataggggaaaagatcgtacagttccctaccgagacaagagacactcttaacagatcctccgcgcgctgggcatacctctt
ccgtgcgtctttctcgtggcaggaacagaacaacagggaaagaaagacccggggccgacctgcccagggctcgagggcgagctttatttaagagagaatggggagcgaatcgaaaggcttccgttttagttcttggtttggttcg
ggctcctctcgatctttttcgtagtagggaaggggaaggagtctaaatctatggacattaatgaaccatcattgatggacgttgcacatgacacgatcaattcgactcagggtccggcgctaatagaggttgcttactttccta
gtagcgaaggaaaagggcagggctttttttcgtggtaatagtgggcgggtctcctttcgaagtaaaggccttcgcattcctaatccgccccaccaaccccggacggcttagtttgtcccagcttggtgaatcgcatccccgcgcaa
cgacatagtttgtgcgccctttaccgttctcgctcagtgtttgcaacggctggggaggcagtcgtagaagcgaagtctatcgccacgccaaccatcaaatacgagattggcccccttctcaaagatttgatggaatggcccaccc
aatagcgcttatgtcatatgggaactcatggctggaaacaatccttatggttttgatatccggtaggaataataagaatcaaagtccaggtaggttggtgagcctagtgataggagactatctagcttggttcggagagcacttg
ttgggttaaaaactttttttgttgctaaatgttacagcctaaatgctgaactattgactctaCTCGTTtGGATGGGTGTTtACCCCAAAGTGTTCCtGGACTGCATGCATACATCCGTAAGTAACTTAGTGCAACATGGCAAATT
TCATTGA

nad5

ATGTATCTACTTATCGTATTTTTGCCCCTGCTCGGTAGTTCCGTAGCAGGTTTTTTCGGACGTTTTCTAGGATCAGAAGGAAGCGCTATAATGACCACTACGTGCGTTTCATTCTCTTCGATCTTATCTTTGATTGCTTTTTATG
AAGTCGCACtGGGAGCTAGTGCTTGCTATCTAAGAATTGCTCCATGGATCTCATCGGAAATGTTTGATGCTTCTTGGGGCTTCTTtggcgaccgtgaagtcaccggatgaattgccgagtagatagatctgatccggacgcggct
gttgctccgcggcgatacggactcgacccgctcctacccaccccggggcaccatagcatgtcgggaataagggggacatactggacgtaaccactccctttggttgtgggaaattcctgcctttcaatcgatacacagttgagt
aggccgatcacgaacgctacaggtgtgggagcgatcctggtcagggaaggctaagacggcgccctgcatatgggtagcaagagggcgcttatgccccgacggtggggccttatgggaagggcccagccaatagggacagcacac
cccccgcttcaagcgcacctctgtatcgactgaataactctttttggtctagtcggtggaaccggtgaaccacgcgagctggttagatgcgtggggcagagggctcgtagtaccccctttttcttgatccagcctttttcttcgcgc
ttcggtagtgaattacctactcaaaaaatagggaggcctgcacgccctatttgtgagactactaaggcggtggtgactcttggaagggaagaaggggcctaagcacggatttttttcgtacactttagtggcaatcgtacctgttt
ttctaggcctgtttggacatacggttcccgccggaagatcaagttggtgagccgtgtgatgggaaaccttcccgcacggttcggagagcactgaattagaatgagaggttcaccaccacatcattgcatgcaaggggagctcgct
cgattcgcaaataggtccgactcgtaattcacttctgactctgtGTTCGATAGCCtGACCGTAGTGATGTTAATTGTGGTTACATttATAAGTAGCTTGGTCCATCTTTATTCCATTTCATATATGTCCGAGGATCCGCATAGCC
CTCGATTTATGTGTTATTTATCCATTtTTACTTTTTTTATGCtAATGTTGGTGACTGGAGATAACTtTCTTCAATTATTCCTGGGATGGGAGGGAGTAGGTCTTGCTTCATATTTGTTAATTCATTTTTGGTTTACACGACTTCA
GGCAGATAAAGCAGCTAtAAAAGCTATGCTTGTCAATCGAGTAGGTGATTTTGGATTAGCTCTTGGGATTTtGGGTtGTTTTACTCTCTTTCAAACAGTAGACTTTTCAACCATTTTTGCTtGTGCTAGTGttCCCAGAAATTCT
TGGATTTtTTGCAATATGAGATTGAATGCCATAAGTCTTATTTGTATTTTACTTtTTATTGGTGCTGTTGGGAAATCCGCGCAGATAGGATtGCATACTTGGTtACCCGATGCTATGGAGGGTCCCACTCCAGTATCCGCTTtGA
TTCATGCAGCTACTATGGTAACAGCTGGCGTTTTCATGATAGCAAGGTGtTCCCCTTTATTTGAATAtCCACCTACGGCTTTGATTGTTATTACTTtTGCAGGAGCTAtGACGTCATTCCTTGCGGCAACCACTGGAATATTACA
GAACGATCTAAAGAGGGTCATCGCTTATTCAACTTGTAGTCAATTAGGCTATATGATCTTTGCTTGCGGCATCTCTAACTATTCGGTTAGCGTCTTTCACTTAATGAATCACGCCTTTTTCAAAGCATTACTATTCCTGAGTGCT
GGTTCGGTGATTCATGCCATGTCGGATGAGCAAGATATGCGGAAGATGGGGGGGCTTGCCTCCTCCTTCCCTTTGACCTATGCCATGATGCTCATAGGCAGCTTATCTCTAATTGGATTTCCTTTTCTAACTGGATTTTATTCCA
AAGATGTGATCTTAGAGCTCGCTTACACTAAGTATACCATCAGTGGGAACTTTGCTTTCTGGTTGGGAAGTGTtTCTGTCCTTTTCACTTCTTATTACTCTTTTCGTTTACTTTTTCTAACATTTCTAGTACCAACTAATTCGTT
CGGGCGAGACATCTtACGATGTCATGATGCGCCCATTCCTATGGCCATTCCTTTAATACTTCTGGCTCTCGGGAGTCTCTTTGTAGGATACTTGGCCAAAggagaaagatattaagcgggtccagaggttgacgcggaacttcga
taacgcggaggatcccgcccgtcgtctggaagtagccgcgcgcctggatccggaggtgcgcgagctagatcagaagtgggcactctttcaagaaaaggatgcctccgggctaggcagatagccgaatggagggagacgttttcgg
aggatttagcggcgggaagggagaaagaagcccgccttgcattactaaattcgtggctcaaggtgatcatccatacacggaataaccaacccccccaaaactaaagctttcgttctcttcttttttttttacttgattggcaggtg
tacaacaaccttaaccgcgcaagcctgggctgggcctacctccatccttagaggagccgtatgaggcggaagccccacgtacggttttgaagccgagcctttccagcaatggggcttagggacc...gggcggccggcctactac
gggcacccgcctgtagggattagtgcgtgagaccgcgatccacaaactgacgcatgggactcacccttttacttgagaataaagaggggaaagatagcatgtcccaagagcgaggcgagttttggaaccctacagcgggagggacg
cctcgcgagccgggcttccagagatgaggccttttggcgaagccaagtttctttcgggccaccaaaccctgcaactgatgagaaagaaggccctatggagtaaagggaagcgtgtacgttgtcacactccctgccttccaaaggt
gcctagaggacgggccagacgcagcggagcgacaacccgggagcagagtccccaccggcaggggggacaggagacggccatctcaaggcacatcacgacctacaggtaagaccggcgagaccagggaaggcaacccgattgggagt
cagaggatccatagtacctgcagcccGATATGATGATTGGTTTAGGTACgggcctagctactgataggaatggtaacccgatagatcttaatgattcgctcccccccctcttcgttgttatacggagaaatagagagttccgcctc
tgtgcgagcgcgggatctcgaattggagaaagatattaagcgggtccagaggttgacgcggaacttcgataacgcggaggatcccgcccgtcgtctggaagtagccgcgcgcctggatccggaggtgcgcgagctagatcagaag
tgggcactctttcaagaaaaggatgcctccgggctaggcagatagccgaatggagggagacgttttcggaggatttagcggcgggaagggagaaagaagcccgccttgcattactaaattcgtggctcaaggtgatcatccatac
acggaataaccaacccccccaaaactaaagctttcgttctcttcttttttttttacttgattggcaggtgtacaacaaccttaaccgcgcaagcctgggctgggcctacctccatccttagaggagccgtatgaggcggaagcccc
acgtacggttttgaagccgagcctttccagcaatggggcttagggacc...cagtcgtctcatagagcactgcggaaatcgaagtcaaggggtaaggtgtagtcagcttccccgctcacgtttcggttgtcaaggtcagattctt
taaacaacagtcctagaggccgattgattgaccgagatggggaatcaattgcctagtcaaatcaaagtaaagtagagtagttaccgttgcttgtttgctccttcgtataggttccgtgtaggcgtctttccatacgatcaattta
atattggaaaaagaaaatccggtgggtttttatcgtgagctcctttcgcgagtcgataccttgctcgccattattataatggcgggaaccatattccttttccctttcaaactcaaaaggaaaaagatgctatctattccattat
gcggcaataccgccgcgaggagtagagtgagtccatgcttcctaattttctgactgaaaagtcttttggctcgatgcgcttaacaaaagggaactctctagattaagaatgaatttccttctccattaagccggaccggcgacga
ataataaataggtacgtaccccccaccccaggcagagttagtgagccgtgtaataggcgaccatttcgcgcggttcgggggcacttgagtaagccgccggcgcccgactgcgtcttgacccctatCAATTTTTGGGCCAATTCCC
tCtTCGTACTACCAAAAAATGAGATTCTTGCCGAATCCGAGTTTGCTGCTCCAACCATTAtCAAACTAATACCTATTCTGTTTAGTACTTtAGGTGCTTTTGTTGCGTATAATGTAAATCtCGTAGCGGATCAATTCCAACGAGC
CTTTCAAACTAGTACTTTTTGTAATCGACTCTATAGCTTCTTCAATAAACGCTGGTTCTTCGATCAAGTTTTGAATGACTTTCTAGTCAGATCGTTCCTGCGTTTCGGATATGAAGTCTCATTCGAAGCTTTAGACAAAGGTGCT
ATTGAGATATTGGGCCCTTATGGTATCTCGTACACATTCCGACGATTGGCCGAGCGAATAAGTCAACTTCAAAGTGGATTTGTTgtgcgaagagtgcgttatgacccgtggccgcctgcctggtgggggccctcctccgttgtg
ggtaaacgggaaacccgactctacgaacccgaggaaaggctgcacagcagtagtaagggcgttaagaccgagctttttgtagtgctagcaggaatgcaagtgaatgaatcccatcccctagcgagtgaagtgcttacgctccggg
ggcagcaaaagagtccgttcttcaatttggagatctttagattcgtaagatcctgatagagtccccttccctttactaatataatagagaaggggctgaaaagcagttgcttgctgtctacttcgcgagccttcactgcaccgtt
ctgtaacttgccttctttcgtccgtccacgaggctgtaaaaagagagataggggcggctatctagcgggagtcgtttcggttgtatgccacgaggtccctatggacaaggggacaagtgaatcatcgcttttgggcgctggcatc
cctctaccatccatcccatagcattccatcgtctcgtattgtactgtaccgtatccagaccagatagatctattgagtgagagaaaggtagtgtaactaattagatattcttcatattcttattgatagggatccccattcattc
ctagattcccgtcattacggatgtccctacctaactacatggtagtggtctagggagcgcaattgctagagcacgggagaatgaagagtaatgtatttcagcaagagcagccggacggactattctagtgagtctatgactacta

gagtagagttgagtcaaaggtatggtatagcagcctttccgtgcgtgcctctgggatctcctgtaaacccccatgatgtggtaaaggaggatattaggggaagcagtgagtggagattcccctgcggagagccggatgaggggag
accttcacgtccggttcggagggcggggatatcccgaccctacTATCATTATGCCTTTGCAATGTTACTTGGTTtAACTCTATTTGTGACCTTTTtTtGTATGTGGGAtTCTCTATCTTCTTGGGTAGATAATCGATtGTCTTTC
ATTTTGATAGTGAGTAGTTTTTATACAAAGTCAAGTCAAGAATAA

nad7

ATGACGACTAGGAAAAGGCAAATtAAAAATTTTACTTtGAATTTTGGACCTCAACATCCTGCTGCTCATGGTGTTTCACGATTAGTATTGGAAATGAACGGAGAAGTGGTGGAACGTGCGGAACCAtATATTGGATCACTCCAgt
gcggcacgaagccgctgacgccgagtcggctcctatgccgctagctatgccctgcttggtcccccggcacggtggaggttccgtagcgggtcatgagcaccgggctaaggggcgaagtcactcgactgaaaagagaggggcggtt
gagcaactcaagcgaaccgccctaccttactacaacatagggacagagggggagaaggttgtgaaggtggcctcgttatccacacctccggtcggatgaatggaggaccgcccgacccgggtttcatgagcgttggcgggtcctg
gagtgcctgtcaagggcgctagcgcataccccggggtgatcatcatcacctgcacctcacatctcggcgtagtggaacgtgtaacccgcctgctgtctcattcaactacatttgttactgtaatctatagcctaacagaaggcag
cgtcgaggggctttaggaactcgactgaaaggagaggagataaattcccatacagccagcggggaggatggcactacaggcaaagaccgtctggcgaaaacgccgcaggcgcgaagcgtggtaggcctgcgccgggtgagcatag
gggggaaagggatcccggacggtgtaagagccaggggaggccgggtcatttgacggaaatggaaggcttttcccctcttatagaaagccctatgaagttaagggaagtgaatgaattcttggaaaaagaaggagcgagcctata
tataaaatgtaagaaagtcaattattcaatgaatagatgataaagtcaaccgtacgacagacagcgctgcctacacgcgaattagcttccaaggtcgagcagtctcaatttcactacaggatttgcgaatgaatgctgggctggg
ccacctcgaatggcgtgagcgcatgcggggagacccgcacgtacggtttttaggggatctggccgaaagaccggccggcgcccacccgacTAGAGGGACTGAGAAATTAATAGAGTACAAAACTTATCTTCAAGCTTTACCTTA
TTCTGATCGTTCAGAgggcgatcgcggagtcactgaatgaagtcctccgtttctttcggaggtgctgacccgcagcgaggcagagatgactaagtgacatatggaatatgacgacaacaacagcatgtcgtagaaggagagaaca
ggtggagctaacgacccacgttgactaacgtatctacaactacatccccgagcggcagtcaaacggaggcgtgaatgcaagatgccagcggaatgatcggccggacagaggctagggctgcttccttcccaccgcgtccttcctt
gtgtatcggagatataaagcgagtgcaccggaaaagaacgggaactgggtcgatctattgcgaagcatccgaagcataactgcacactcacacgatctttgccgagagataggagcattcggtggaaccggtgaactacacttgc
ttctggatagatgtgtgggacagagggctcgtggtaccttctgcccacccttcctcctctgctttgagaactgtgtgaacggagagtgggcagaagggaaggaggtcctcatacagagaaaatcatggaatgggtcgagatagat
gacagcgccttttttcctctcttccttgccgggagggcaatcttctcttatggtcttcacctcccgcccggcctggaaattgaatccagccccttctttctgatccattcatttctgcaagcccagagcgttgcctcccttctat
tgcataacctaaaaagctataagcaaagtaacaaaagcgcgctccgcccggtgactaagaaagaggtttgcgcaacaattaaagtgataaggtcgagggaagtagggctcctattgaaaggctttccctccctcaaaagaagact
agctttcaatactagttcttacgttacgctgccatttttccaatattattgaatagcatggcctggggctaaagtaactcaagtgggagagccgtgttatgggtgaccttattgcacggttcagagagcacttgtgtatgtgatg
caagtgaacgtgtacgaaaaagctgtcgtaaagtttcgtttttcgttccgttttcgaccctattTATGTTTCTATGATGGCCCAAGAACACGCTtATTCTTtAGCTGTAGAGAAACTTTTGAATTGCGAGGTACCATTACGAGCT
CAATATATACGAGTGTTATTCCGTGAAATAACTCGAATTTtAAATCATTtACTTGCTTTAACTACTCATGCTATGGATGTGGGAGCATTAACTCCGTTCCTGTGGGCTTTTGAGGAGCGGGAGAAATTGTTGGAATTCTATGAAA
GAGTCTCGGGAGCCAGGATGCATGCCAGTTTCATACGACCAGGTGGAGTGGCACAAGATCTGCCTCTTGGCTTATGTCGAGATATTGATTCCTTCACACAACAATTTGCTTCTCGTATCGATGAATTAGAAGAGATGTtAACCGG
CAACCGTATCTGGAAACAACGATTAGTGGATATTGGTACTGTCACTGCACAGCAAGCAAAGGATTGGGGATTCAGTGGTGTAATGTTAAGAGGTcgtgcgacatgaagacattgatagcaatatggggaagttcccatcaggca
acaatggttccgcctgactctacttaagcatgcatattatgtaagtgaagacttggtgtgaagccttggagcttacgttagaagagcaaaaggcccggggctagggtgagctggggggggacagcgtaagtgagcgaatgtgtgt
aagcccagtcaaagatgactgttctaagcgggggagccacccacctttgaatggtgttggtcctacggaccgtgaacggatttcgcctctggcctctgggcacgtcggaaccgcgtgagttcaccggggtggagcacggtccgc
caaaaccggcatagattaggtgctattgatggaacatggtaagcctatctttctccatatggaagtgctgcgagcacttagagatgcgggtagaggaagcctcaaaaagcgaaggccgagctgtaggtcacgtgacctgcaccga
gttggtggctgactgggctttttccttgatcaaagcagatcaactcgccttctttcttgttacccaaaactaaagttggtcgaatggtttttttcctgccccggaacgtcgaatgaaatagggggccgggttctctttctacaac
cctttgatatgataggcctggctacctttcccctatcccttatgattaggggggctgttaagcccaagaacaaccagtcttgtggtggtacggaaggaacggactccgcgaacgtcccgcgccccggaaagaaagtctcaaccag
aaccacattccttttgcgtgcggatgtagctaagtgtctgactctattggtcatagtttcctgctgttgcggctggtgctcgtttgcgcgcgcgtgaaccaactcaacaaagaaggaaaggatgcccggggaggcatctgagaat
gattcgagccgtatgaagggaaactctcacgtacagtttgttttttttgggggggcaggagcccgacagggtcccccactgacttggcccgggcctaagttaaagtgaagtggtgggcctacccatcccaaCtAGGGGTATGCT
GGGATTtGCGAAGAGCAGCACCTTACGATGTTtATGACCAATtGGATtTTGACGTACCAGTAGGTACCAGAGGAGATtGCTATGATCGTTACTGTATtCGTATtGAAGAGATGCGACAAAGTCTTCGGATCATTGTGCAATGTCT
TAATCAAATGCCTAGCGGCATGATCAAAGCCGATGATCGTAAGCTATGTCCTCCATCACGATGTCGAATGAAACTATCCATGGAATCgtgcgtcgtgtgaaacgtagatcatcgccgttcttaaccaagactcaggttaagctcc
gtctcggaaccttgtgggttaggagtaaagcatcccggggttggcgcatctcattgggcgtagagaagcattgggaaccccaatttatttcttcggagccgtttcttttcccgtccccccacccccggcatagcgcttcgcttcc
ggttcttcggaagaatcaacttacttctaccttcttcattgatctggggaaaaggaaccgtctaccagttgggaagctagacatcaagtaagtggcttgatgaggataactaagctgacacgccggagttggctgctggcacaa
cagggtggtgccttaccgcaccgcaggcgaacgcgcggtagcgttcgtggtggtgcttcaggattccaatgtactgcgtccaagatcagaacgagcttgccggcggaccactgccgtcccattcttgagtgagctggagcgcagc
catcttatccactgaactagctagaagctatcgcttcgggtcgaagcactaaaagaaaagcaccgggaaacgcggcggcataggaaccacgggacccccaccctactagtaaagggaaaacggaagtgcgctcctgcgcaccagc
tgaaaaaagccctttcccctttctctgataataaggaaagcttcttagctccaacctatacaaggggtttttatgtccttttttataggttgggttgttggatacgggatcctcgtagtaggctggaccaacatccagccgagaga
gggcagcctctagaagcaacaggttgggaaaccaagagaacgcttcgccttttcttatcttctttctgccctaggagtagaagtagcacaaaaagagggattcgcattattgacccaatgataaaccactaacaccttcctcgtt
ggggctccgcgcactgggaaaacgcttgacgcgattgggaaaccggccactagttacaaagctccaataaggtatcgagagggctatcacagtcaggtgcgaagaattacccctatttggaaagtacccttcttcctatttaggg
ggttaaggcgagaaatggcttgatgaatcgttccgttcgccatgcaccggccccattcacttgcttatcgtagaggctgtaagtacacagtgccccacaactatcaatagtatagtggggttgaaagacgagagtgcccgccctt
tcttttctttcaagtgggccactttttttcccgaacgcagtccgggatcaccgtggccgtgtatatatatatatatatcttcgatgctgtcatttcgaaatgtccgcttcaatcgctctttcacctcccccaaaaaaagcaaagt
tggcttaacgagcgcagatgtgaggaagcgggagcaataaaacaaaaaaatatctttcttgtccttctacttaaggggcaaagagaagcgcttttgctactgagaaagcgaacggtcagcgcgaaggttcaagactttctgagc
gttagcgaagctagattctcatagcgaggcgcttcgagttagcgaagcctgtagtagcgccgaagccctatgtgctataatgctgagccaaggacactccgccttattcttataagaagcagtcaactgagttctgaacgaatta
gatccttggtaaatggctcaatctatagatagaaagccttatgatgggaaactaccacgttaggtttggagagagatgggaccggttatataatagagggagcagatgcaagctttttttctttcaatagccggccaaatgactac
aggatcatcggtctactctacCTCAATTCACCATTTCGAACTTTATACAGAAGGTTTTTtCGTACCAGCTTCTTCTACCTATACCGCAGTTGAAGCACCTAAAGGAGAATTTGGTGTCTTTCTGGTCAGTAATGGAAGCAATCGT
CCtTACCGTtGTAAAATAAGAGCACCCGGCTtTGCCCATTtACAAGGACTCGATTtTATGTCCAAACATCACATGCtAGCAGATGTGGTtACCATCATAGGTACTCAAGATATTGTGTTTGGAGAGGTGGATAGATAG

rpl12

ATGAGACCAGGGAGAGCAAGAGCACTTAGACAATTCACTTTGAGTACAGGAAAGTCTGCTGGTAGGAATTCCTCAGGGCGTATTACGGTTTTTCACCGAGGGGGTGGCTCGAAGCGATTGCTGCGAAGA
ATTGATCTGAAACGAAGCACTTCCTCTATGGGCATTGTAGAGAGTATAGAATATGACCCTAATCGTTCTTCTCAGATCGCTCCAGTACGATGGATCAAAGGGGGCTGCCAGAAAAAAATGAACACGATC
GAGAAGTTCGCTCCGCCGCGCAAGATCCTCGAACCTACCACGAACACCATCAGCGGCCTCTTTTCGTTCTCTTTCCTGCCCGGGAAGGTGGATAAAAGAAAGGTAGCTTGCTTCTCTCCTGGACTGATG
GCCGCTTATGTAGTGGTCGGCCTTCCTACCGGAATGCCTCCTTTGTCTTCGTCTAAGAGCGCCTTTGCTAGTAAGGGCGCAGGAAGCACAAAAACTTTAGTGAAGGACGTCTTCTTCTCTGCCTTCTCC
TCTCCAAAGGCCAAGAGAGAGACTGCATCCCTTGCCTTCGCTAGCTCTTTTGGTTTCCCAAGGATAGCGGTAGCTGGGGCAAAGCCCGCTTTCTTCGCTCCGCGAATGAGACAGAAAGTGAGAGGAAAA
AGCACGTTCTCTCTTTGCGAGGTCCAAAAGGGGAGAACGCATAGCATTCTCTGGGCACATAGGATtAAAGGTAAAGCAGGGCTTTCTTGGCAGAGTTTTAGGCGGCAAGATACTTTAGGGCTTGTTGGA
GCTGCTGGGCATAAAAAATCGAAGCCGAAGACGGATCAAGGTAACTTGCCTGCCAAGCCAATAGGCGAAAGGGCGAAGCAACTCAAAGCTCTCCGGGGTTTGAGGGCGAAGGATGGAGCGTGCAAAGTC
GATCGTGCACCTGTcgtgtgaccCGTTGGTCCTAAGCAATGTCTTGCGCGAAGCGACCCACCTAGAAAGAGCTCTCCTTTATCTGGGGGCACTAAAATGAAACTTCGATCAGATGCGGGTATAAAATCC
CGCCGCTGAGATGTCCAGCGGATTCCTGAGCCTTGACGAAAGGTCGGCCACCTTTTTTTTACGGAGAGCAAAAGGCCCGGGGCATAGCAGGATGAACCAATGTGAATGAGTGTAAGCTTCGTTGCCCGA
ACACGATTGGTGCTGACCACACTAGGTGCTACCGCGGTAGCAAGAGAGGCCAGGCAATGACAATTGAGAGGTTGTCACTGAACATTTCTAGTCACACGGGAAGAGAGGTCCAATGGCAAGGCCATACGC
CCGTTTGGCTCCTCGCGGAGTATAGCTCACATCCAAATATCATATCTGATTGGGGAACGGGGCAACACCCATGAAGCTCCGACGGAAAGGGAAGGCCTGCCAGGCCGTATGCCCATGGGTGCAGGATTC
TTCGAAAAAGCGCGGGCTGACTCGGAGACCTGGGACCTTGGCTTAGCAACGAATGAATATTTCTCCTCGAGCTTTCTCCGCCAGCGGCTTATGTAGTGATCGGCCAGCTCGCTAAGCTTTCCTTCTTGT
AGTCGGCCCGTAATGCCTCCCTTCATTTGCTTGCCTCCTTCCTTACTTTTCAGAGAAGCATTTTACGACTATAAGGGGCGCTGTTCACCTTTGGAAACTTAGCTACACCGGTCACGATATCTTGTTGAT
ATTGATTGAGGACTTTCGCTGACTAAATCCATAAACCTAGAAAGTCACCGTCACTGGTACTTTTTTGACTCGATAGGTAGGTATTGGTGGAGCTTGCGTAATGTAGTTGTAGTTAAGGTTGCATTGAAG
TCTTTCTTTTTTTTGAAGATCTACTGAACAAAGGCGAACGGGGTTCCCAAGGCGGGACGTCTGGCAGAATGCTTGGCCTCCCGCGCTGGAAGCGAGACCCGAAGGGTGAGCTTCTGGCGGTTAGCTTCT
AGAACTTATAATAGGCATTAGGCATTCTGAGCTGGAAGGAGGCAAGCAAAAGGCCGACCACTCTATCATTTCCCATTTCTGATGGGAAGGCCGACCACTACACGGACTCTATAACAAGTCATGAGCGAT
ATCGAAACCAAGCCAAAGCCTATAGGCTTTGGGATGAAAGCCAGCCCGACGAAGGCCTATGATAGAGTAAGAAAAAAAGTACGTTAAAGTGACGAAGTAACTTAGCCGTCTACAAAGGGAAAGGCGTCG
GTACGGAGTCACCGTCAGCTGTGGATATAGACTATACTATAAGGAACGGAGTCTTAAACTATGGACCGAGACTACACTAAGGAACAAGGAAGCTTGACTGAGCAAAGAAGTCAAGGAACGAAGCTGCTT
CTCTAATAGCCCCGTTGAATAGGAGGGCGAAGGCTTTAAAAAAAAGTTTGATTTAGGGAGAGGGGGCTTCAAGTTCTTAGGAAGAGCCGTACGAGGCAGCTCACGTACGGTTCGGGAGCCGAGCCCCTg
cacaggggcttaggtcaaCACTTATATAATAGCCAGTCATCAATTAGAAGCAGGCAAAATGGTGATGAATTGCGATTGGTCCAAACCTTCGACCAGCTCCTTCTTGCAATCCGCCCAGAATGACCATCC
TAAGCCCTTATTCACTGTGTGA

rps3

ATGGCACGAAAAGGAAATCCGATTTCGGTAAGACTTGGAAAAAATCGTAGTTCAGATTCAAGTtGGTTCAGTGAGggcgaccgcgaaagtcaccgaatgggtaagtcttttccttcagtttatcctatattttcaaaaaagcgtg
ggaggacgttgcagatgtccgggacggacacgacttcttctaacgctagaagaccactggaaaacacccggaaccagagcatgtcgtgaaagaagcacattggatgggcgtgcttcgaccgtgtctccggggcacagttgaatgt
agatatcattaacgcgaggtgtaggataccaggccgagaaacccgggcaactaaccccccctatgcgccgatcgctagcgcatccaggtccccggcgcccagctctgcgggggaggcctagtctgatctgagaagtgctaattcg
gttcggatagacttcaaaaaagaacgccgccgcccaataagaaaaatcaaacaagtgctaagtttcagatagtgaataaaccgaaacgaaagaagagaggtttctcgctcggcgcctaaagcgcactatgctccgttttaacaaa
tgagagttttcggtggaaccggtgaaccacgcgagctggttagatgtgtgggacagagggctcgtagtacctgttagcgtagctatgcagtggaagggaaggagcctaaatcgaccccttttcttcttttttttttctttgaaaaaa
agcagtacaaagagattagactctcggatgagactaagcagccaccgaccgttccgacggcacccctgaccgattttgattaagagaccgaaaacctatctaaaatgaagcctagtttaagctgtcaaacaaatgatagaattga
aatagaaaatattccgggaataaatatagatagaattttgctcaataaagggaagattcgtagaaaaggataagttgtttcgctaaggctgcaatctttctaaagaaacaagcgagaaactttactttgagaaaaaaaggtgctt
gttgccggtaagtaagcttgttttatatcaataaaggcgaaaggttaatgacaactgcctcttcctgttgcgagggccttgcacgaaaccaaaccgcccccccgccccaatcaagtaccagttgcttcgcagcgcgggtagcctac
ttaggttaggagggcattgccctcagttcttactaacctccggtgtgtaatcaacatgttgctagcttcaactcggttgaataagaatcattgattctctctcctgtccatttcttattcattcagggcgctcggccggtgctcc
tttctcaaaccaaaacgactctgaacgttagggaagataagaccacctgagcgaaccgatcaaagggacttgacttatccgaaccgaacgatagcagcttaaacttaaagctaagcctctcacgagcagcgtgctgcgcggggag
gagcatctcaaagtatggggcaaaggatcaagcgcttttactttgtctcccgggatccaccacaggttgggtttgagagccgtgtgatgggtgactatccagcacggttcggagagcacttttagtctttgttggtgaatggaag
cccccctatcaagcaaaaaataagcggctctttctacggcggggtcactattgactctattTATTATTATGGTAAATTTGTGTATCAAGATGTCAATCTGAGATCTTATTTtGGTTCGATACGTCCACCTACGAGACTCACCTTC
GGCTTTCGTCTCGGTAGGTGTATTATTtTACATTTTCCTAAAAGAACATTCATTCATTTCTTTCTTCCCCGTCGACCACGACGACTGAAACGACGTGAAAAAACTAGACCCGGAAAGGAGAAGGGCCGGTGGTGGACGACATTCG
GGAAAGCCGGGCCGATCGAGTGTCTTCATTCAAGCGACGATACAGAAGAAGAACGAAACGAAGTGAGAGGCCGGGGGGCAAGGAAAAGAGTCGAGTCGATCAGGCTCGACGACCGAAAAAAGCAAAACGAAATCCGGGGTTGGCC
GAAAAAAAAACAACGCTATGGATACCATGACCGATTACCCTCGATAAAGAAGAATCTTTCTAAATtACTTCGGATCAGCGGGGCCTTCAAGCATCCGAAATACGCCGGGGTTGTAAATGACATAGCGTTCCTGATAGAAAATGAC
GACTCCTTtAAAAAAACGAAGTTATTCAAGTTATTTTTCCAAAATAAGTCCCGCTCCGACGGCCCGACGAGTTATCTACGGACCCTCCCTGCAGTGCGCCCCTCCTTGAATTTTTTGGTCATGCAATACTTTTTTAATACAAAGA
ACCAAATTAATTTCGACCCCGTCGTAGTTCTCAATCATTTCGTGGCACCGGGCGCCGCTGAACCATCTACGATGGGGAGAGCGAATGCACAGGGAAGAAGCTTACAGAAGAGAATACGTTCTCGTATCGCTTTTTTTGTAGAAAG
CTtGACCAGCGAGAAAAAGTGTTTGGCCGAAGCCAAAAATAGGTTGACCCACTTCATTCGCTTGGCGAATGATCTTCGCTTCGCGGGAACAACTAAAACCACCATCTCGCTCTTTCCATTCTTCGGTGCTACCTTTTTCTTTCTA
AGAGATGGGGTTGGGGTGTATAATAACCTTGATGCTCGGGAACAACTCCTCAATCAATTAAGGGTCAAATGTTGGAACCTCGTGGGTAAGGATAAGATAATGGAATTGATAGAGAAATTAAAAAACCTAGGTGGGATAGAAGAAT
TGATAAAGGTAATAGATATGATGATAGAGATCATACTGAGAAAGAGAGGAATTCCGTACAGGTACAACTCTTATTTTTACGAAGTCAAAAAAATGCGATCTTTCTTGTCTAATAGAACAAACACTAAGACCTTAATTGAGTCAGT
CAAAATAAAATCTGTTTATCAAAGtGCTTCTCtGATTGCTCAAGACATCTCTTTTCAACTGAAGAACAAAAGAAGATCATTTCATTCCATTTTTGCTAAAATAGTGAAGGAGATTCCAAAAGGGGTGGAGGGAATCCGTATATGT
TTTTCtGGTCGATTAAAAGACGCAGCAGAAAAAGCTCAAACTAAATGCTATAAGCATAGAAAAACTTCTtGTAATGTATTTAACCATAAAATCGATTATGCTCCTGtGGAAGTATtTACTCGTTACGGAATCTtAGGTGTCAAAG
TGTGGATTTCATATAGTCAAAAAAAAGGGAGACGTGCTATATCCGAAACGTACGAAATATAG

Table S2. Identification of nMAT2-associated mitochondrial RNAs by RNA-co-immuno-precipitations / cDNAs sequencing.
cDNAs corresponding to RNAs rthat were co-purified with the nMAT2-PMH2 particles were obtained by RT-PCRs with specific oligoes to different intron-containing pre-RNAs in Arabidopsis mitochondria (see Table S6). The PCR products were cloned into the pGEM-T vector system II (Promega) and sequenced (The Center for Genomic Technologies at the Hebrew University of Jerusalem). The resulting sequencing data is underlined on the corresponding sequences of the 23 group II introns in *Arabidopsis thaliana* (Col-0) mitochondria (Unseld *et al.* 1997). Red letters indicate to C-to-U RNA editing events.
