# Supplementary material for: Analysis of the Roles of the Arabidopsis nMAT2 and PMH2 Proteins Provided with New Insights into the Regulation of Group II Intron Splicing in Land-Plant Mitochondria
Source: Int J Mol Sci. 2017 Nov 17;18(11):2428. doi: 10.3390/ijms18112428 (PMC5713396; doi:10.3390/ijms18112428)
Supplement: Supplementary file 1 [file ijms-18-02428-s001.zip › Table S3 -antibodies.pdf]

**Table S3. List of antibodies used for the analysis of *nmat2 and pmh2* mutants**

| Antibody | Protein I.D. | origin | serum | dilution | Reference / source |
|---|---|---|---|---|---|
| 18-kDa | 18-kDa subunit (NDFSU4) of complex I | *Arabidopsis thaliana* | Rabbit (polyclonal) | 1/5,000 | (Meyer *et al.* 2011, Meyer *et al.* 2009) |
| AOX1/2 | Alternative oxidase subunits 1/2 | *Sauromatum guttatum* | Mouse (monoclonal) | 1/500 | (Elthon *et al.* 1989) |
| AtpA | Mitochondrial ATP-synthase subunit α | *Zea mays* | Mouse (monoclonal) | 1/5,000 | (Michael *et al.* 1993) |
| AtpB | Mitochondrial ATP-synthase subunit β | *Zea mays* | Mouse (monoclonal) | 1/5,000 | (Michael *et al.* 1993) |
| CA2 | γ-carbonic anhydrase-like subunit 2 | *Arabidopsis thaliana* | Rabbit (polyclonal) | 1/1,000 | (Perales *et al.* 2005, Sunderhaus *et al.* 2006) |
| Cox2 | Cytochrome oxidase subunit-2 | *Arabidopsis thaliana* | Rabbit (polyclonal) | 1/5,000 | Agrisera antibodies, AS04 053A |
| HA | Hemagglutinin protein | synthetic peptide | Rabbit (polyclonal) | 1/500 | Sigma (H6908) |
| Nad9 | NADH-dehydrogenase complex subunit-9 | *Triticum spp.* | Rabbit (polyclonal) | 1/50,000 | (Lamattina *et al.* 1993) |
| nMAT2 | Nuclear-encoded Maturase 2 | *Arabidopsis thaliana* | Rabbit (polyclonal) | 1/200 | (Keren *et al.* 2009) |
| PMH2 | Putative mitochondrial RNA helicase 2 | *Arabidopsis thaliana* | Rabbit (polyclonal) | 1/1,000 | (Matthes *et al.* 2007) |
| RbcL | Large subunit of chloroplast Rubsico enzyme | *Pisum sativum* | Rabbit (polyclonal) | 1/1,000 | Gift of Prof. Michal Shapira, BGU |
| RISP | Rieske iron-sulfur protein | *Arabidopsis thaliana* | Rabbit (polyclonal) | 1/5,000 | Gift of Prof. Ian Small, UWA |
| SHMT1 | Serine hydroxymethyltransferase 1 | *Spinacia oleracea* | Rabbit (polyclonal) | 1/1,000 | Agrisera antibodies, AS05 075 |
| VDAC (Porin) | Voltage-dependent anion channel | *Zea mays* | Mouse (monoclonal) | 1/5,000 | Thomas Elthon collection, PM035 |

**References**


**Elthon, T.E., Nickels, R.L. and McIntosh, L.** (1989) Monoclonal antibodies to the alternative oxidase of higher plant mitochondria. *Plant Physiol*, **89**, 1311-1317.

**Keren, I., Bezawork-Geleta, A., Kolton, M., Maayan, I., Belausov, E., Levy, M., Mett, A., Gidoni, D., Shaya, F. and Ostersetzer-Biran, O.** (2009) AtnMat2, a nuclear-encoded maturase required for splicing of group-II introns in Arabidopsis mitochondria. *RNA*, **15**, 2299-2311.

**Lamattina, L., Gonzalez, D., Gualberto, J. and Grienenberger, J.-M.** (1993) Higher plant mitochondria encode an homologue of the nuclear-encoded 30-kDa subunit of bovine mitochondrial complex I. *Eur J Biochem*, **217**, 831-838.

**Matthes, A., Schmidt-Gattung, S., Köhler, D., Forner, J., Wildum, S., Raabe, M., Urlaub, H. and Binder, S.** (2007) Two DEAD-Box Proteins May Be Part of RNA-Dependent High-Molecular-Mass Protein Complexes in Arabidopsis Mitochondria. *Plant Physiol*, **145**, 1637-1646.

**Meyer, E.H., Solheim, C., Tanz, S.K., Bonnard, G. and Millar, A.H.** (2011) Insights into the composition and assembly of the membrane arm of plant complex I through analysis of subcomplexes in Arabidopsis mutant lines. *J Biol Chem*, **286**, 26081-26092.

**Meyer, E.H., Tomaz, T., Carroll, A.J., Estavillo, G., Delannoy, E., Tanz, S.K., Small, I.D., Pogson, B.J. and Millar, A.H.** (2009) Remodeled respiration in ndufs4 with low phosphorylation efficiency suppresses Arabidopsis germination and growth and alters control of metabolism at night. *Plant Physiol*, **151**, 603-619.

**Michael, H.L., Arnost, H. and Elthon, T.E.** (1993) Monoclonal Antibodies to the a- and b- Subunits of the Plant Mitochondrial $F_1$-ATPase. *Plant Physiology*, **101**, 931-937.

**Perales, M., Eubel, H., Heinemeyer, J., Colaneri, A., Zabaleta, E. and Braun, H.-P.** (2005) Disruption of a nuclear gene encoding a mitochondrial gamma carbonic anhydrase reduces complex I and supercomplex I+III2 levels and alters mitochondrial physiology in Arabidopsis. *J Mol Biol*, **350**, 263-277.
**Sunderhaus, S., Dudkina, N.V., Jansch, L., Klodmann, J., Heinemeyer, J., Perales, M., Zabaleta, E., Boekema, E.J. and Braun, H.P.** (2006) Carbonic anhydrase subunits form a matrix-exposed domain attached to the membrane arm of mitochondrial complex I in plants. *J Biol Chem*, **281**, 6482-6488.
