# Supplementary material for: Analysis of the Roles of the Arabidopsis nMAT2 and PMH2 Proteins Provided with New Insights into the Regulation of Group II Intron Splicing in Land-Plant Mitochondria
Source: Int J Mol Sci. 2017 Nov 17;18(11):2428. doi: 10.3390/ijms18112428 (PMC5713396; doi:10.3390/ijms18112428)
Supplement: Supplementary file 1 [file ijms-18-02428-s001.zip › Table S4 - T-DNA oligos.pdf]

Table S4. Oligonucleotides used in screening of individual T-DNA insertion lines in Arabidopsis.

Gene target	Gene I.D.	oligo name	Oligonucleotide sequence (5'-to-3')
nMAT2	At5g46920	GT11F	CTTGATCATGTTTTGTGTCGC
		GT11R	TGATGTTGTAAAAACTAGAATTGAAAAC
PMH2/AtRH53	At3g22330	PMH2FLR5	GCTTTCTCCTTTTACCTCGAACC
		PMH2-Rev	GCGGATCCCAAGCCCATCACCACCAAC
actin2/8	At3g18780	Act2-F	TCTTCCGCTCTTTCTTTCCAAG
		Act2-R	CTGGCGTACAAGGAGAGAAC
SALK T-DNA right border	pROK2 T-DNA	RB-10064	CCAGATCCGGTGCAGATTATTTG
SALK T-DNA left border	pROK2 T-DNA	LB-6443	CATCGCCCTGATAGACGGTT
SAIL T-DNA left border	pDAP101 T-DNA	SynLB	TAGCCTTGCTTCCTATTATATCTTCC
SAIL T-DNA right border	pDAP101 T-DNA	SynRB	GATCAGATTGTCGTTTCCCGCCTTC
