# Supplementary material for: Analysis of the Roles of the Arabidopsis nMAT2 and PMH2 Proteins Provided with New Insights into the Regulation of Group II Intron Splicing in Land-Plant Mitochondria
Source: Int J Mol Sci. 2017 Nov 17;18(11):2428. doi: 10.3390/ijms18112428 (PMC5713396; doi:10.3390/ijms18112428)
Supplement: Supplementary file 1 [file ijms-18-02428-s001.zip › Table S5 - qPCR primers.pdf]

Table S5. Lists of oligonucleotides used for the analysis of the splicing profiles of wild-type and mutant plants by RT-qPCR experiments.

Gene	Forward primer	genome position	Reverse primer	genome position
rpl2	CCGAAGACGGATCAAGGTAA	155542..155561	CGCAATTCATCACCATTTTG	(157249..157268)
rpl2 intron exon2	TTAGGAAGAGCCGTACGAGG	157142..157161	CGCAATTCATCACCATTTTG	(157249..157268)
rps3	AGCCGAAGGTGAGTCTCGTA	26990..27009	CCGATTTCGGTAAGACTTGG	(28696..28715)
rps3 intron1 exon2	AGCCGAAGGTGAGTCTCGTA	26990..27009	TCTACGGCGGGGTCACTAT	(27088..27106)
cox2	TGGGGGATTAATTGATTGGA	40518..40537	TGATGCTGTACCTGGTCGTT	(42016..42035)
cox2 intron1 exon2	TGGGGGATTAATTGATTGGA	40518..40537	AGCAGTACGAGCTGAAAGGC	(40640..40659)
ccmFc	GTGGGTCCATGTAAATGATCG	51765..51785	CACATGGAGGAGTGTGCATC	(52856..52875)
ccmFc intron1 exon1	CCCGGATCGAATCAGAGTT	52747..52765	CACATGGAGGAGTGTGCATC	(52856..52875)
nad1 exon1-2	GACCAATAGATACTTCATAAGAGACCA	289014..289040	TTGCCATATCTTCGCTAGGTG	(318039..318059)
nad1 intron1 exon2	GACCAATAGATACTTCATAAGAGACCA	289014..289040	CGTGCTCGTACGGTTCATAG	(289133..289152)
nad1 exon2-3	ATTCAGCTTCCGCTTCTGG	287943..287961	TCTGCAGCTCAAATGGTCTC	(289034..289053)
nad1 intron2 exon2	GGTTGGGTTAGGGGAACATC	288936..288955	TCTGCAGCTCAAATGGTCTC	(289034..289053)
nad1 exon3-4	AAAAGAGCAGACCCCATTGA	147026..147045	TCCGTTTGATCTCCCAGAAG	(287955..287974)
nad1 intron3 exon4	AAAAGAGCAGACCCCATTGA	147026..147045	GGGAGCTGTATGAGCGGTAA	(147113..147132)
nad1 exon4-5	AGCCCGGGATCTTCTTGA	143401..143418	TCTTCAATGGGGTCTGCTC	(147030..147048)
nad1 intron4 exon5	AGCCCGGGATCTTCTTGA	143401..143418	ACGGAGCTGCATCCCTACT	(143482..143500)
nad2 exon1-2	GCGAGCAGAAGCAAGGTTAT	80109..80128	GGATCCTCCCACACATGTTC	(81259..81278)
nad2 intron1 exon2	GCGAGCAGAAGCAAGGTTAT	80109..80128	CCCATTCCTAACCAGTGGAG	(80272..80291)
nad2 exon2-3	AAAGGAACTGCAGTGATCTTGA	332947..332968	AATATTTGATCTTAGGTGCATT TTC	(79761..79785)
nad2 intron2 exon2	CCCGATCCGATAGTTTACAA	79641..79660	AATATTTGATCTTAGGTGCATT TTC	(79761..79785)
nad2 exon3-4	GCGCAATAGAAAGGAATGCT	330240..330259	CTATGGGTCTACTGGAGCTAC CC	(333071..333093)
nad2 intron3 exon4	GCGCAATAGAAAGGAATGCT	330240..330259	GGCGAATTTCAAACTTGTGG	(330377..330396)
nad2 exon4-5	CAAAGGAGAGGGGTATAGCAA	327932..327952	TATTTGTTCTTCGCCGCTTT	(329793..329812)
nad2 intron4exon4	CTTATTCGTGGCAACCTTCC	329705..329724	TATTTGTTCTTCGCCGCTTT	(329793..329812)
nad4 exon1-2	ATTCTATGTTTTTCCCGAAAGC	162115..162136	GAAAAACTGATATGCTGCCTT G	(163664..163685)
nad4 intron1 exon2	CCGTATGATGCGGAAGTCTC	163512..163531	GAAAAACTGATATGCTGCCTT G	(163664..163685)
nad4 exon2-3	AATACCCATGTTTCCCGAAG	163967..163986	TGCTACCTCCAATTCCCTGT	(167228..167247)
nad4 intron2 exon3	GCGGAACGACCAGAAAAATA	167110..167129	TGCTACCTCCAATTCCCTGT	(167228..167247)
nad4 exon3-4	TTCCTCCATAAATTCTCCGATT	167577..167598	TGAAATTTGCCATGTTGCAC	(169651..169670)
nad4 intron3 exon4	TCTAGCTTGGTTCGGAGAGC	169498..169517	TGAAATTTGCCATGTTGCAC	complement(169651..169670)
nad5 exon1-2	TGGACCAAGCTACTTATGGATG	141880..141901	CCATGGATCTCATCGGAAAT	complement(142793..142812)
nad5 intron1 exon2	TGGACCAAGCTACTTATGGATG	141880..141901	TTCGCAAATAGGTCCGACT	(141962..141980)
nad5 exon2-3	TACCTAAACCAATCATCATATC	190740..1907610	CTGGCTCTCGGGAGTCTCTT	(140743..140762)
nad5 intron2-exon2	GTACGATCGTGTCGGGTGA	140656..140674	CTGGCTCTCGGGAGTCTCTT	(140743..140762)
nad5 exon3-4	AACTCGGATTCGGCAAGAA	22023..22041	GATATGATGATTGGTTTAGGT A	(190740..1907610)
nad5 intron3-exon4	AACTCGGATTCGGCAAGAA	22023..22041	GCCGTGTAATAGGCGACCA	(22150..22168)
nad5 exon4-5	AACATTGCAAAGGCATAATGA	20695..20715	GTTCCTGCGTTTCGGATATG	(21808..21827)
nad5 intron4 exon5	AACATTGCAAAGGCATAATGA	20695..20715	CCTGTAAACCCCCATGATGT	(20827..20846)
nad7 exon1-2	ACCTCAACATCCTGCTGCTC	132118..132137	AAGGTAAAGCTTGAAGATAAG TTTTGT	(133203..133229)

nad7 intron1 exon2	ACGGTTTTTAGGGGGATCTG	133128..133147	AAGGTAAAGCTTGAAGATAAG TTTTGT	(133203..133229)
nad7 exon2-3	GAGGGACTGAGAAATTAATAGAGTACA	133179..133205	TGGTACCTCGCAATTCAAAA	(134362..134381)
nad7 intron2 exon3	AGTGGGAGAGCCGTGTTATG	134182..134201	TGGTACCTCGCAATTCAAAA	(134362..134381)
nad7 exon3-4	ACTGTCACTGCACAGCAAGC	134718..134737	CATTGCACAATGATCCGAAG	(135963..135982)
nad7 intron3 exon4	TAAAGTGAAGTGGTGGGCCT	135797..135816	CATTGCACAATGATCCGAAG	(135963..135982)
nad7 exon4-5	GATCAAAGCCGATGATCGTAA	136007..136027	AGGTGCTTCAACTGCGGTAT	(137951..137970)
nad7 intron4 exon5	CGGCCAAATGACTACAGGAT	137856..137875	AGGTGCTTCAACTGCGGTAT	(137951..137970)
rpl5B	CAGAAGACCTTTCCGTGCTC	chr5 15904477..15904496	CACTGGAAACCGTGTGTTTG	(15904525..15904 544)
actin2	GGTAACATTGTGCTCAGTGGTGG	chr3 6476501..6476523	AACGACCTTAATCTTCATGCTG C	(6476608..647658 6)
matR	AATTTTTGCGAGAGCTGGAA	231141.. 231160	TTGAACCCCGTCCTGTAGAC	(231313 .. 231294)
18S nuclear rRNA	AAACGGCTACCACATCCAAG	chr3 14198079..14198098	ACTCGAAAGAGCCCGGTATT	(14198179..14198 160)
26S rRNA	GACGAGACTTTCGCCTTTTG	38542 … 38523	CTTGGAGCGAATTGGATGAT	(38387… 38406)
