# Supplementary material for: Analysis of the Roles of the Arabidopsis nMAT2 and PMH2 Proteins Provided with New Insights into the Regulation of Group II Intron Splicing in Land-Plant Mitochondria
Source: Int J Mol Sci. 2017 Nov 17;18(11):2428. doi: 10.3390/ijms18112428 (PMC5713396; doi:10.3390/ijms18112428)
Supplement: Supplementary file 1 [file ijms-18-02428-s001.zip › Table S6 - primers cDNAs.pdf]

Table S6. Oligonucleotides used in the sequencing of mtRNAs.

gene target	oligonucleotide sequences (5'-to- 3')
nad1 intron 1	Forward: GCAACGTAGAAAGGGTCCTG
	Reverse: CAAGGAGAGGTTCACCATGA
nad1 intron 2	Forward: TCGAAATATGCCTTTCTAGGAG
	Reverse: AACGGGCGGCATCGTTGTA
nad1 intron 3	Forward: GTCATGGCGCAAAAGCAGATATGG
	Reverse: TGGTTCCCAAGGATCCAATAT
nad1 intron 4	Forward: TCTTCAATGGGGTCTGCTCT
	Reverse: TCACCTTTCAATGGCTTCCT
nad2 intron 1	Forward: AGCGGTTTTCCCAGAGATC
	Reverse: TCCAAGAGGAATTGTGCTATG
nad2 intron 2	Forward: TTGCCAAATCTTTTTATTATTAAG
	Reverse: CTGATCCGGTCGAGGTGG
nad2 intron 3	Forward: AAGATTTTGACTGGATACGAA
	Reverse: TTATAAAAGAGGAACTCGAGCT
nad2 intron 4	Forward: CACTACTAATTGGTATCTTTAT
	Reverse: GACTTCGCCGTTCTTTCCC
nad4 intron 1	Forward: TTATGGGAAAGAGTATATTATAG
	Reverse: GACCCTATTCTTGAACCCTC
nad4 intron 2	Forward: TAAGTGATGTTAGAAAGCCGT
	Reverse: GGAAATCAGGTTTTAAATTTCCA
nad4 intron 3	Forward: TTTGTGCGCCCTTTACCGT
	Reverse: TCAATGAAATTTGCCATGTTGC
nad5 intron 1	Forward: AGGATCAGAAGGAAGCGCTA
	Reverse: GTGCTTAGGCCCCTTCTTC
nad5 intron 2	Forward: TATTACTTTTGCAGGAGCTATG
	Reverse: GAGAACGAAAGCTTTAGTTTTG
nad5 intron 3	Forward: GATATGATGATTGGTTTAGGTAC
	Reverse: GTTAAGGTTGTTGTACACCTG
nad5 intron 4	Forward: CACATTCCGACGATTGGCC
	Reverse: ACCTTTCTCTCACTCAATAGAT
nad7 intron 1	Forward: ATTTTGGACCTCAACATCCTG
	Reverse: CATAGGGCTTTCTATAAGAGG
nad7 intron 2	Forward: GGGACTGAGAAATTAATAGAGT
	Reverse: GGATCAGAAAGAAGGGGGC
nad7 intron 3	Forward: GCAAAGGATTGGGGATTCAG
	Reverse: AGACTGGTTGTTCTTGGGCT
nad7 intron 4	Forward: GGATTTTGACGTACCAGTAGG
	Reverse: TGCCGCCGCGTTTCCCG
cox2 intron 1	Forward: CAAATCTCTATTTTGGTACAAC
	Reverse: CTATTATCTTTGTAGAGGAACAG
ccmFc intron 1	Forward: GAAGCTCTCGCCTTACCAA
	Reverse: TACGGACGTAGATGATATCAT
rpl2 intron 1	Forward: GGTAAAGCAGGGCTTTCTTG
	Reverse: GGAGCTTCATGGGTGTTGC
rps3 intron 1	Forward: CGGTAAGACTTGGAAAAAATCG
	Reverse: TAAGCAGCCACCGACCGTT
